# Effect of a community-based intervention for sexually transmitted infections on population-level prevalence among youth in Zimbabwe (STICH): a cluster-randomised trial

**DOI:** 10.1016/S2214-109X(24)00373-5

**Published:** 2024-11-14

**Authors:** Chido Dziva Chikwari, Ethel Dauya, Victoria Simms, Katharina Kranzer, Tsitsi Bandason, Anna Machiha, Owen Mugurungi, Primrose Musiyandaka, Tinashe Mwaturura, Nkazimulo Tshuma, Sarah Bernays, Constancia Mavodza, Mandikudza Tembo, Kevin Martin, Constance R S Mackworth-Young, Joanna Busza, Suzanna C Francis, Richard J Hayes, Rashida A Ferrand

**Affiliations:** MRC International Statistics & Epidemiology Group, Department of Infectious Disease Epidemiology; London School of Hygiene & Tropical Medicine, London, UK; The Health Research Unit Zimbabwe, Biomedical Research and Training Institute, Harare, Zimbabwe; London School of Hygiene & Tropical Medicine, London, UK; The Health Research Unit Zimbabwe, Biomedical Research and Training Institute, Harare, Zimbabwe; MRC International Statistics & Epidemiology Group, Department of Infectious Disease Epidemiology; London School of Hygiene & Tropical Medicine, London, UK; The Health Research Unit Zimbabwe, Biomedical Research and Training Institute, Harare, Zimbabwe; Clinical Research Department; London School of Hygiene & Tropical Medicine, London, UK; The Health Research Unit Zimbabwe, Biomedical Research and Training Institute, Harare, Zimbabwe; Division of Infectious Diseases and Tropical Medicine, LMU University Hospital, LMU Munich, Munich, Germany; London School of Hygiene & Tropical Medicine, London, UK; The Health Research Unit Zimbabwe, Biomedical Research and Training Institute, Harare, Zimbabwe; AIDS and TB Unit, Ministry of Health and Child Care, Harare, Zimbabwe; London School of Hygiene & Tropical Medicine, London, UK; The Health Research Unit Zimbabwe, Biomedical Research and Training Institute, Harare, Zimbabwe; AIDS Healthcare Foundation, Bulawayo, Zimbabwe; Department of Global Health and Development; School of Public Health, University of Sydney, Sydney, NSW, Australia; Department of Public Health, Environments, and Society; London School of Hygiene & Tropical Medicine, London, UK; The Health Research Unit Zimbabwe, Biomedical Research and Training Institute, Harare, Zimbabwe; MRC International Statistics & Epidemiology Group, Department of Infectious Disease Epidemiology; Department of Global Health and Development; London School of Hygiene & Tropical Medicine, London, UK; The Health Research Unit Zimbabwe, Biomedical Research and Training Institute, Harare, Zimbabwe; Clinical Research Department; London School of Hygiene & Tropical Medicine, London, UK; The Health Research Unit Zimbabwe, Biomedical Research and Training Institute, Harare, Zimbabwe; Department of Global Health and Development; London School of Hygiene & Tropical Medicine, London, UK; The Health Research Unit Zimbabwe, Biomedical Research and Training Institute, Harare, Zimbabwe; Department of Public Health, Environments, and Society; MRC International Statistics & Epidemiology Group, Department of Infectious Disease Epidemiology; Department of Global Health and Development; MRC International Statistics & Epidemiology Group, Department of Infectious Disease Epidemiology; Clinical Research Department; London School of Hygiene & Tropical Medicine, London, UK; The Health Research Unit Zimbabwe, Biomedical Research and Training Institute, Harare, Zimbabwe

## Abstract

**Background:**

Young people are at particularly high risk of acquiring sexually transmitted infections (STIs). We conducted a trial to investigate the effect of a community-based intervention that included STI screening among youth on population-level prevalence of STIs in Zimbabwe.

**Methods:**

STICH was a parallel-arm, cluster-randomised controlled trial nested within CHIEDZA, a trial of community-based integrated HIV and sexual and reproductive health services for youth in Zimbabwe. STICH was conducted in Harare and Bulawayo provinces with eight clusters in each province, randomised 1:1 to control (existing health services) or to the intervention: community-based screening and treatment for *Chlamydia trachomatis* and *Neisseria gonorrhoeae* (males and females) and *Trichomonas vaginalis* (females only) offered over 12 months to intervention cluster residents aged 16–24 years who were attending CHIEDZA. Outcomes were ascertained through a population-level survey immediately after the intervention period, which included young people aged 18–24 years who lived in randomly selected households in each of the 16 clusters. The primary outcome was population prevalence of any (one or more) of the three STIs; secondary outcomes were prevalence of each of the three STIs. The STICH trial is registered with ISRCTN registry, ISRCTN15013425, and the CHIEDZA trial is registered with ClinicalTrials.gov, NCT03719521.

**Findings:**

From Oct 6, 2021, to March 8, 2022, 6361 randomly sampled young people were recruited into the outcome survey (median age 20 years [IQR 19–22], 3500 female and 2101 male, 3066 in intervention clusters and 3295 in control clusters). 5601 participants were included in the primary outcome analysis (2756 in intervention clusters and 2845 in control clusters). In the intervention clusters, 612 (22·2%) of 2756 participants reported that they had attended CHIEDZA and 298 (10·8%) had been tested for *C trachomatis* and *N gonorrhoeae*. In the control clusters, 113 (4·0%) of 2845 participants had attended CHIEDZA and 40 (1·4%) had been tested for *C trachomatis* and *N gonorrhoeae*. In the outcome survey, the cluster-level geometric mean prevalence of the primary outcome (any of *C trachomatis, N gonorrhoeae*, and *T vaginalis*) was 19·07% (geometric standard deviation [GSD] 1·20) in the intervention arm versus 19·95% (GSD 1·10) in the control arm (risk ratio [RR] 0·93 [95% CI 0·78–1·10]; p=0·35). There was no difference between arms in geometric mean prevalence of *C trachomatis* (12·86% [GSD 1·14] in the intervention arm *vs* 12·94% [GSD 1·15] in the control arm, RR 0·97 [95% CI 0·84–1·11]; p=0·60) or *T vaginalis* (7·06% [GSD 1·48] *vs* 6·20% [1·38], RR 1·09 [95% CI 0·74–1·60]; p=0·66). *N gonorrhoeae* prevalence was significantly lower in the intervention arm, with a 43% risk reduction (geometric mean 1·65% [GSD 1·77] *vs* 2·87% [1·43], RR 0·57 [95% CI 0·34–0·96]; p=0·036).

**Interpretation:**

Our study showed high population prevalence of curable STIs. Community-based STI screening appeared to reduce population-level prevalence of *N gonorrhoeae*, but not of *C trachomatis* or *T vaginalis*, probably due to low intervention coverage. Future research is needed on the effects of screening interventions on morbidity, antimicrobial resistance, and re-infection rates.

**Funding:**

Medical Research Council, Economic and Social Research Council, Department for International Development, National Institute for Health and Care Research, and the Wellcome Trust.

## Introduction

The global incidence of four curable sexually transmitted infections (STIs) remains persistently high, with 360 million cases a year.^1^ The WHO Africa region ranks first in terms of annual incidence, compared with other global regions, with 96 million cases of syphilis, chlamydia, gonorrhoea, and trichomoniasis in 2020.^1^ These STIs are associated with compromised sexual and reproductive health, including pelvic inflammatory disease, chronic pelvic pain, tubal infertility, and increased risk of ectopic pregnancy and preterm labour; STIs are also associated with stillbirths and neonatal infections resulting in pneumonia and ophthalmitis. STIs are cofactors for HIV infection, increasing both susceptibility and infectiousness.^2^

The Global Health Sector Strategies on HIV, viral hepatitis, and STIs call for “a precise focus to reach the people most affected and at risk for each disease that addresses inequities”.^1^ Young people are at particularly high risk of STIs, but there is scarce evidence of the effectiveness of specific interventions in this group, especially in low-income settings.^3^ Management of STIs in such settings currently relies on a syndromic approach that classifies STIs into easily identifiable groups of symptoms and signs (syndromes) and provides treatment for the most common organisms implicated in causing each syndrome. This approach relies on people presenting with symptoms, but more than 70% of curable STIs are asymptomatic and they will remain undiagnosed and untreated, with consequent onward transmission, potentially impeding STI control efforts.^2^

Identifying asymptomatic infections requires proactive screening. Some screening programmes, specifically for *Chlamydia trachomatis*, have been implemented in high-income settings, but no such programmes exist in low-income settings.^4^ The availability in recent years of near-patient or point-of-care diagnostic platforms that require little laboratory infrastructure and have high sensitivity and specificity provides an opportunity to consider screening approaches in high-prevalence but resource-constrained settings.^5^ Screening for STIs coupled with treatment for people who test positive, and partner notification to identify and treat sexual partners, could serve to reduce risk of morbidity, reduce prevalence, and control transmission.

These tests are not without costs, and target groups for screening need to be carefully selected to maximise individual-level and population-level health impact. Young people in Africa are at particularly high risk of HIV and STIs.^6^ Global trends from 1990–2019 showed that young people aged 10–24 years had a higher incidence of curable STIs than older age groups.^7^ Young people are at the start of their reproductive lives and might benefit the most from interventions aimed at preventing reproductive morbidity.^8^

We conducted a cluster-randomised trial to investigate the effect of a community-based STI screening intervention coupled with comprehensive STI management in youth on population prevalence of STIs in Zimbabwe. Young people encounter considerable barriers to access and are infrequent users of health facilities, and delivering services in community-based settings might improve engagement and access.^1^

## Methods

### Study design and participants

The STICH (Sexually Transmitted Infections in CHIEDZA) trial was a parallel-arm cluster-randomised trial nested within the CHIEDZA cluster-randomised trial. The CHIEDZA trial aimed to investigate the impact of providing community-based integrated HIV and sexual and reproductive health services for youth on population-level HIV outcomes. The CHIEDZA trial was conducted in three provinces in Zimbabwe (Harare, Bulawayo, and Mashonaland East). Clusters were defined as geographically distinct areas (separated by natural landmarks to minimise contamination) containing a primary care clinic and a community centre from which services were delivered. Each province had eight clusters randomised 1:1 to the control arm (existing HIV and sexual and reproductive health services, which are largely facility-based) or the intervention arm. The CHIEDZA intervention incorporated HIV testing and management, including adherence support (for those who tested HIV-positive), as well as menstrual health products and information, contraception, condoms, syndromic management of STIs and risk reduction, and general health counselling. Services were offered once per week over 30 months at a community venue in each intervention cluster to intervention cluster residents aged 16–24 years by a multidisciplinary team, and were free of charge. To assess eligibility and prevent contamination, all attendees were asked their age and address to ensure that they lived within the cluster. The CHIEDZA intervention procedures are described in detail in the published trial protocol.^9^

**Figure 1:**
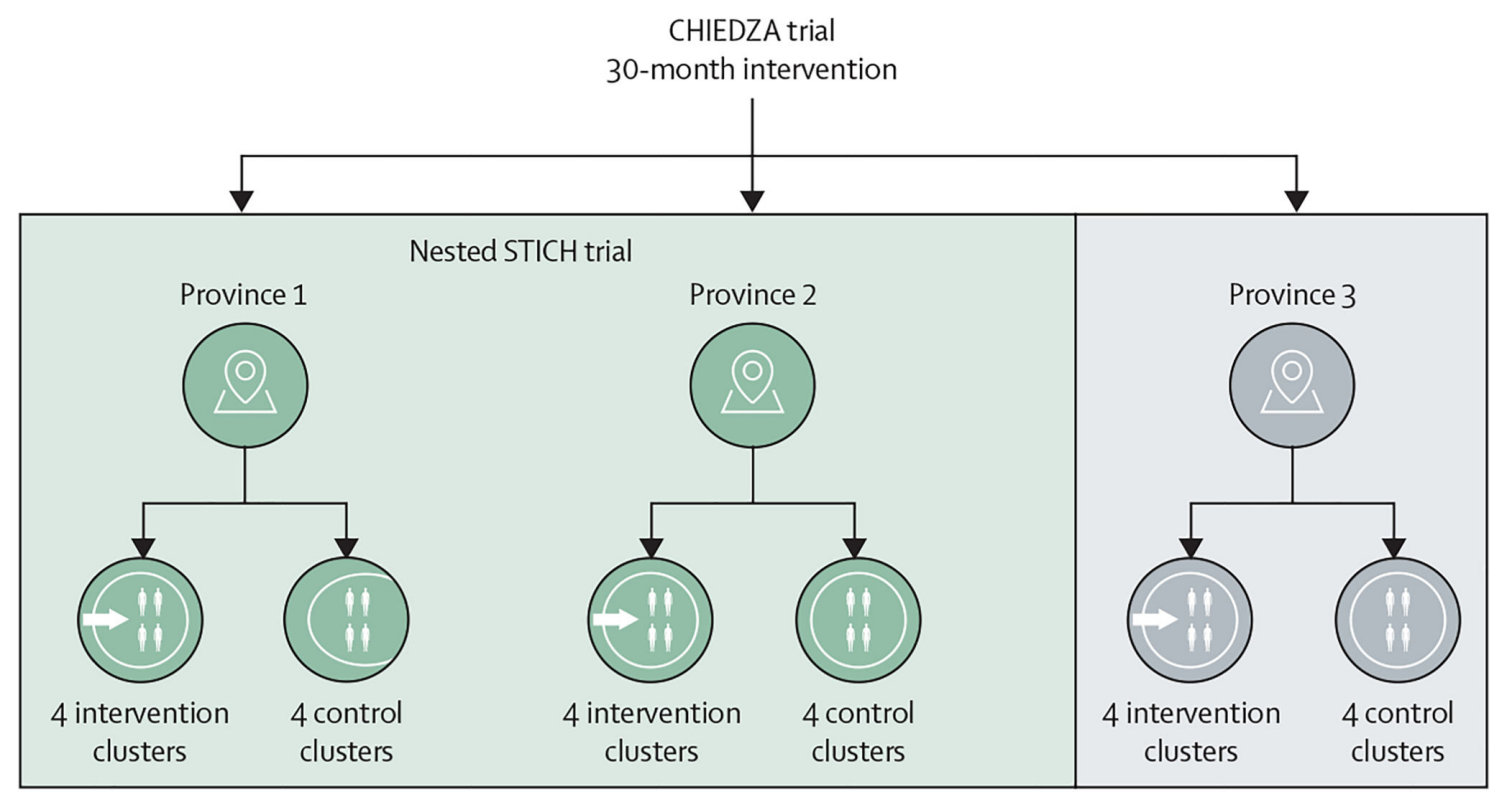
CHIEDZA and STICH trial design

The STICH trial, which aimed to assess the effect of STI screening and treatment on population-level STI prevalence, was done in two of the three provinces in which the CHIEDZA trial was conducted (Harare and Bulawayo, as they were both urban provinces with high population density, which meant that it was logistically easier to implement the STICH trial). A total of 16 study clusters, eight per province, were randomised 1:1 to either the control or the intervention arm, which incorporated the STICH intervention added to the CHIEDZA service package in the final 12 months of the CHIEDZA trial intervention, from Oct 5, 2020, in Harare and Jan 4, 2021, in Bulawayo (figure 1). STI screening was offered to intervention cluster residents aged 16–24 years. Young people aged 18–24 years who lived in randomly selected households in each of the 16 clusters were invited to participate in the prevalence survey.

The CHIEDZA Trial Steering Committee (TSC) was set up in April, 2019 with the overall role to provide independent advice to the Principal Investigator and overall supervision of the trial on behalf of the Sponsor. The TSC, represented by seven members, met anually for the duration of the trial. Ethical approvals for the CHIEDZA and STICH trials were granted by the Medical Research Council of Zimbabwe (MRCZ/A/2387), the Biomedical Research and Training Institute Institutional Review Board (AP149/2018), and the London School of Hygiene & Tropical Medicine Ethics Committee (16124/RR/11602). Verbal consent was obtained for STI testing from intervention clients. Written informed consent from survey participants was obtained. The STICH trial is registered with ISRCTN registry, ISRCTN15013425, and the CHIEDZA trial is registered with ClinicalTrials. gov, NCT03719521.

### Randomisation and masking

Random allocation using coloured balls withdrawn from a bag was made at public randomisation ceremonies in each province for the CHIEDZA trial. As the STICH trial was embedded within the CHIEDZA trial, a separate randomisation was not carried out and the STICH intervention was allocated to the CHIEDZA intervention clusters. Data collectors for the outcome prevalence survey were not blinded to trial arm.

### STICH intervention

The STICH intervention consisted of outreach, promotion, and mobilisation strategies specifically related to STI services, including posters and STI messaging within intervention clusters. Alongside CHIEDZA services, testing for *C trachomatis* and *Neisseria gonorrhoeae* (males and females) and *Trichomonas vaginalis* (females only) was offered to all CHIEDZA clients over a 12-month period. There was no direct linkage between the HIV and the other STI testing services, except that they were all offered as part of the CHIEDZA trial’s package of services. Questions about sexual behaviour were not asked and all clients were offered an STI test regardless of whether they had symptoms. Those who had symptoms were managed syndromically on the same day, according to the Zimbabwe national guidelines, but were also offered an STI test. *N gonorrhoeae* and *C trachomatis* testing was performed on urine samples offsite using the GeneXpert assay (Cepheid, Johannesburg, South Africa), with results provided within 1 week. *T vaginalis* testing was performed on a self-collected vaginal swab using the OSOM lateral flow test (Sekisui Diagnostics, Burlington, MA, USA), with results available within 20 min and same-day treatment provided. All individuals who tested positive for any STI were given treatment free of charge (unless already treated syndromically), given risk-reduction counselling, and offered partner notification slips. Partners who presented to the CHIEDZA trial were also given free treatment regardless of their age and whether they were resident in the intervention cluster. Clients could access re-testing after 3 months following a test, and sooner if they had new or persisting symptoms. Individuals who tested positive for any STI were actively encouraged to re-test after 3 months. Findings (screening uptake, proportion treated, and prevalence in intervention clusters) from the intervention have been published elsewhere.^10^

### Population-level survey and STICH outcomes

The primary outcome of the STICH trial was the population-level prevalence of any (one or more) of *C trachomatis, N gonorrhoeae*, and *T vaginalis*. The secondary outcomes were the prevalence of each of the three individual STIs. Outcomes were measured through a cross-sectional survey of the trial clusters immediately after the end of the intervention period (beginning Oct 6, 2021, in Harare and Jan 4, 2022, in Bulawayo). The survey aimed to recruit a random sample of youth aged 18–24 years in each of the 16 clusters. This age group was selected for the survey to ensure maximum potential for exposure to the trial interventions. Each cluster was divided into road segments of similar length and a random sample of segments was selected. All dwellings in selected segments were visited and the number of households per dwelling and residents per household enumerated. A household was defined as a person or group of related or unrelated persons who live together in the same dwelling or units of a dwelling, who acknowledge one adult male or female as head of the household, who share the same housekeeping arrangements, and who are considered a single unit. All residents aged 18–24 years who were living in a household (defined as having slept in the household for at least 3 nights in the previous week) in dwellings within the selected segment were eligible. If eligible individuals were not at home, the data collectors made up to three return visits on different days.

Eligible individuals were shown a video that explained the survey procedures, including the samples to be collected and the tests to be performed. Consent was recorded on an electronic tablet and participants also signed a paper copy that they retained.

The survey aimed to enrol 700 participants per cluster, to meet the requirements of the CHIEDZA trial within which STICH was embedded.^9^ The sample size for STICH was 300 per cluster. Therefore, data collection days of the survey were randomly allocated to STICH or non-STICH, stratified by weekday (Monday to Friday) or Saturday, with 55% of anticipated days allocated to STICH. It was estimated that data collection would be slower on STICH days (due to the urine collection) and thus 55% of days would be required to obtain 300 (43%) of 700 participants.

All participants (male and female) recruited on the data collection days allocated to STICH were asked to provide a urine sample, which was tested for *C trachomatis, N gonorrhoeae*, and *T vaginalis* using the Seegene Allple STI Essential Assay (Seegene, Seoul, South Korea) at Newlands Clinic laboratory in Harare. Newlands Clinic was responsible for staff training and quality control procedures. Electronic test results and reports were shared with the study data manager daily. All participants who tested positive for any STI were followed up and treated. Survey participants provided a dried blood spot for anonymised HIV antibody testing using the Genscreen ULTRA HIV AG-Ab assay (Bio-Rad Laboratories, Hercules, CA, USA) and referred for diagnostic HIV testing at the nearest clinic. Participants were defined as living with HIV if they either self-reported as HIV-positive or had a positive test result.

## Intervention coverage

Clients who attended the CHIEDZA completed digital fingerprint registration using Simprints scanners (Simprints Technology, Cambridge, UK). Survey participants also completed fingerprint registration, and the two datasets were matched to find the proportion of survey participants who attended CHIEDZA and who received *C trachomatis* and *N gonorrhoeae* testing during the CHIEDZA trial, by arm (representing coverage in the intervention arm and contamination in the control arm).

## Statistical analysis

CONSORT guidelines for cluster-randomised trials were followed. Cluster-level analyses were used to adjust for between-cluster variability, as recommended for trials with fewer than 15 clusters per arm.^11^ Descriptive analysis was used to compare the cluster-level characteristics of the two arms, and the analysis of intervention effect was adjusted for demographic variables that were unbalanced between arms and for province. Imbalance was defined as a difference in distribution between arms likely to affect the results of the trial. All analyses were adjusted, a priori, for sex and age in years.

A complete-case analysis was undertaken with no imputation planned to correct for missing data in the primary and secondary outcomes, as the proportion of missing data was expected to be low. The risk of the three STI infections (any of *C trachomatis, N gonorrhoeae*, or *T vaginalis*) for each cluster was calculated and shown by arm. All participants with a valid test result for the relevant outcome were included. A two-stage analysis was conducted using the *clan* command in Stata 17.0.^12^ In the first stage, a logistic regression model was fitted to estimate the effects on the outcome of the adjustment covariates sex, age, and province. Cluster-summarised observed and predicted statistics were used to calculate ratio-residuals. In the second stage, linear regression of the log ratio-residual on province and arm was used to estimate the risk ratio (RR) and 95% CI for the effect of intervention, with 13 degrees of freedom. Cluster-level geometric means and geometric standard deviations (GSDs) were reported. Significance tests were two-sided with 5% level of significance.

As an exploratory analysis, outcomes were reported by sex (male and female), by HIV status (positive and negative), and among only those who reported that they had ever had sex. For the sex and HIV status variables, effect modification was examined by calculating the cluster-level risk of the outcome separately for the two groups (eg, males and females), and then comparing the mean log RR for the two groups between arms. An unpaired *t* test on the cluster-level differences was used to test for interaction. Men were not eligible for *T vaginalis* testing in the intervention; however, they were tested for *T vaginalis* in the survey. As an exploratory analysis, we assessed the difference between arms in the prevalence of one or both of *C trachomatis* and *N gonorrhoeae* by sex (a modification of the primary outcome, excluding *T vaginalis*).

The nesting of the STICH trial within the CHIEDZA trial imposed restrictions on the size and duration of STICH. Thus, the sample size calculations for STICH were based on the minimum detectable difference in STI prevalence that could be detected with adequate power, rather than on explicit assumptions regarding the coverage of the intervention. The expected prevalence for the primary outcome in the control arm was 17%, based on a pilot survey of 2331 young people in the CHIEDZA trial in 2019. The coefficient of variation in the outcome was conservatively estimated at 0·25–0·30. A sample size of 300 per cluster in 16 clusters gave 80% power to detect an absolute decrease in the outcome of 7% (to 10% prevalence) and 90% power to detect an absolute decrease of 8% (to 9% prevalence) at a coefficient of variation of 0·30. While the sample size per cluster might have been increased to match that of the CHIEDZA trial, in a cluster-randomised trial with a relatively small number of clusters, the variance is dominated by the between-cluster variation, and this is not reduced by increasing the sample size within each cluster. The trial was not powered to detect an effect on secondary outcomes. The between-cluster coefficient of variation was calculated as the between-cluster SD of the outcome divided by the mean across clusters.

### Role of the funding source

The funders of the study had no role in study design, data collection, data analysis, data interpretation, or writing of the report.

## Results

The uptake of STI screening within the intervention clusters and the yield and prevalence of STIs and proportion treated have been reported previously.^10^ In summary, among eligible young people attending the CHIEDZA intervention, 8549 (86·1%) of 9891 were tested for *C trachomatis* and *N gonorrhoeae* and, among those diagnosed, treatment uptake was 60·6% (924 of 1526). Uptake of *T vaginalis* testing among eligible females was 85·2% (6388 of 7501) and, among those diagnosed, treatment uptake was 98·8% (483 of 489). A partner returned for treatment for 103 (5·7%) of 1807 participants who were diagnosed with an STI.

**Figure 2:**
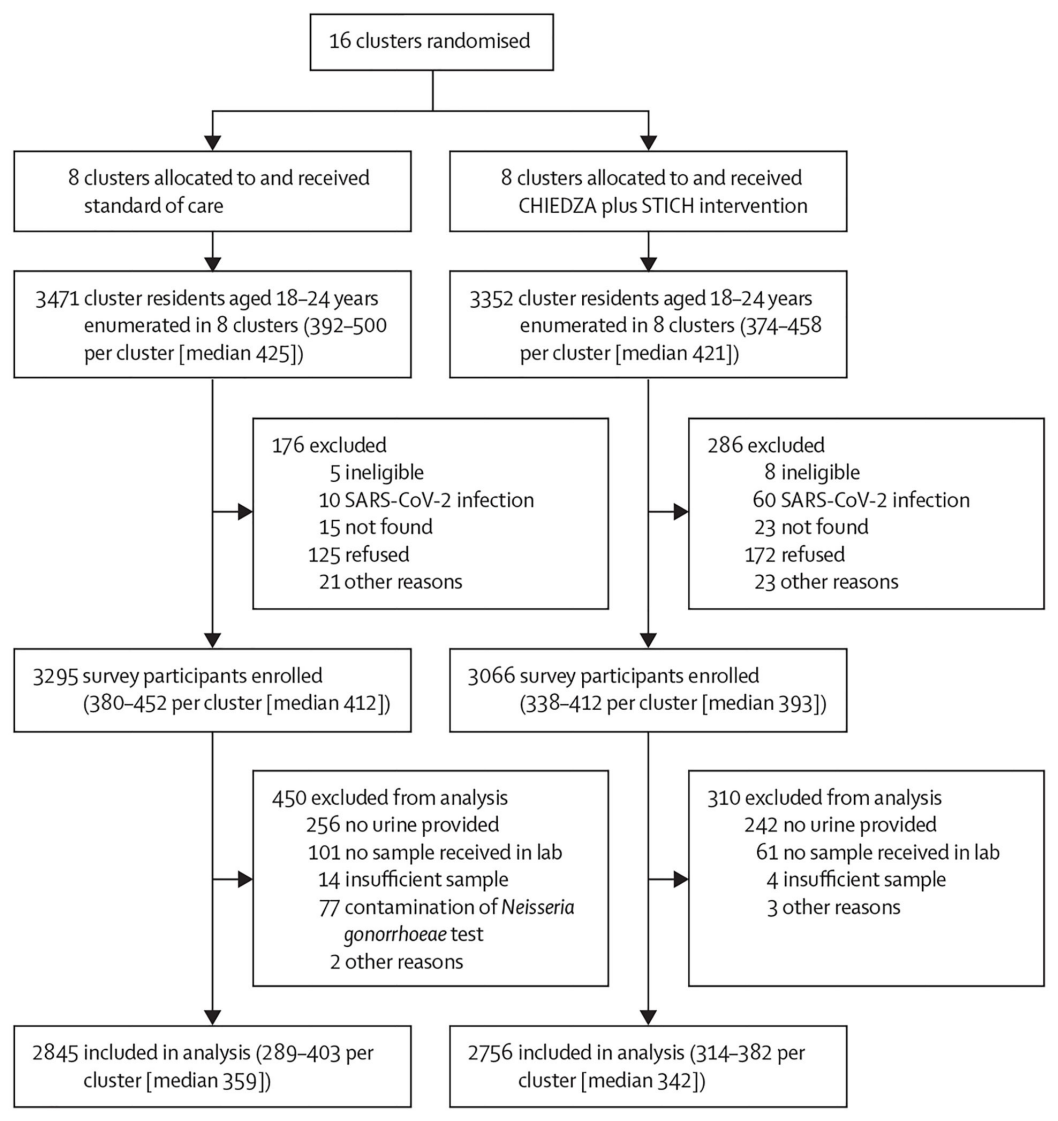
Trial profile

The outcome survey was conducted from Oct 6 to Dec 15, 2021, in Harare province, and from Jan 4 to March 8, 2022, in Bulawayo province. In total, 6823 residents of the trial communities were enumerated on days allocated for STICH sampling, 3471 in the control clusters and 3352 in the intervention clusters (figure 2). Of those enumerated, 176 (5·1%) of 3471 in the control clusters and 286 (8·5%) of 3352 in the intervention clusters were excluded from the survey because they refused, were not found, were ineligible, or had SARS-CoV-2 infection, or for other reasons. In total, 6361 residents (3295 in control clusters and 3066 in intervention clusters) were enrolled. Of those enrolled, 450 (13·7%) of 3295 in the control clusters and 310 (10·1%) of 3066 in the STICH clusters were excluded from analysis of the primary outcome because they did not give a urine sample, the urine sample was not found in the lab, the sample was insufficient or was contaminated, or for other reasons. On two days of data collection (Nov 17–18, 2021), *N gonorrhoeae* results did not pass laboratory quality control, resulting in unusable results for samples collected on those days. Ultimately, 82·1% of enumerated residents were included in the analysis of the primary outcome, 2845 (82·0%) of 3471 in the control arm and 2756 (82·2%) of 3352 in the intervention arm.

Descriptive characteristics of participants, by arm, are shown in table 1. There was imbalance by sex: the proportion of male participants was 34·6% (953 of 2756) in the intervention arm and 40·4% (1148 of 2845) in the control arm. The median age of participants was 20 years (IQR 19–22) and 3500 (62·5%) of 5601 were female. About half the participants (2874 [51·3%] of 5601) were neither in education nor formally or informally employed.

In the intervention arm, 612 (22·2%) of 2756 participants had a fingerprint match against a participant in the CHIEDZA trial database, indicating they had attended CHIEDZA services, and 298 (10·8%) of 2756 had received a *C trachomatis* and *N gonorrhoeae* test. In the control arm, 113 (4·0%) of 2845 participants had attended CHIEDZA services and 40 (1·4%) had received a *C trachomatis* and *N gonorrhoeae* test.

The cluster-level geometric mean prevalence for the primary outcome (*C trachomatis, N gonorrhoeae*, and *T vaginalis*) was 19·07% (GSD 1·20) in the intervention arm versus 19·95% (1·10) in the control arm (absolute numbers 538 of 2756 *vs* 569 of 2845), with no evidence of a difference between arms (RR 0·93 [95% CI 0·78–1·10]; p=0·35; table 2). There was also no difference between arms in the geometric mean prevalence of *C trachomatis* (12·86% [GSD 1·14] in the intervention arm and 12·94 [1·15] in the control arm, absolute numbers 358 of 2756 *vs* 382 of 2922, RR 0·97 [95% CI 0·84–1·11]; p=0·60) or *T vaginalis* (7·06% [GSD 1·48] *vs* 6·20% [1·38], absolute numbers 213 of 2756 *vs* 189 of 2922, RR 1·09 [95% CI 0·74–1·60]; p=0·66). However, the geometric mean prevalence of *N gonorrhoeae* was significantly lower in the intervention than in the control arm, with a 43% risk reduction (1·65% [GSD 1·77] *vs* 2·87% [1·43], RR 0·57 [95% CI 0·34–0·96]; p=0·036). There were 52 of 2756 *N gonorrhoeae* infections in the intervention arm versus 85 of 2829 in the control arm. There was no evidence of a difference between arms in the exploratory outcome of *C trachomatis* and *N gonorrhoeae* prevalence (14·06% [GSD 1·17] *vs* 15·40% [1·15], RR 0·89 [95% CI 0·75–1·05]; p=0·14). In total, 393 of 2756 participants in the intervention arm and 439 of 2839 in the control arm had either *C trachomatis* or *N gonorrhoeae*.

Overall, there were 414 young people living with HIV and 364 of them had STI test results. For *N gonorrhoeae*, there was evidence of significant effect modification by HIV status (test for interaction p=0·02), with a significant effect of the intervention among those who were HIV-negative (RR 0·50 [95% CI 0·29–0·86]; p=0·017), but not among those who were HIV-positive (RR 1·14 [95% CI 0·47–2·80]). This was not observed for the primary outcome or for *C trachomatis* or *T vaginalis* prevalence (table 3).

The RR for *T vaginalis* was 2·00 (95% CI 1·08–3·71) for males and 0·97 (95% CI 0·63–1·50) for females, indicating evidence of increased *T vaginalis* prevalence in the intervention arm for males only, but with only moderate evidence of interaction (p=0·055; table 3). There was no interaction with sex for the primary outcome or for *C trachomatis* or *N gonorrhoeae* prevalence.

On exploratory analysis, there was no difference between arms in the primary outcome among the 4249 participants who reported that they had ever had sex (22·8% in intervention arm *vs* 24·0% in control arm, RR 0·92 [95% CI 0·78–1·10]; p=0·34); the absolute numbers were 472 of 1959 in the intervention arm versus 434 of 1857 in the control arm.

## Discussion

Our trial found no evidence of an effect of the intervention on the population-level prevalence of any of *C trachomatis, N gonorrhoeae*, and *T vaginalis*. This lack of effect on the primary outcome might be explained by the low coverage of STI screening and treatment in the intervention arm, which was unable to reduce the very high *C trachomatis* population prevalence and the consequent high risk of re-infection in the community. However, there was more than a 40% risk reduction in *N gonorrhoeae* prevalence. *C trachomatis* had the highest prevalence and hence the primary outcome (a composite of *C trachomatis, T vaginalis*, and *N gonorrhoeae* prevalence) was driven mainly by *C trachomatis*.

The prevalence of *C trachomatis* (geometric mean prevalence of 19·1% and 20·0% by arm) was much higher than that reported in previous clinic or community-based studies in east and southern Africa (excluding South Africa), which had an estimated pooled prevalence of 2·7% among women aged 15–24 years.^13^ However, the prevalence of *N gonorrhoeae* (geometric mean prevalence of 1·7% and 2·9% by arm) was comparable to the estimated prevalence of 1·7% reported in these studies.^13^ The differential impact of STI screening and management on *C trachomatis* compared with *N gonorrhoeae* can be explained by the difference in prevalence of the two infections. Given the much higher population prevalence of *C trachomatis*, the risk of re-infection in those who were screened and treated in the intervention was likely to be substantially higher than for *N gonorrhoeae* and would thus rapidly dilute any effect of the intervention. Other possible explanations for the absence of an effect on *C trachomatis* include the low population-level coverage of the intervention,^14^ the relatively low treatment uptake (only 60·7% of CHIEDZA clients who tested positive for *C trachomatis* or *N gonorrhoeae* returned for treatment), and the low proportion (5·7%) of partners presenting to CHIEDZA for treatment.^10,15^ Low treatment uptake in the intervention was probably due to the fact that the majority of STIs were asymptomatic, so young people might have felt that returning to the site for treatment was not a priority for them. In addition, taking up treatment required follow-up via telephone and some participants did not have mobile phones. Most participants noted that they often felt ill-equipped to counsel and convince their partners to seek treatment and feared that notifying their partners would expose them to social risks, threatening their emotional and physical safety. We conducted a detailed study exploring partner notification and why acceptability was low among youth.^10,15^ In addition, infections caused by *N gonorrhoeae* are more likely to be symptomatic and lead to health-care attendance and prompt treatment than those caused by *C trachomatis*.^16^ Some strategies to mitigate the above limitations would include the use of point-of-care tests, which would allow youth to be diagnosed and treated on the same day, as well as use of alternative partner notification strategies such as provider-facilitated partner notification and expedited partner therapy.

This study is the first in Africa to report an effect of screening on population-level prevalence of *N gonorrhoeae*. Evidence to date for STI (mainly *C trachomatis*) screening strategies has come from high-resource settings and is mixed.^4^ A 2012 pragmatic trial in the general population in the Netherlands found no difference in *C trachomatis* test positivity after 3 years of postal invitations, but the uptake of testing was low (16·1% uptake after the first invitation).^17^ The implementation of a national *C trachomatis* screening programme in England resulted in large increases in *C trachomatis* testing, with more than 1 million young people aged 15–24 years tested in 2019; however, the proportion of positive *C trachomatis* tests has remained consistent at 10% following a sustained increase from 8% in 2015.^18^

Although *N gonorrhoeae* has a much lower prevalence than *C trachomatis*, it is more likely to be symptomatic and result in pelvic inflammatory disease.^16^ *N gonorrhoeae* is also a considerable public health concern because, globally, *N gonorrhoeae* has developed progressive resistance to different classes of antibiotics.^19^ Following the increase in fluoroquinolone resistance, cephalosporins have been the recommended first-line treatment for *N gonorrhoeae*. Unfortunately, cephalosporin resistance has recently emerged, and multidrug-resistant *N gonorrhoeae* has been reported from many settings at variable prevalence. In the absence of alternative antibiotic classes, this threatens to render *N gonorrhoeae* untreatable. *N gonorrhoeae* has been classified by WHO as an antimicrobial resistance priority pathogen.^19^ An important component of the global action plan to address antimicrobial resistance is to control *N gonorrhoeae* transmission. As such, the Global Health Sector Strategies on HIV, viral hepatitis, and STIs (2022–30) recommend screening approaches in priority populations.^20^ Priority populations differ across settings and countries, and our study provides valuable evidence on the effectiveness of screening to control *N gonorrhoeae* transmission among young people in low-income settings with high HIV and STI prevalence.

Although *N gonorrhoeae* is an important public health problem, a number of other factors need to be considered when contemplating STI screening strategies, as discussed by Wilson and Junger.^21^ Of note, screening (diagnosing and treating a disease earlier) should confer some individual-level benefit even though this might not be the main focus of a control programme. There is limited evidence regarding the beneficial effect of STI screening on prevention of short-term or long-term health outcomes such as pelvic inflammatory disease, compromised fertility, and adverse reproductive outcomes.^22,23^

An important knowledge gap concerns uncertainty about the natural history of STIs, including what proportion of infections are self-limiting, what proportion develop sequelae and when in the course of infection, and whether sequelae develop after a single or multiple infection episodes. Although many observational studies have shown associations with adverse reproductive health sequelae, these might be subject to bias.^24^ The risk of long-term complications attributable to STIs, in particular the risk of tubal factor infertility, is considered low and there is uncertainty about the preventable fraction from screening.^25^ Further controlled studies using reproductive health outcomes as outcome measures are needed to ascertain whether screening results in individual-level benefits and which groups would benefit most from screening. However, such trials are challenging to conduct given that these outcomes are uncommon and there might be long periods between infection and the outcome, requiring large sample sizes and prolonged follow-up. In addition, the potential harms of screening, such as over-treatment, anxiety associated with a positive diagnosis, stigma, and implications for relationships need to be considered. Other key considerations for implementation of screening approaches are health system factors and cost. Costing studies for this trial are underway and will be reported separately.

We found a significant effect of the intervention on *N gonorrhoeae* prevalence among young people who were HIV-negative but not among those living with HIV. Higher *N gonorrhoeae* prevalence was also observed among young people living with HIV who accessed STI testing in the intervention when compared with those who were HIV-negative (8·9% *vs* 2·6%).^10^ The reasons for this are not clear, but might include a higher risk of infection due to riskier sexual behaviour or lower likelihood of spontaneous clearance of infection among people living with HIV.^26,27^

When stratified by sex, the study showed a higher prevalence of *T vaginalis* among males in the intervention arm compared with the control arm. *T vaginalis* testing was not offered to males in the intervention (due to resource constraints, which necessitated prioritisation, with an anticipated low yield of *T vaginalis* among males), which makes it challenging to interpret the between-arm difference in *T vaginalis* among males. Any anticipated differences in *T vaginalis* prevalence in males would probably be due to the indirect effects of *T vaginalis* testing and treatment among females; however, the converse effect was observed. Due to these considerations, including the fact that the test for interaction showed only weak evidence, we conclude that this effect was probably due to chance and should be interpreted with caution.

The strengths of the study are that it incorporated a rigorous cluster-randomised trial design and was well powered with high participation rates. Embedding STICH within CHIEDZA was advantageous in terms of costs and logistical efficiency and, additionally, because in the context of southern Africa, where high STI prevalences coexist with high HIV prevalence and incidence, any community-based intervention to reduce STI prevalence in youth would necessarily have to be linked to relevant HIV services. The combined CHIEDZA and STICH services achieved this important integration of services. Furthermore, the integration of STI screening with provision of other services, as was done in the CHIEDZA trial, could improve uptake and acceptability, and allow for programmatic efficiency. Other services such as condom provision and sexual and reproductive health education might also have a positive effect on STI outcomes. As such, where possible, STI screening services should ideally be part of a holistic package of sexual and reproductive health services, including HIV services. The outcome assessed impact on population-level prevalence, capturing the effects of uptake of both screening and treatment in the intervention, as well as coverage of the intervention among survey participants.

We acknowledge several limitations. Although the intervention had high uptake among youth who attended the CHIEDZA trial, a key limitation of the study was the low coverage of the intervention in the community, as reported by the survey participants, with only 10·8% of survey participants in the intervention arm reporting having had a *C trachomatis* and *N gonorrhoeae* test during the CHIEDZA trial. Although there was active community mobilisation during the CHIEDZA intervention, added to previous formative work with CHIEDZA communities, possible reasons for low coverage of the intervention include high in-migration and out-migration of young people, limited access to the intervention in the community due to young people being unaware of the CHIEDZA trial and available services, opening times that might not have been optimal for young people, distance to the CHIEDZA sites from their homes, and potentially low perceived risk among young people who did not come to the CHIEDZA trial’s services. The study was limited to in-person contacts locally, excluding the use of social media to reach young people in order to avoid possible contamination of the intervention from young people living in control clusters. We note that powering of the study was not based on explicit assumptions about intervention coverage, which would have been difficult to predict before the trial. Even if it had been possible to predict coverage, it would still have been exceedingly difficult to estimate the effect of this on population STI prevalence. This would probably have involved constructing complex mathematical models of STI transmission, which would require detailed data on sexual networks and health-seeking behaviour among young people in Zimbabwe. No such data of this kind were available, and it would have taken a considerable time to collect them and to develop and validate the models needed to estimate intervention effects. Hence, the opportunity to nest the STICH trial in the CHIEDZA trial would have been lost. Mathematical models from Australia suggest that increased population coverage of testing is likely to have led to greater reductions in population prevalence, particularly of *C trachomatis*.^28^

An additional limitation is that approximately 12% of those enrolled in the outcome survey were excluded from analysis, mostly because they did not give a urine sample. When the characteristics of those included in and excluded from the analysis were compared (appendix 1 p 6), those excluded were more likely to have never had sex, to be never married, to be in education, and to have completed form 6 at secondary school. Given these findings, it seems plausible that those who had never had sex did not consider themselves to be at risk for STIs and so were less likely to give a urine sample for testing. As the proportions excluded were roughly similar in both study arms (10·1% in intervention clusters and 13·7% in control clusters) it is unlikely that the primary analyses were much affected by the missing data. However, it is likely that prevalences of STIs were underestimated because of this selection bias.

Furthermore, the trial did not assess the impact of screening on clinical outcomes, and drug susceptibility testing for *N gonorrhoeae* was not performed and so *N gonorrhoeae* antibiotic resistance profiles were not available; both are critical when considering screening strategies. There was a lower proportion of males than females in the outcome survey, a feature of other population-based surveys in the region and mirrored in the trial intervention.^10,29^ Possible reasons include the fact that men are more likely to be at work and are often less likely to participate. We did not observe a differential participation rate by sex and three visits were undertaken to find eligible participants. Enumeration of study communities undertaken as part of the 2020 Zimbabwe Population-based HIV Impact Assessment (ZIMPHIA) surveys in similar study areas confirm that there are fewer men than women, especially among those aged 18–24 years, in these communities.^30^ Exploratory qualitative interviews undertaken by the process evaluation team corroborated this, with out-migration due to exceedingly high levels of unemployment among young males (CRSM-Y; unpublished data). All analyses were adjusted for sex and age, but residual confounding cannot be excluded, particularly given the relatively small number of clusters. Although participation in the survey was generally high, selection bias could have been introduced because not all participants provided urine samples or the sample volume was too small, samples were not received in the laboratory, and results from tests of samples collected on two days (both control arm days) were invalid because they did not pass the quality control standards. Sensitivity of the molecular tests is also lower for urine than for genital samples, possibly resulting in non-differential misclassification and underestimation of prevalence estimates. Conversely, molecular tests do not differentiate between viable and non-viable organisms, and prevalence can therefore be overestimated.^31^ However, this bias would apply equally to both trial arms. The study was conducted in urban and peri-urban settings, thus limiting generalisability to rural settings.

In summary, this was a pragmatic trial designed to measure the effect of a practical community-based STI screening intervention rather than to provide proof-of-concept for STI screening to reduce STI prevalence. Our study showed high overall population prevalence of curable STIs, including frequent co-infections. STI screening for young people delivered in community-based settings appeared to reduce the population-level prevalence of *N gonorrhoeae*, a pathogen that is of particular importance due to growing concerns about antimicrobial resistance, especially the development of multidrug resistance, which might render *N gonorrhoeae* untreatable. Developments in near-point-of-care or point-of-care tests and other technologies, including mobile health and home sampling, offer increased opportunities and options for STI screening. Any STI screening approaches will need to be coupled with acceptable strategies for partner notification and continued emphasis on prevention and education. Finally, any STI screening strategies also need to go beyond population-level control and consider the effect of screening on individual-level morbidity.

## Supplementary Material

Supplementary Materials

## Figures and Tables

**Table 1:** Characteristics of participants by trial arm

	Intervention (n=2756)	Control (n=2845)
**Age, years**		
18–20	1402 (50·9%)	1483 (52·1%)
21–24	1354 (49·1%)	1362 (47·9%)
**Sex (self-reported)**		
Male	953 (34·6%)	1148 (40·4%)
Female	1803 (65·4%)	1697 (59·6%)
**Education level attained**		
Did not complete primary	61 (2·2%)	45 (1·6%)
Completed primary	480 (17·4%)	475 (16·7%)
Completed form 4	1639 (59·5%)	1783 (62·7%)
Completed form 6	358 (13·0%)	340 (12·0%)
Post-secondary	218 (7·9%)	202 (7·1%)
**Sexual debut**		
Ever had sex (penetrative intercourse)	1857 (67·8%)	1959 (69·1%)
Never had sex (penetrative intercourse)	882 (32·2%)	875 (30·9%)
Missing or refused to answer	17	11
**Number of sexual partners in lifetime (if ever had sex)**		
1	744 (41·8%)	628 (33·3%)
2	385 (21·7%)	378 (20.0%)
3	235 (13·2%)	316 (16·7%)
4	121 (6·8%)	141 (7·5%)
5	123 (6·9%)	148 (7·8%)
>5	170 (9·6%)	277 (14·7%)
Did not say	79	71
**Number of sexual partners in past 12 months (if ever had sex)**		
0	128 (7·0%)	131 (6·8%)
1	1311 (71·6%)	1269 (65·4%)
>1	392 (21·4%)	542 (27·9%)
Did not say	26	17
**Condom use (if had sexual partner in past 12 months)**		
Used condom the last time had sex	749 (44·2%)	813 (45·0%)
Did not use a condom the last time had sex	945 (55·8%)	993 (55·0%)
Did not say	9	5
**In the past 12 months, had sex or been sexually involved with anyone because they gave you or said they would give you material support of any kind**		
Yes	32 (1·9%)	46 (2·6%)
No	1669 (98·1%)	1760 (97·5%)
Did not say	2	5
**Household monthly income**		
<US$50	406 (17·1%)	528 (21·3%)
US$50–100	770 (32·4%)	711 (28·7%)
US$101–200	724 (30·4%)	732 (29·6%)
US$201–500	397 (16·7%)	417 (16·8%)
>US$500	81 (3·4%)	89 (3·6%)
Missing	378	368
**Main activity**		
Education	730 (26·5%)	796 (28·0%)
Formally employed	112 (4·1%)	133 (4·7%)
Informally employed	475 (17·2%)	481 (16·9%)
None of the above	1439 (52·2%)	1435 (50·4%)
**Marital status**		
Married or living together	592 (21·5%)	492 (17·3%)
Never married	2061 (74·8%)	2219 (78·0%)
Divorced, widowed, or separated	103 (3·7%)	134 (4·7%)

**Table 2:** Trial outcomes

	Intervention	Control	Cluster-level geometric mean prevalence (GSD)		Adjusted risk ratio* (95% CI)	p value	Between-cluster coefficient of variation
			Intervention (n=8)	Control (n=8)			
**Primary outcome**							
*C trachomatis, N gonorrhoeae,* and *T vaginalis*^†^	538/2756	569/2845	19·07% (1·20)	19·95% (1·10)	0·93 (0·78–1·10)	0·35	0·15
**Secondary outcomes**							
*C trachomatis* ^‡^	358/2756	382/2922	12·86% (1·14)	12·94% (1·15)	0·97 (0·84–1·11)	0·60	0·13
*N gonorrhoeae* ^‡^	52/2756	85/2829	1·65% (1·77)	2·87% (1·43)	0·57 (0·34–0·96)	0·036	0·50
*T vaginalis* ^‡^	213/2756	189/2922	7·06% (1·48)	6·20% (1·38)	1·09 (0·74–1·60)	0·66	0·38
**Exploratory outcome**							
*C trachomatis* and *N gonorrhoeae^§^*	393/2756	439/2839	14·06% (1·17)	15·40% (1·15)	0·89 (0·75–1·05)	0·15	0·14

*C trachomatis=Chlamydia trachomatis*. GSD=geometric standard deviation. *N gonorrhoeae=Neisseria gonorrhoeae.* *T vaginalis=Trichomonas vaginalis*.

* Adjusted for sex and age in years.

† Population-level prevalence of any (one or more) of *C trachomatis*, *N gonorrhoeae*, and *T vaginalis*.

‡ Population prevalence of single infection.

§ Population-level prevalence of one or both of *C trachomatis* and *N gonorrhoeae*.

**Table 3:** Trial outcomes by HIV status and sex

	Intervention	Control	Cluster-level geometric mean prevalence (GSD)		Adjusted risk ratio* (95% CI)	p value	Test for interaction (p value)
			Intervention (n=8)	Control (n=8)			
*C trachomatis, N gonorrhoeae,* and *T vaginalis*^†^	··	··	··	··	··	··	0·68
HIV-positive	47/156	69/208	26·35% (1·81)	30·17% (1·52)	0·85 (0·50–1·44)	0·51	··
HIV-negative	487/2583	497/2612	18·46% (1·21)	18·99% (1·09)	0·94 (0·79–1·11)	0·41	··
*C trachomatis* ^‡^	··	··	··	··	··	··	0·43
HIV-positive	24/156	43/213	16·30% (1·77)	19·81% (1·70)	0·81 (0·45–1·43)	0·43	··
HIV-negative	333/2583	338/2684	12·79% (1·14)	12·49% (1·16)	0·99 (0·86–1·14)	0·89	··
*N gonorrhoeae* ^‡^	··	··	··	··	··	··	0·016
HIV-positive	9/156	9/205	6·44% (2·54)	5·65% (2·16)	1·14 (0·47–2·80)	0·75	··
	43/2583	76/2599	1·43% (1·84)	2·82% (1·39)	0·50 (0·29–0·86)	0·017	··
*T vaginalis* ^‡^	··	··	··	··	··	··	0·84
HIV-positive	28/156	32/213	16·58% (2·25)	15·15% (1·46)	1·04 (0·58–1·86)	0·89	··
HIV-negative	182/2583	155/2684	6·48% (1·45)	5·60% (1·32)	1·10 (0·76–1·58)	0·60	··
*C trachomatis, N gonorrhoeae,* and *T vaginalis*^†^	··	··	··	··	··	··	0·95
Male	116/953	144/1148	11·14% (1·30)	12·41% (1·22)	0·90 (0·72–1·12)	0·32	··
Female	422/1803	425/1697	22·71% (1·22)	25·09% (1·14)	0·91 (0·75–1·09)	0·28	··
*C trachomatis* ^‡^	··	··	··	··	··	··	0·98
Male	78/953	101/1176	7·44% (1·34)	7·80% (1·61)	0·95 (0·68–1·34)	0·77	··
Female	280/1803	281/1746	15·45% (1·13)	16·10% (1·10)	0·96 (0·85–1·09)	0·51	··
*N gonorrhoeae* ^‡^	··	··	··	··	··	··	0·63
Male	17/953	28/1143	1·39% (1·79)	2·30% (1·66)	0·60 (0·33–1·10)	0·092	··
Female	35/1803	57/1686	1·61% (2·06)	3·21% (1·53)	0·50 (0·27–0·95)	0·036	··
*T vaginalis*t	··	··	··	··	··	··	0·055
Male	34/953	23/1176	3·83% (1·38)	1·92% (2·10)	2·00 (1·08–3·71)	0·031	··
Female	179/1803	166/1746	8·82% (1·49)	9·10% (1·46)	0·97 (0·63–1·50)	0·90	··
*C trachomatis* and *N gonorrhoeae^§^*	··	··	··	··	··	··	0·48
Male	89/953	123/1145	8·31% (1·38)	10·34% (1·37)	0·80 (0·59–1·09)	0·14	··
Female	304/1803	316/1694	16·72% (1·15)	18·70% (1·14)	0·89 (0·77–1·04)	0·14	··

*C trachomatis=Chlamydia trachomatis*. GSD=geometric standard deviation. *N gonorrhoeae=Neisseria gonorrhoeae*. *T vaginalis=Trichomonas vaginalis*.

* Adjusted for sex and age in years.

† Population-level prevalence of any (one or more) of *C trachomatis, N gonorrhoeae*, and *T vaginalis*.

‡ Population-level prevalence of single infection.

§ Population-level prevalence of one or both of *C trachomatis* and *N gonorrhoeae*.

## Data Availability

Individual, anonymised participant data and a data dictionary will be available through the London School of Hygiene & Tropical Medicine repository (Data Compass) at the time of publication, conditional upon approval for analyses from the Medical Research Council of Zimbabwe.

## Research in context

### Evidence before this study

We searched MEDLINE on Sept 25, 2023, for systematic reviews and trials using (variations and synonyms of) the search terms “sexually transmitted infections” AND “testing” OR “screening” AND “intervention” OR “trial”, with no date or language restrictions. We also searched clinical trial databases (ClinicalTrials.gov, the ISRCTN registry, and the WHO International Clinical Trials Registry Platform) for ongoing sexually transmitted infection (STI) screening trials. We found seven reviews of studies investigating the effectiveness of screening for STIs delivered in various settings (eg, at home, in antenatal care, in primary care clinics, and at school).

These reviews and our search identified a total of 13 trials. Except for five trials, all screened for chlamydia only. The main outcomes were either *Chlamydia trachomatis* prevalence (or STI prevalence in the four trials that screened for other STIs) or incidence of pelvic inflammatory disease or adverse birth outcomes. All but one trial were conducted in high-income countries. Screening for *C trachomatis* had little effect on incidence of pelvic inflammatory disease or on *C trachomatis* prevalence. Overall, these studies had low coverage and low *C trachomatis* prevalence.

Two trials screening for multiple STIs that focused on high-risk populations (>11% *C trachomatis* prevalence at baseline), in female sex workers in Peru and high-school students in the USA, found a reduction in STI prevalence in Peru and *C trachomatis* prevalence in the USA. However, one cluster-randomised trial in remote Aboriginal communities in Australia reported that, despite an increase in testing coverage for *C trachomatis, Neisseria gonorrhoeae*, and *Trichomonas vaginalis* following the implementation of continuous improvement strategies, this did not lead to a reduction in STI prevalence.

An additional four trials investigating the impact of STI screening in pregnant women on adverse birth outcomes in Botswana, China, Papua New Guinea, and South Africa were recently completed or are ongoing and yet to be published. A fifth trial among young women in France is investigating the impact of *C trachomatis* screening on incidence of pelvic inflammatory disease. No trial has investigated the effect of STI screening (outside antenatal care settings) in southern Africa, where STI and HIV prevalence are both very high.

### Added value of this study

To our knowledge, this is the first trial to evaluate the effect of an STI screening intervention (*C trachomatis, N gonorrhoeae*, and *T vaginalis*) on population STI prevalence among young people in a setting of high HIV and high STI prevalence. Although the trial found no effect of screening young people on the primary outcome—population-level prevalence of any (one or more) of *C trachomatis, N gonorrhoeae*, and *T vaginalis*—or on *C trachomatis* or *T vaginalis* prevalence, the population-level prevalence of *N gonorrhoeae* in the intervention arm was significantly lower than that in the control arm.

### Implications of all the available evidence

The absence of effect of screening on the composite of *C trachomatis, N gonorrhoeae*, and *T vaginalis* prevalence in our study might be due to the low coverage of STI screening and treatment in the intervention arm, which was unable to reduce the very high *C trachomatis* prevalence and the consequent high risk of re-infection in the community. *N gonorrhoeae* had a population-level prevalence much lower than that of *C trachomatis*, which might explain the reduction in *N gonorrhoeae* prevalence in the intervention arm. Moreover, infections caused by *N gonorrhoeae* are more likely to be symptomatic and lead to health-care attendance and prompt treatment than those caused by *C trachomatis*. The finding of lower *N gonorrhoeae* prevalence in the intervention arm is of note because *N gonorrhoeae* infection is associated with various short-term and long-term sequelae, including pelvic inflammatory disease and compromised fertility, and *N gonorrhoeae* is a WHO priority pathogen for antimicrobial resistance.

Despite findings of lower STI prevalence following targeted screening in the STICH trial and other studies, these have been mixed and the benefits and harms of STI screening need to be carefully weighed and further investigated before screening can be recommended. Harms might include increased consumption of antimicrobials within the population and subsequent onward risks of antimicrobial resistance, as well as psychosocial harms associated with STI diagnosis. In settings with high STI prevalence, screening might not affect transmission, but could have other benefits such as improved reproductive health and birth outcomes and decreased HIV transmission. The STICH trial serves as proof-of-concept of the feasibility and acceptability of population-based STI screening among youth, which will inform research and practice in other southern African countries.
