# Supplementary material for: Effect of a community-based intervention for sexually transmitted infections on population-level prevalence among youth in Zimbabwe (STICH): a cluster-randomised trial
Source: Lancet Glob Health. Author manuscript; Available in PMC 2025 Jul 20. (PMC7617908; doi:10.1016/S2214-109X(24)00373-5)
Supplement: Supplementary Materials [file EMS206793-supplement-Supplementary_Materials.zip › 1-s2.0-S2214109X24003735-mmc2.pdf]

THE LANCET
Global Health

Supplementary appendix 2

This Equitable Partnership Declaration (EPD) was submitted by the authors, and we reproduce it as supplied. It has not been peer reviewed. *The Lancet*'s editorial processes have not been applied to the EPD.

Supplement to: Dziva Chikwari C, Dauya E, Simms V, et al. Effect of a community-based intervention for sexually transmitted infections on population-level prevalence among youth in Zimbabwe (STICH): a cluster-randomised trial. *Lancet Glob Health* 2024; published online Nov 14. https://doi.org/10.1016/S2214-109X(24)00373-5.

Equitable Partnership Declaration questions

This Equitable Partnership Declaration is a statement being published online alongside papers at *The Lancet Global Health*, as a separate appendix, to allow researchers to describe how their work engages with researchers, communities, and environments in the countries of study. This is part of our broader goal to decolonise global health, handing control and leadership of research to academics and clinicians who are based in the regions of study, and to affected communities.

Please answer all questions with as much detail as possible, noting that all included information will be published open-access and it will be freely available online to all who wish to read it. If a question does not apply to your study, please state "Not applicable".

The format of and questions in this statement are currently in a pilot phase. Please email Dr Liam Messin (Liam.Messin@lancet.com; deputy editor) and Dr Kate McIntosh (Kate.McIntosh@lancet.com; senior editor) with any feedback, particularly if you find any questions unclear.

Researcher considerations

1. Please detail the involvement that researchers who are based in the region(s) of study had during a) study design; b) clinical study processes, such as processing blood samples, prescribing medication, or patient recruitment; c) data interpretation; and d) manuscript preparation, commenting on all aspects. If they were not involved in any of these aspects, please explain why.

This question is intended for international partnerships; if all your authors are based in the area of study, this question is not applicable.

This should include a thorough description of their leadership role(s) in the study. Are local researchers named in the author list or the acknowledgements, or are they not mentioned at all (and, if not, why)? Please also describe the involvement of early career researchers based in the location of the study. Some of this information might be repeated from the Contributors section in the manuscript. Note: we adhere to ICMJE authorship criteria when deciding who should be named on a paper.

**a) Study design:**<br>The first author of this manuscript and the principal investigator for this trial (CDC) is a Zimbabwean early career researcher who is also based in Zimbabwe. She was part of the team who developed the study proposal and co-led the development of the study methods with support from the original principal investigator (SF) and other co-investigators also based in Zimbabwe (KK, RAF, VS, CM, MT, CMY). Among the 19 co-authors, 13 are based in the region of study. With support from local research staff including representatives from the Ministry of Health and Child Care in Zimbabwe (AM, OM) and research assistants (who are also co-authors, PM, TM)) the principal investigator developed the manual of operations for the trial as well as the data collection tools.
**b) Clinical study processes:**<br>Researchers based in Zimbabwe were responsible for the daily clinical study processes including participant recruitment and are named as authors (CDC, ED, PM, TM, NT). Specifically, researchers involved in the study coordination, sample processing in the laboratory and oversight of participant are named. Laboratory tests processing and data was managed by TM who is a Zimbabwean early

career researcher, and she received support from a Zimbabwean laboratory technician who was employed as a research assistant (PM).
**c) Data interpretation:**<br>The data management and interpretation for this trial was led by researchers who were based in Zimbabwe for the duration of the trial (VS and TB). They played a key role in the cleaning, analysis and interpretation of the data with overall leadership from the principal investigator (CDC).
**d) Manuscript preparation:**<br>The first author of this manuscript and the principal investigator developed the first draft of this manuscript (CDC). She received support from an early career researcher based in Zimbabwe (KM) who conducted a literature review and drafted the research in context section. All authors reviewed the manuscript and supported the refinement of subsequent drafts.

2. Were the data used in your study collected by authors named on the paper, or have they been extracted from a source such as a national survey? ie, is this a secondary analysis of data that were not collected by the authors of this paper. If the authors of this paper were not involved in data collection, how were data interpreted with sufficient contextual knowledge?

   The Lancet Global Health *believe contextual understanding is crucial for informed data analysis and interpretation.*

The authors named on the paper collected the study data.

3. How was funding used to remunerate and enhance the skills of researchers and institutions based in the area(s) of study? And how was funding used to improve research infrastructure in the area of study?

   Potentially effective investments into long-term skills and opportunities within institutions could include training or mentorship in analytical techniques and manuscript writing, opportunities to lead all or specific aspects of the study, financial remuneration rather than requiring volunteers, and other professional development and educational opportunities.

   Improvements to research infrastructure could be funding of extended trial designs (such as platform trials) and use of master protocols to enable these designs, establishment of long-term contracts for research staff, building research facilities, and local control of funding allocation.

**Skills:**<br>All local research staff based in Zimbabwe who were responsible for participant recruitment and management on the study were trained on screening for sexually transmitted infections. Zimbabwean clinical staff on the study received further training on STI management including treatment protocols in Zimbabwe from the Ministry of Health and Child Care. This training was funded by the study. The Zimbabwean PI has also given further training on STI epidemiology and screening to other organisations and nurses in Zimbabwe including sitting on the Ministry of Health and Child Care STI Advisory group. This has contributed to long term skills and

opportunities for the research staff. All staff received financial renumeration while working on the study and staff wishing to further their education were provided with financial support for this during their employment on the study and were given study time. This study included MSc programmes in Development Studies and Certificates in Monitoring and Evaluation completed by Research Assistants on the study.
**Research infrastructure:**<br>Funding from the trial was used to procure two GeneXpert machines. One machine continues to be used for patients at the health facility where the study was conducted in Zimbabwe. The second machine was donated to the research institute which conducted the study in Zimbabwe, and they continue to use the machine on other studies. As part of the study procured equipment such as refrigerators and barcode scanning machines continue to be used by the research institute that conducted the study in Zimbabwe.<br><br>Additionally, a smaller pilot study was conducted in a health facility in Zimbabwe informed by the findings from and infrastructure provided by this study.

4. How did you safeguard the researchers who implemented the study?

Please describe how you guaranteed safe working conditions for study staff, including provision of appropriate personal protective equipment, protection from violence, and prevention of overworking.

The Medical Research Council of Zimbabwe (MRCZ) gave ethical approval for the study. MRCZ granted a waiver of written informed consent for attending the CHIEDZA service and approved the use of electronic consent for the endline survey. Data collectors were trained and worked in pairs. COVID-19 procedures and training were implemented to protect staff and participants including the provision of personal protective equipment. All CHIEDZA community centres had prior approval from and support from the Zimbabwe Republic Police in the event of violence at the centre. Additionally, all staff were employees of BRTI and protected by their HR policies, including their rights for annual leave and sick leave.

Benefits to the communities and regions of study

5. How does the study address the research and policy priorities of its location?

How were the local priorities determined and then used to inform the research question? Who decided which priorities to take forward? Which elements of the study address those priorities?

Adolescents and young people are a priority group for the Zimbabwe Ministry of Health and Child Care (MOHCC). The Comprehensive National HIV Communications Strategy for Zimbabwe highlighted the importance of integrated SRH/HIV services and community outreach, especially to adolescent girls and young women. The Zimbabwe National Health Strategy 2016-2020 aimed to achieve the HIV 90-90-90 targets by optimising prevention activities including HIV testing and counselling, STI control, and condom promotion. The head of the AIDS and TB Unit at MoHCC is a named trial co-investigator. A youth advisory board was formed to advise on study design.

6. How will research products be shared in the community of study?

   For instance, will you be providing written or oral layperson summaries for non-academic information sharing? Will study data be made available to institutions in the region(s) of study? The Lancet Global Health *encourages authors to translate the summary (abstract) into relevant languages after paper editing; do you intend to translate your summary?*

   We intend on translating the summary of our manuscript. We have conducted dissemination meetings with researchers in Zimbabwe as well as young people and the communities where research was conducted. We intend to make our manuscript available open access so that researchers at institutions in Zimbabwe and in Africa more widely are able to access them without any financial barriers.

   The manual of operations and any resources developed from the study are available open access on the study website.

7. How were individuals, communities, and environments protected from harm?

a) How did you ensure that sensitive patient data was handled safely and respectfully? Was there any potential for stigma or discrimination against participants arising from any of the procedures or outcomes of the study?

The study ensured that all participant data is kept private and confidential. Within the trial no participant names were collected/used but rather study IDs and biometric data which was securely stored. No test results were shared via telephone to protect participants and staff were trained on sensitive conduct including ensuring non-judgemental conduct and the protection of data. This was critical due to the sensitive nature of the trial.

b) Might any of the tests be experienced as invasive or culturally insensitive?

No.

c) How did you determine that work was sensitive to traditions, restrictions, and considerations of all cultural and religious groups in the study population?

Extensive formative work was conducted prior to the start of the study to ensure that the work was contextually appropriate and acceptable.

d) Were biowaste and radioactive waste disposed of in accordance with local laws?

Yes, the research was conducted in partnership with the Ministry of Health and Child Care in Zimbabwe and all waste was disposed of in accordance with their regulations.

e) Were any structures built that would have impacted members of the community or the environment (such as handwashing facilities in a public space)? If so, how did you ensure that you had appropriate community buy-in?

The study primarily used preexisting community venues, however, in one site a structure was conducted to ensure protection from the rain for the study team. This structure was constructed in partnership with and on the site of a community-based organisation working with the study team. The site was built with a long-term perspective and in accordance to the needs and preferences of the community based organisation.

f) How might the study have impacted existing health-care resources (such as staff workloads, use of equipment that is typically employed elsewhere, or reallocation of public funds)?

All commodities used on the study were procured by the study funding with the exception of antiretroviral therapy (ART) for HIV treatment. ART was provided by the existing health facilities as the clients were to be referred back to the health facility at the end of trial and ART is lifelong. Overall, the study may have lowered the load on existing resources during the intervention but may have increased this load at the end of the study.

8. Finally, please provide the title (eg, Dr/Prof, Mr/Mrs/Ms/Mx), name, and email address of an author who can be contacted about this statement. This can be the corresponding author.

**Name:** Dr Chido Dziva Chikwari<br>**Email: chido.dzivachikwari@lshtm.ac.uk**
