# Supplementary material for: Effect of a community-based intervention for sexually transmitted infections on population-level prevalence among youth in Zimbabwe (STICH): a cluster-randomised trial
Source: Lancet Glob Health. Author manuscript; Available in PMC 2025 Jul 20. (PMC7617908; doi:10.1016/S2214-109X(24)00373-5)
Supplement: Supplementary Materials [file EMS206793-supplement-Supplementary_Materials.zip › 1-s2.0-S2214109X24003735-mmc1.pdf]

THE LANCET
Global Health

Supplementary appendix 1

This appendix formed part of the original submission and has been peer reviewed. We post it as supplied by the authors.

Supplement to: Dziva Chikwari C, Dauya E, Simms V, et al. Effect of a community-based intervention for sexually transmitted infections on population-level prevalence among youth in Zimbabwe (STICH): a cluster-randomised trial. *Lancet Glob Health* 2024; published online Nov 14. https://doi.org/10.1016/S2214-109X(24)00373-5.

Appendix Document

1. Table of Contents

2. *STICH Eligibility Criteria* ... 4

1.1 Eligibility for the intervention ... 4

1.1.1 Inclusion criteria ... 4

1.1.2 Exclusion criteria ... 4

1.2 Eligibility for the survey ... 4

1.2.1 Inclusion criteria ... 5

1.2.2 Exclusion criteria ... 5

3. Supplementary Table 1 ... 6

4. CHIEDZA and STICH Protocol ... 7

- ***Table of Contents ... i***
- ***List of Tables and Figures ... iii***
- ***... iii***
- ***List of Abbreviations and Acronyms ... iv***
- ***Study Team ... vi***
- ***Declaration ... viii***
- ***Executive Summary ... ix***
- ***Overview of Study Design ... 1***
- ***1. Introduction ... 1***

a. 1.1 Background ... 2

i. 1.1.1 HIV Epidemic in Sub-Saharan Africa ... 2

ii. 1.1.2 HIV care cascade in young people ... 3

iii. 1.1.3 Community-based approaches to HIV service delivery ... 3

a. 1.2 Innovation ... 5

- ***2. Study Objectives and Design ... 6***

b. 2.1 Primary Objective ... 6

c. 2.2 Secondary Objectives ... 6

d. 2.3 Study design ... 6

- ***3. Study Intervention ... 9***

e. 3.1 Standard of care ... 9

f. 3.2 Intervention components ... 9

i. 3.2.1 HIV Testing and Counselling ... 10

ii. 3.2.2 Linkage to HIV care and treatment ... 10

iii. 3.2.3 Adherence support ... 11

iv. 3.2.4 SRH and HIV prevention ... 11

v. **3.2.5 General health information and counselling** 12

g. ***3.3 Delivery of Intervention*** **12**

i. **3.3.1 Configuration of Services** 12

ii. **3.3.2 Collaborations and partnerships** 14

- ***4. Study Population*** ***14***

h. ***4.1 Selection of Study Clusters*** **14**

i. ***4.2 Randomisation*** **15**

j. ***4.3 Community Engagement*** **16**

- ***5. Research Procedures and Activities*** ***19***

k. ***5.1 Prevalence survey*** **19**

l. ***5.2 Process Evaluation*** **20**

i. **5.2.1 Implementation** 21

ii. **5.2.2 Mechanisms of Impact** 21

iii. **5.2.3 Context** 21

iv. **5.2.4 Data collection methods** 22

m. ***5.3 Cost-effectiveness studies*** **24**

i. **5.3.1 Introduction** 24

ii. **5.3.2 Aims and Objectives** 25

iii. **5.3.3 Primary costing studies** 25

iv. **5.3.4. Analytical plan** 26

n. ***5.4 MHM Intervention Acceptability, Uptake, and Effectiveness (MAE) Sub-Study*** **27**

i. **5.4.1 Background and Rationale** 27

ii. **5.4.2 Study Aim and Objectives** 28

iii. **5.4.3 Theory of change** 29

iv. **5.4.4 MHM Intervention** 29

v. **5.4.5 Study Outcomes** 31

vi. **5.4.6 Data from the CHIEDZA intervention** 32

vii. **5.4.7 The Nested MHM Cohort Sub-Study Intervention** 33

o. ***5.5 STI Screening Acceptability and Effectiveness*** **36**

i. **5.5.1 Background and Rationale** 36

ii. **5.5.2 Study Aim and Objectives** 37

iii. **5.5.3 Sub-study methods** 37

iv. **5.5.4 Outcomes** 39

p. ***5.6 Pilot study of investigation into prevalence of exposure to trauma*** **39**

i. **5.6.1 Background and description** 39

- ***6. Data management*** ***41***

q. ***6.1 Process data for intervention delivery*** **41**

i. **6.1.1 Identification of service users** 41

ii. **6.1.2 Monitoring service use** 41

r. ***6.2 Outcome data from cross-sectional survey*** **42**

- ***7. Statistical considerations and Data analysis*** ***43***

s. ***7.1 Outcomes*** **43**

t. ***7.2 Sample size calculations*** **43**

u. ***7.3 Statistical analysis*** **45**

- *8. Ethical Considerations* *46*

8.1 Collaborative partnerships 46

v. *8.2 Social value* 46

w. *8.3 Scientific validity* 46

x. *8.4 Risk-benefit assessment* 47

i. **8.4.1 Benefits and risks at community level** 47

ii. **8.4.2 Risks and benefits at individual level** 48

8.5 Consent procedures 49

iii. **8.5.1 Approval from Authorities** 49

iv. **8.5.2 Community Consent** 50

v. **8.5.3 Individual Consent** 50

vi. **8.5.4 Consent to Access routine healthcare surveillance data** 54

y. *8.6 Confidentiality* 55

z. *8.7 Data and Safety monitoring* 57

aa. *8.8 Clinical management during and post-study* 57

bb. *8.9 Ethical Review* 58

- *9. Laboratory Procedures* *59*

cc. *9.1 Cross-sectional survey tests* 59

dd. *9.2 Safety issues* 59

- *10. Administrative Procedures* *61*

ee. *10.1 Regulatory approvals* 61

ff. *10.2 Trial registration* 61

gg. *10.3 Indemnity* 61

hh. *10.4 Study monitoring* 61

ii. *10.5 Protocol compliance* 61

jj. *10.6 Maintenance of records* 61

kk. 62

ll. *10.7 Use of data and publications* 62

- *11. References* *63*

2. STICH Eligibility Criteria

1.1 Eligibility for the intervention

1.1.1 Inclusion criteria

- Lives within one of the STICH intervention clusters
- Aged 16-24 years
- Female (for Trichomonas test only)
- Attends CHIEDZA service between 21 September 2020 and 30 September 2021 (Harare) or 1 January 2021 and 31 December 2021 (Bulawayo), the times when the STICH intervention is operational

1.1.2 Exclusion criteria

- Has had an STI test within the previous 3 months, unless they are symptomatic and request an STI test

1.2 Eligibility for the survey

For the prevalence survey all 16 clusters were mapped using OpenStreetMap and the road networks were divided into sections of approximately 200 metres in length. Sections were each allocated an ID number, and simple random sampling was used to select 40 street sections and a backup list of 40 extra sections, in each cluster. The list of selected street sections was sent to the study coordinator. The study coordinator arranged for the survey team to survey all selected street sections. If the first list was completed before 700 participants had been enrolled, the team moved on to the backup list.

The entire survey aimed to enrol 700 participants per cluster, to meet the requirements of the CHIEDZA trial. The sample size for STICH is 300 per cluster. Therefore, data collection days of the survey were randomly allocated to STICH or non-STICH, stratified by weekday/Saturday, with 55% of days allocated to STICH. All participants recruited on STICH days were asked to provide a urine sample which will be tested for 5 STIs using Seegene (Allplex™ STI Essential Assay). Vaginal swab samples were not used in the prevalence survey. The 5 STIs are *C. trachomatis*, *N. gonorrhoeae*, *T. vaginalis*, *Ureaplasma urealyticum* and *Mycoplasma genitalium*. 55% of days were allocated to STICH because it was estimated that data collection would be slower on STICH days (due to the urine sampling) and so 55% of days are required to obtain 43% (300/700) of the participants.

Data collectors visited every dwelling in the allocated street sections and enumerated household members. If any household members were aged 18-24 years, they approached these potential participants for consent. If eligible participants were not at home the data collectors made up to 3 return visits on three different days.

Household members aged 18-24 were shown a video which explains the survey procedures and gave consent if they agreed to participate. The video includes details of STICH including the need for a urine sample and the tests to be performed. Paper consent forms were used in case of video failure, and for a small number of participants as a control group for a randomised controlled trial of consent methods.

1.2.1 Inclusion criteria

- Aged 18-24 years
- Consent to participate
- Surveyed on a day allocated to STICH sampling

1.2.2 Exclusion criteria

- Unable or willing to provide informed consent
- Unable or unwilling to provide a urine sample

3. Supplementary Table 1

Supplementary Table 1: Characteristics or enrolled participants comparing youth included and not included in the final analysis

Characteristic		Analysed, n (%)	Enrolled but not analysed, n (%)	Chi squared, p
Age (yrs)	18-20	2885 (51.5)	402 (52.9)	χ^2=0.5, p=0.47
	21-24	2716 (48.5)	358 (47.1)	
Sex	Male	2101 (37.5)	312 (41.1)	χ^2=3.6, p=0.059
	Female	3500 (62.5)	448 (59.0)	
Education level attained	Did not complete primary	106 (1.9)	19 (2.5)	χ^2=17.8, p=0.001
	Completed primary	955 (17.1)	91 (12.0)	
	Completed Form 4	3422 (61.1)	467 (61.5)	
	Completed Form 6	698 (12.5)	119 (15.7)	
	Post-secondary	420 (7.5)	64 (8.4)	
Sexual debut	Ever had penetrative intercourse	3816 (68.5)	433 (58.0)	χ^2=32.5, p<0.001
	Never had penetrative intercourse	1757 (31.5)	313 (42.0)	
Household monthly income	<USD 50	934 (19.2)	135 (20.6)	χ^2=1.8, p=0.9
	USD 50-100	1481 (30.5)	184 (28.1)	
	USD 101-200	1456 (30.0)	200 (30.6)	
	USD 201-500	814 (16.8)	111 (17.0)	
	>USD 500	170 (3.5)	24 (3.6)	
	Missing	746	106	
Main activity	Education	1526 (27.3)	246 (32.4)	χ^2=11.5, p=0.009
	Formally employed	245 (4.4)	40 (5.3)	
	Informally employed	956 (17.1)	125 (16.5)	
	None of the above	2874 (51.3)	349 (45.9)	
Marital status	Married or living together	1084 (19.4)	87 (11.5)	χ^2=33.0, p<0.001
	Never married	4280 (76.4)	651 (85.7)	
	Divorced, widowed or separated	237 (4.2)	22 (2.9)	

4. CHIEDZA and STICH Protocol

Community based interventions to improve HIV outcomes in youth: a cluster randomised trial in Zimbabwe (CHIEDZA)

Chiedza
Ukukhanya
Health For Our Future

Sponsored by:
London School of Hygiene and Tropical Medicine (LSHTM)

Funded by:
The Wellcome Trust
Grant No: 206316/Z/17/Z
United Kingdom

Principal Investigator
Rashida Ferrand
London School of Hygiene and Tropical Medicine
London, United Kingdom

Version 4.0
April 30th 2021

• Table of Contents

List of Tables and Figures iii
List of Abbreviations and Acronyms iv
Study Team vi
Declaration viii
Executive Summary ix
Overview of Study Design 1
1. Introduction 1
1.1 Background 2
1.1.1 HIV Epidemic in Sub-Saharan Africa 2
1.1.2 HIV care cascade in young people 3
1.1.3 Community-based approaches to HIV service delivery 3
1.2 Innovation 5
2. Study Objectives and Design 6
2.1 Primary Objective 6
2.2 Secondary Objectives 6
2.3 Study design 6
3. Study Intervention 9
3.1 Standard of care 9
3.2 Intervention components 9
3.2.1 HIV Testing and Counselling 10
3.2.2 Linkage to HIV care and treatment 10
3.2.3 Adherence support 11
3.2.4 SRH and HIV prevention 11
3.2.5 General health information and counselling 12
3.3 Delivery of Intervention 12
3.3.1 Configuration of Services 12
3.3.2 Collaborations and partnerships 14
4. Study Population 14
4.1 Selection of Study Clusters 14
4.2 Randomisation 15
4.3 Community Engagement 16
5. Research Procedures and Activities 19
5.1 Prevalence survey 19
5.2 Process Evaluation 20
5.2.1 Implementation 21
5.2.2 Mechanisms of Impact 21
5.2.3 Context 21
5.2.4 Data collection methods 22
5.3 Cost-effectiveness studies 24
5.3.1 Introduction 24
5.3.2 Aims and Objectives 25
5.3.3 Primary costing studies 25
5.3.4. Analytical plan 26
5.4 MHM Intervention Acceptability, Uptake, and Effectiveness (MAE) Sub-Study 27
5.4.1 Background and Rationale 27

5.4.2 Study Aim and Objectives....28
5.4.3 Theory of change....29
5.4.4 MHM Intervention....29
5.4.5 Study Outcomes....31
5.4.6 Data from the CHIEDZA intervention....32
5.4.7 The Nested MHM Cohort Sub-Study Intervention....33
5.5 STI Screening Acceptability and Effectiveness....36
5.5.1 Background and Rationale....36
5.5.2 Study Aim and Objectives....37
5.5.3 Sub-study methods....37
5.5.4 Outcomes....39
5.6 Pilot study of investigation into prevalence of exposure to trauma....39
5.6.1 Background and description....39
6. Data management.... 41
6.1 Process data for intervention delivery....41
6.1.1 Identification of service users....41
6.1.2 Monitoring service use....41
6.2 Outcome data from cross-sectional survey....42
7. Statistical considerations and Data analysis.... 43
7.1 Outcomes....43
7.2 Sample size calculations....43
7.3 Statistical analysis....45
8. Ethical Considerations.... 46
8.1 Collaborative partnerships....46
8.2 Social value....46
8.3 Scientific validity....46
8.4 Risk-benefit assessment....47
8.4.1 Benefits and risks at community level....47
8.4.2 Risks and benefits at individual level....48
8.5 Consent procedures....49
8.5.1 Approval from Authorities....49
8.5.2 Community Consent....50
8.5.3 Individual Consent....50
8.5.4 Consent to Access routine healthcare surveillance data....54
8.6 Confidentiality....55
8.7 Data and Safety monitoring....57
8.8 Clinical management during and post-study....57
8.9 Ethical Review....58
9. Laboratory Procedures.... 59
9.1 Cross-sectional survey tests....59
9.2 Safety issues....59
10. Administrative Procedures.... 61
10.1 Regulatory approvals....61
10.2 Trial registration....61
10.3 Indemnity....61
10.4 Study monitoring....61
10.5 Protocol compliance....61
10.6 Maintenance of records....61
10.7 Use of data and publications....62
11. References.... 63

- # List of Tables and Figures

Table 1: Rationale for choice of intervention components 9
Table 2: Configuration of the intervention to provide Youth-Friendly Services 13
Table 3: Process Evaluation Summary 23
Table 4: Overview of costing approach 26
Table 5: MHM Intervention 31
Table 6: Sample size calculation for primary outcome 44
Table 7: Sample size calculations for secondary outcome: Awareness of HIV positive status 44
Table 8: Sample size calculations for STI outcomes 45
Table 9: Types of data to be collected and consent 54

-

Figure 1: The cascade of HIV care 2
Figure 2: Timeline of Study 8
Figure 3: HIV prevalence among 15-64 year olds by province (ZIMPHIA estimates) 15
Figure 4: VL suppression (VLS) among 15-64 PHIV year olds by province (ZIMPHIA estimates) 16
Figure 5: Theory of Change model for the MAE sub-study 30
Figure 6: Different forms of data and linkage to fingerprints 42

- ## List of Abbreviations and Acronyms

AIDS	Acquired Immunodeficiency Syndrome
ANC	Antenatal Care
ANRS	National Agency for Research on AIDS and Hepatitis
ART	Anti-Retroviral Therapy
BRTI	Biomedical Research and Training Institute
BV	Bacterial Vaginosis
CAG	Community Adherence Group
CaPS	Chiedza Peer Support
CARG	Community ART Refill Group
CBO	Community-Based Organisation
CHW	Community Health Worker
CMD	Common Mental Disorder
CrAg	Cryptococcal Antigen
CT	Chlamydia *trachomatis*
DALY	Disability Adjusted Life Year
DSMB	Data Safety Monitoring Board
FGD	Focus Group Discussion
GC	Gonococcus (Neisseria *gonorrhoea*)
GCLP	Good Clinical Laboratory Practice
GCP	Good Clinical Practice
GDPR	General Data Protection Regulation
HCW	Health Care Worker
HIV	Human Immunodeficiency Virus
HSV-2	Herpes Simplex Virus 2
HTC	HIV Testing and Counselling
ICER	Incremental Cost-Effectiveness Ratio
IDI	In-Depth Interview
IEC	Information, Education and Counselling
IPV	Intimate Partner Violence
IRB	Institutional Review Board
LARC	Long Acting Reversible Contraception
LEC	Life events Checklist
LMIC	Low and Middle Income Countries
LSHTM	London School of Hygiene and Tropical Medicine
mHealth	Mobile Health
MHM	Menstrual Hygiene Management
MoHCC	(Zimbabwe) Ministry of Health and Child Care
MRCZ	Medical Research Council of Zimbabwe
NGO	Non-Governmental Organisation
OI	Opportunistic Infection
OMT	Oral Mucosal Test

PITC	Provider-initiated HIV testing and counselling
PLHIV	People Living With HIV
POI	Point of Interest
PrEP	Pre-Exposure Prophylaxis
PTSD	Post-traumatic stress disorder
RA	Research assistant
RCT	Randomised Controlled Trial
RCZ	Research Council of Zimbabwe
RDT	Rapid Diagnostic Test
RGIO	Research Governance and Integrity
RTI	Reproductive Tract Infection
SMS	Short Messaging Service
SRH	Sexual and Reproductive Health
SSA	Sub-Saharan Africa
STI	Sexually Transmitted Infection
TB	Tuberculosis
TSC	Trial Steering Committee
TV	Trichomonas *vaginalis*
UK	United Kingdom
UNAIDS	United Nations Programme on HIV/AIDS
UNICEF	United Nations Children's Fund
UTI	Urinary Tract Infection
VL	Viral load
VMMC	Voluntary Male Medical Circumcision
WHO	World Health Organization
YAG	Youth Advisory Group
ZIMPHIA	Zimbabwe Population-based HIV Impact Assessment

- ## Study Team

Tsitsi Apollo

Co-investigator
Deputy Director HIV/AIDS and STIs
AIDS & TB Unit
Ministry of Health and Child Care
5th Floor Mukwati Building
Fifth Street / Livingstone Avenue
Harare, Zimbabwe
Phone: +263 712 871980 / +263 8644 211024
Email: tsitsiapollo2@gmail.com

Tsitsi Bandason

Data Manager
Biomedical Research and Training Institute
10 Seagrave Road
Avondale
Harare, Zimbabwe
Phone: +263 772 942268
Email: tbandason@brti.co.zw

Sarah Bernays

Senior social scientist
School of Public Health
University of Sydney
Camperdown
2038 NSW, Australia
Phone: +61 2 9036 7137
Email: sarah.bernays@sydney.edu.au

Chido Dziva Chikwari

Research Fellow
Biomedical Research and Training Institute
10 Seagrave Road
Avondale
Harare, Zimbabwe
Phone: +263 772 773879
Email: chido.dziva.chikwari@lshtm.ac.uk

Ethel Dauya

Study coordinator
Biomedical Research and Training Institute
10 Seagrave Road
Avondale
Harare, Zimbabwe
Phone: +263 774 452172
Email: edauya@brti.co.zw

Richard Hayes

Statistical Epidemiologist
MRC Tropical Epidemiology Group
Department of Infectious Disease Epidemiology
London School of Hygiene & Tropical Medicine
Keppel Street
London WC1E 7HT, UK
Phone: +44 207 927 2243
Email: richard.hayes@lshtm.ac.uk

Dr Pitchaya Indravudh

Health Economist
London School of Hygiene & Tropical Medicine
15-17 Tavistock Place
London WC1H 9SH, UK
Phone: +44 207 927 2577
Email: Pitchaya.indravudh@lshtm.ac.uk

Katharina Kranzer

Epidemiologist (Implementation Science)
Clinical Research Department
London School of Hygiene & Tropical Medicine
Keppel Street
London WC1E 7HT, UK
Phone: +44 207 927 2577
Email: katharina.kranzer@lshtm.ac.uk

Constance Mackworth-Young

Social & Implementation Scientist
London School of Hygiene & Tropical Medicine
Keppel Street
London WC1E 7HT, UK
Phone: +44 207 927 2234
E-mail: Constance.Mackworth-Young1@lshtm.ac.uk

Bernard Madzima

Director Family health Services
Ministry of Health and Child Care
5th Floor Kaguvi building
Fifth Street / Livingstone Avenue
Harare, Zimbabwe
Phone: +263 772 481 478 / +263 4 798546-60
Email: madzimabernard@gmail.com

Aoife Doyle

Epidemiologist (M-health)
Department of Infectious Disease Epidemiology
London School of Hygiene & Tropical Medicine
Keppel Street
London WC1E 7HT, UK
Phone: +44 (0) 207 927 2041
Email: aoife.doyle@lshtm.ac.uk

Constancia Mavodza

Research Fellow
Biomedical Research and Training Institute
10 Seagrave Road
Avondale
Harare, Zimbabwe
Phone: +263 776 005501
Email: constancia.mavodza@gmail.com

Rashida Ferrand

Principal Investigator
Biomedical Research & Training Institute AND
Clinical Research Department
London School of Hygiene & Tropical Medicine
Keppel Street
London WC1E 7HT, UK
Phone: +44 207 927 2577 /+263 772 165 755
Email: rashida.ferrand@lshtm.ac.uk

Victoria Simms

Statistician
MRC Tropical Epidemiology Group
Department of Infectious Disease Epidemiology
London School of Hygiene & Tropical Medicine
Keppel Street
London WC1E 7HT, UK
Phone: +44 207 927 2234
Email: victoria.simms@lshtm.ac.uk

Suzanna Francis

Epidemiologist (MHM & STI)
MRC Tropical Epidemiology Group
Department of Infectious Disease Epidemiology
London School of Hygiene & Tropical Medicine
Keppel Street
London WC1E 7HT, UK
Phone: +44 (0)20 7927 2245
Email: suzanna.francis@lshtm.ac.uk

Mandi Tembo

Research Fellow (MHM)
Biomedical Research and Training Institute
10 Seagrave Road
Avondale
Harare, Zimbabwe
Phone: +263 772 676417
Email: tembom91@gmail.com

Chris Grundy

Geographical Information Systems
Department Infectious Disease Epidemiology
London School of Hygiene & Tropical Medicine
Keppel Street
London WC1E 7HT, UK
Phone: +44 207 927 2441
Email: chris.grundy@lshtm.ac.uk

Helen Weiss

Statistical Epidemiologist
MRC Tropical Epidemiology Group
Department of Infectious Disease Epidemiology
London School of Hygiene & Tropical Medicine
Keppel Street
London WC1E 7HT, UK
Phone: +44 207 927 2087
Email: helen.weiss@lshtm.ac.uk

• Declaration

Community based interventions to improve HIV outcomes in youth: a cluster randomised trial in Zimbabwe (CHIEDZA)

I, the Principal Investigator, agree to conduct this study in full compliance with this protocol, Good Clinical Practice and the regulatory requirements of the Medical Research Council of Zimbabwe.

I will ensure that the study team and all colleagues involved in the conduct of this study are informed about the obligations incurred by their contribution to the study.

The study team declares no conflicts of interest.

Name of Principal Investigator: Professor RASHIDA FERRAND

Signature of Principal Investigator:

Date: 10th May 2021

• Executive Summary

Young people fare disproportionately poorly across the HIV care continuum compared to other age-groups; the prevalence of undiagnosed HIV is substantially higher, and coverage of and adherence to antiretroviral therapy is lower, resulting overall in worse virological outcomes.

Aim: The aim is to determine the impact of an integrated community-based package of HIV services incorporating HIV testing, linkage to care and ongoing adherence support, combined with sexual and reproductive health services and general health counselling for 16 to 24 year olds on population level HIV viral load in a high HIV prevalence setting.

Design: This is a two-arm cluster-randomised trial in 24 clusters randomised 1:1 to standard of care or to the intervention package.

Intervention: Community-based package of services that includes: HIV testing and counselling, delivery of antiretroviral therapy, adherence support groups, mHealth, condoms, menstrual hygiene management, contraception and treatment of sexually transmitted infections, referral for voluntary medical male circumcision , risk reduction counselling and general health information and counselling. The intervention will be implemented over a 30 month period (a six month extension over the originally planned 24 months to mitigate against the effects of lockdowns due to the COVID19 pandemic. The intervention will be implemented in 12 clusters, each with a population of approximately 2500 16-24 year olds.

Study Outcomes: The study outcomes will be determined at a population level through a community cross-sectional survey among 18 to 24 year olds two years following the implementation of the intervention. The primary outcome is the proportion with HIV with a viral load <1000 copies/ml. The secondary outcomes will reflect each step of the HIV care cascade: proportion with HIV who know their HIV status, proportion of those who know their HIV-positive status who are currently taking antiretroviral therapy, proportion of those taking antiretroviral therapy who are virally suppressed. Sexual and reproductive health knowledge, and other health risks and behaviour will also be assessed. In addition a random sample of 300 per cluster will be surveyed to investigate the effect of providing unselected STI testing on prevalence of STIs.

Study population: The end-line survey will recruit 700 18-24 year olds per cluster (total 16,800).

Study sites: The study will be conducted in 3 provinces in Zimbabwe: Harare, Bulawayo and Mashonaland East. Unselected STI testing will be offered in Harare and Bulawayo only and the outcomes will be assessed in these provinces only (n=16 clusters).

Study Duration: The planned duration of the entire study will be 4 years

- ## Overview of Study Design

24 community clusters

Approx. 2,500 16-24 year olds per cluster
Approx. Total: 60,000 across all clusters

Randomisation

Control Arm: 12 clusters

Standard of Care:

- Existing HIV testing and care services
- Existing sexual and reproductive health services

Intervention Arm: 12 clusters

Community-based service over 2 years:

- HIV testing
- Immediate ART initiation
- ART delivery
- ART Adherence support
- Sexual & reproductive health
- General health information and counselling

Process Evaluation of intervention
Cost-effectiveness of intervention

Community prevalence survey at 30monthspost intervention implementation

700 8-24 year olds/ cluster
Total 16,800 across all clusters

Primary Outcome Measure

- % with HIV with HIV viral load < 1000 copies/ml (goal: 73% in the intervention arm)

Secondary Outcome Measures

- % with HIV who know that they are HIV positive (goal: 90% in intervention arm)
- % of those who know their status, who are currently on ART (goal: 90% in intervention arm)
- % of those who are currently on ART, are virally suppressed. (goal: 90% in intervention arm)

Exploratory Outcomes: health behaviours, risks and morbidities

Note: ART to be provided according to Zimbabwe national guidelines
MHM substudy not included in this chart for simplicity but described in detail in the protocol
Evaluation of effect of STI prevalence not included in this chart for simplicity but described in detail in protocol

a. 1.1 Background

i. 1.1.1 HIV Epidemic in Sub-Saharan Africa

Sub-Saharan Africa bears over two-thirds of the worldwide burden of HIV infection, with 25.5 million of the 36.7 million PLHIV worldwide living in this region.[1] The HIV epidemic in this region has had a devastating effect on morbidity, mortality and national economies, as well as wider societal effects. While recent declines in incidence have been observed in several African countries, particularly among young women, HIV prevalence remains extremely high in many parts of SSA.[2] In particular, Southern Africa remains severely affected, with extensive, generalized HIV epidemics and very high HIV prevalence in most countries.[1] Zimbabwe is among the most severely-affected countries with an estimated 1.3 million PLHIV and an adult HIV prevalence of 14.4% in 2016.[1,3]

The 2016 WHO guidelines recommend immediate initiation of ART for all PLHIV, irrespective of age or disease stage.[4] These recommendations are based on the results of trials which showed both clinical benefits (START and ANRS 12136 (Temprano)) and reduced HIV transmission following early ART initiation (HPTN052).[5-7] Thus, a substantially larger number of PLHIV are now eligible for ART, many of whom remain undiagnosed; e.g. the size of the potential treatment cohort globally has almost doubled, from 18.2 million to 36.7 million since introduction of the revised treatment guidelines.[2]

ART, if taken optimally, results in viral suppression, which in turn reduces the risk of mortality in an individual but also reduces the risk of HIV transmission to their partners, conferring a public health benefit. The prerequisite steps to achieving virological suppression are identification of undiagnosed HIV-positive individuals, linking them to care and ensuring retention in care and treatment adherence (Figure 1).

Figure 1: The cascade of HIV care

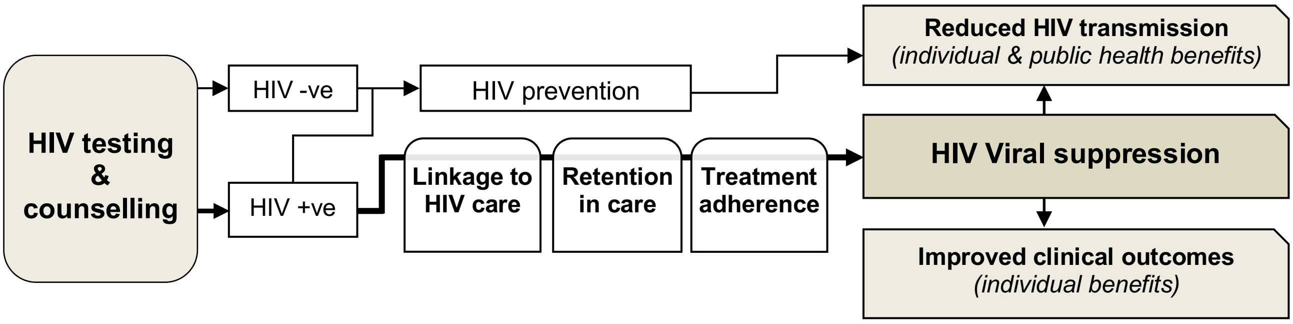

While Zimbabwe has made good progress towards increasing access to ART, there will be an increasing pool of untreated HIV-infected individuals who will need treatment in the next few years in addition to those already on treatment, given the change in WHO treatment guidelines, which the country has adopted.[8] There is also a risk that individuals initiating ART earlier in the course of infection (and therefore more likely asymptomatic) may discontinue treatment due to no perceived short-term benefits from ART; or that expanded treatment eligibility may result in overcrowding of services and therefore impact adversely on patients' engagement in care.[9,10] Furthermore, there were an estimated 40,000 new HIV infections in 2017 in Zimbabwe.[1] Unless the number of new

infections can be steeply reduced, it will be increasingly difficult and costly to provide ART for all those who need it.[11] Therefore, HIV prevention remains a pressing priority.

ii. 1.1.2 HIV care cascade in young people

Young people are a very diverse population, and several terms are used interchangeably to describe this group. For the purpose of this document, young people are considered to be persons between the age of 10 and 24 years, adolescents are persons aged between 10 and 19 years while youth are considered to be persons between the age of 16 and 24 years.[12]

Youth not only have one of the highest incidence rates of HIV of any age-group but they fare disproportionately poorly across each step of the HIV cascade compared with other age-groups: the prevalence of undiagnosed HIV infection is substantially higher; once diagnosed linkage to care is lower; once enrolled in care, they are more likely than older people to drop out, and, as with other chronic diseases, treatment adherence is poorer, leading to worse virological outcomes compared to other age groups.[13,14]

To achieve the ambitious UNAIDS 90-90-90 targets (whereby 90% of PLHIV know their HIV status, 90% of people who know their HIV-positive status are accessing treatment and 90% of people receiving treatment have suppressed VL),[15] there is now an even more pressing need to improve HTC, engagement with services and adherence; *particularly* among youth.[16] Furthermore, because of demographic shifts, there are increasingly large cohorts of youth especially in SSA, an age-group in which the risk of HIV transmission is highest. Therefore, HIV prevention among youth will need to be integral to HIV service delivery.

iii. 1.1.3 Community-based approaches to HIV service delivery

Health systems in high HIV prevalence countries in SSA are already overwhelmed by the demands of HIV care provision. It is clear that there will be major difficulties in sustaining treatment provision for a continuously expanding number of HIV-infected patients. In recent years, there has been a move towards "differentiated HIV care" models (www.differentiatedcare.org). Differentiated care is a client-centred approach that simplifies and adapts HIV services across the HIV cascade to reflect the preferences, expectations and needs of various groups of PLHIV and reduce unnecessary burdens on the health system. Differentiated HIV care requires an understanding of both the barriers and the likely facilitators to accessing services across the HIV cascade in a particular group.

Healthcare workers tend to offer HTC to those presenting with conditions indicative of HIV, which likely identifies individuals with symptomatic HIV.[17] Youth are a relatively healthy age-group and therefore health facility usage rates among this group are low; in addition there are substantial personal, social, legal and structural barriers that limit their access to key health services.[18] Restrictive laws and policies, including age of consent laws and adult-oriented HIV services that are perceived as intimidating and of poor quality, discourage service uptake. Community-based HTC strategies may be able to diagnose individuals at an earlier stage of infection by being more accessible and not relying on providers' discretion or on a client visiting a health facility.[19] Community-based HTC approaches have resulted in high uptake of testing; in the NIMH Project Accept

(HPTN 043) trial of community-based HTC, the increase in proportion of first-time testers was especially pronounced in men and adolescents, two groups with the lowest access to and coverage by conventional HIV testing services, and uptake of home-based HTC was 80% within the POPART trial.[20,21] Similarly, in a study evaluating HIV self-testing in communities in Malawi, the uptake was 78% in 16-19 year olds, the highest of any age-group.[22,23]

Identifying currently undiagnosed youth will only result in successful treatment outcomes if combined with an effective strategy to enhance linkage to and retention in care and support for maintaining adherence to treatment. In recent years, strategies that simplify and streamline the ART initiation process or decentralise HIV care to primary health facility level or even to community level have shown success in facilitating linkage to and retention in care.[23-25] Furthermore, there is evidence that lay cadres can be successfully leveraged to provide HTC and facilitate linkage to care.[26] Differentiated ART delivery aims to improve retention in care by providing flexibility in the way PLHIV access treatment. Four innovative service delivery models have emerged in response to context-specific client needs and health systems challenges met in different settings:[27]

ii. Health care worker-managed group (adherence clubs): Clients receive their ART refills in a group and either a professional or a lay health care staff member manages this group. The groups meet within and/or outside of health care facilities.

iii. Client-managed group (known as CAGs): Clients receive their ART refills in a group but this group is managed and run by clients themselves. Generally, client-managed groups meet outside of health care facilities.

iv. Facility-based individual models: ART refill visits are separated from clinical consultations. When clients have an ART refill visit, they bypass any clinical staff or adherence support and proceed directly to receive their medication.

v. Out-of-facility individual models: ART refills, and in some cases, clinical consultations are provided to individuals outside healthcare facilities including home-based delivery or community-based delivery.

The models are flexible to accommodate clients who may require referral to reflect the increased clinical needs of the client. While several studies have demonstrated the feasibility and effectiveness of these models, several questions remain. Firstly, these models have predominantly been implemented among clients who are clinically stable on ART. Secondly, most studies have not evaluated these ART delivery models among young people.[28]

Ongoing adherence support will be required if viral suppression rates are to improve. There is no one single approach that will address the multiple barriers to adherence and it is likely that a multipronged approach will be needed.[29] There is sparse evidence on effective interventions to improve adherence among youth. Community-based support, mHealth, and group counselling are strategies that have been used in adult populations and show promise for adaptation and testing among youth.[30]

a. 1.2 Innovation

The CHIEDZA intervention addresses a critical gap in the field of HIV care. There is increasing recognition that existing interventions, which are largely facility-based have not been successful in engaging youth and outcomes among this age-group across the HIV cascade have been worse than in other age-groups.[31] The proposed project speaks strongly to WHO recommendations to develop innovative community-based strategies with task sharing as more efficient and cost-effective approaches and as a way to reduce pressure on already heavily over-burdened clinics.[32] There is evidence that community-based approaches may address some of the barriers to access of services and are more acceptable. Potentially, those who stand to benefit most from such a differentiated care approach are groups such as young people who do not access routine clinic care and require more attention to achieve quality HIV care outcomes. While the concept of differentiated care is applicable across the HIV care cascade from prevention to testing to viral suppression, the majority of studies have focused on provision of ART only.[28] The CHIEDZA intervention will address the whole HIV care cascade including HTC, support for linkage to care and adherence. The integration of components is likely to have a synergistic effect because the outcome of each step of the HIV care cascade is conditional on that of the previous step. The intervention also incorporates an SRH intervention, HIV prevention and general health information and counselling. As well as being essential components of HIV service delivery and of a comprehensive response to the HIV epidemic, integration of these services will likely lead to higher acceptability and greater potential for scaleability.[2,33]

Youth were involved in the development of the intervention, including the content and mode of delivery of the intervention and user-centred design will be used to refine the intervention. Promising strategies including HIV self-testing, streamlined ART initiation, a differentiated ART delivery model, community-based support and mhealth are being incorporated, adapted for youth. For example, implementing a differentiated HIV care delivery model following diagnosis rather than once clients are stable may improve retention, particularly in youth - a group at high risk of loss-to-follow-up after diagnosis.[34] Given that HIV-positive youth are likely to have been infected recently, the majority will be clinically well and therefore unlikely to require complex clinical investigations prior to ART initiation, making streamlined ART initiation outside a facility setting feasible.[35]

This will be the first study in SSA to investigate the impact on population-level viral load of a combined approach addressing every step of the HIV cascade among youth. The trial will have important public health outcomes and will definitively determine whether the proposed strategy has a role in contributing towards meeting the ambitious global targets aimed at ending the HIV epidemic.[15,36] Importantly, such a model could be adapted to serve as a platform for delivery of services for other chronic conditions (e.g. common mental disorders, for which lay counsellor interventions have recently been shown to be highly effective in Zimbabwe and are being scaled-up nationally).[37] The study incorporates a detailed process evaluation and study of cost-effectiveness to inform scalability should it prove effective. Finally, taking advantage of the outcome survey, the project will provide much needed data at population level on health-related behaviours and risk factors for the leading causes of morbidity in youth.[38]

- ## 2. Study Objectives and Design

b. 2.1 Primary Objective

The primary objective of this study is to measure the impact of an integrated community-based package of SRH and HIV services addressing the whole HIV care cascade on HIV VL among youth (defined as those aged 16-24 years) at population level.

c. 2.2 Secondary Objectives

The secondary objectives of the study are to:

- Measure the impact of the intervention on each step of the HIV care cascade at population level: i)proportion of PLHIV aware of their status ii) proportion aware of their status taking ART and iii) proportion on ART who are virally suppressed (corresponding to the UNAIDS 90-90-90 targets[15])
- Estimate the cost and cost-effectiveness of the intervention
- Conduct a mixed-methods process evaluation of the implementation of the intervention to inform the factors required for scalability and sustainability
- Estimate the prevalence of important health-related risk factors and behaviours

d. 2.3 Study design

A cluster randomised trial design will be used to address the objectives of the study. The study will be conducted in three provinces of Zimbabwe (Harare, Bulawayo and Mashonaland East). A total of 24 study clusters, stratified by province, will be randomised in a 1:1 allocation ratio to one of the following two arms:

i. Control Arm - Existing HIV and SRH services
ii. Intervention Arm - Community-based intervention

The target population for the intervention will be individuals aged 16-24 years. In Zimbabwe, individuals aged 16 years and older are able to access SRH and HIV services without guardian consent.

The definition of a cluster, cluster selection and the randomisation method are described in Section 4.2. A pilot will be conducted to test the relevance, acceptability and feasibility of the intervention components, and to optimise trial procedures. The pilot will be delivered over a three-month period in one of the trial clusters in Harare. Iterative loops of evaluation will be used to refine the intervention procedures during the pilot phase.

The intervention will be implemented over a 30 month period in each cluster. This is an extension of six months beyond the originally planned 24 months to mitigate against the effects of the COVID19-related lockdowns during which service provision was not feasible. At the end of this period, a population cross-sectional survey will be conducted to ascertain outcomes. The deployment of the intervention will occur in phases with 4 clusters implementing the intervention first, followed by the next 4 clusters 3 months later and the final 4 clusters starting a further 3 months later. The intervention will be implemented in phases to ensure that the final survey is completed within three months of the end of the intervention.

A detailed mixed-methods process evaluation of the intervention based on the MRC Process Evaluation Framework[39] will be conducted alongside the intervention to explore three core functions of the intervention components: i) implementation, ii) mechanisms of impact and iii) context, to provide guidance for sustainable and scalable implementation of the intervention should it prove effective. Across the pilot and intervention, data collection will include observations and real-time feedback, as well as a combination of quantitative process data and focus group discussions and in-depth interviews with youth and those involved in intervention delivery. Data will be collected at multiple time points, using multiple methods and sources and triangulated with outcome data. Purposive sampling will be used to determine the sampling frame, with consideration of type of setting and provider and participant characteristics (e.g. age, gender).

An economic evaluation will be undertaken from the health provider perspective to estimate the costs and incremental cost-effectiveness of providing community-based HTC and care. The economic costing will be undertaken using UNAIDS guidelines,[40] and will estimate costs of providing the different components of the CHIEDZA intervention and for providing routine HIV testing and treatment services. Findings from the CHIEDZA trial, process evaluation and primary costing studies will be synthesised within a decision-analytic modelling framework to undertake an incremental cost-effectiveness analysis. The model will estimate the incremental cost per DALY averted through implementation of the CHIEDZA intervention.

Figure 2: Timeline of Study

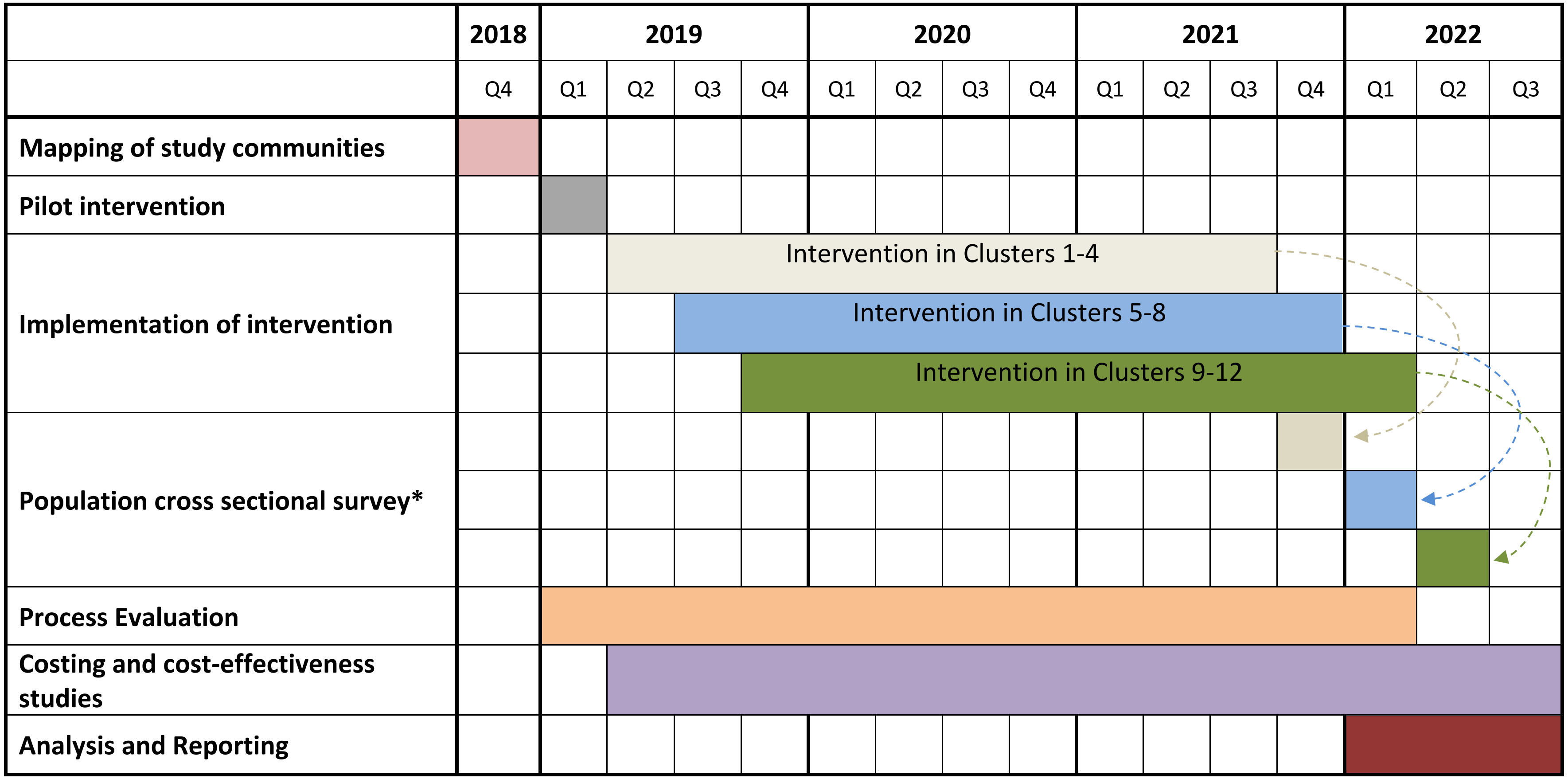

*4 intervention and 4 control clusters (total 8 clusters) surveyed per round

- ## 3. Study Intervention

e. *3.1 Standard of care*

Current standard of care in Zimbabwe consists largely of facility-based services. Existing services on HIV testing, ART delivery, VMMC, SRH and other youth services will be mapped in the control and intervention communities.

f. *3.2 Intervention components*

The intervention will be delivered in a community-based venue combined with outreach activities (details in Section 3.3.1). The rationale for the choice of the intervention components is summarised in Table 1.

Table 1: Rationale for choice of intervention components

Intervention component		Rationale
**HTC**	• Community-based HTC + HTC for sexual partners	• Anticipate high coverage (80%) among youth living with HIV with convenient services in the community, compared to much lower rates through facility-based approach alone[22]<br>• Earlier HIV diagnosis
**Linkage to care and ART initiation**	• ART initiation at community level<br>• Simplified ART initiation criteria	• ART initiation at community level kick-starts the linkage to care process[24]<br>• Simplification of ART initiation process (without multiple pre-ART initiation visits) improves linkage to and retention in care[41]<br>• Zimbabwe National Policy recommending ART regardless of age and disease stage streamlines ART initiation process[8]<br>• Zimbabwe has a standard decentralised ART delivery approach
**Retention in care & ART adherence**	• Community-based ART delivery<br>• Facilitated support groups<br>• Regular SMS	• Provision of ART in the community provides flexibility and convenience<br>• Maintaining contact in the community and community-based support encourages retention in care[25]
**SRH & HIV prevention**	• Age-appropriate risk reduction counselling<br>• Condoms; MHM and contraception counselling and products<br>• Expedited referral for VMMC<br>• Syndromic STI management + Testing	• Integration of SRH within the HIV care cascade addresses a longstanding gap in HIV programming for youth and improve retention in services[18,42]<br>• Risk reduction counselling insufficient without availability of commodities[43]<br>• Provision of MHM and other SRH products enhances acceptability by community and youth and enhances social marketing[44]<br>• Community-based HTC linked with active support increases VMMC uptake[45]

**General Health counselling**	• Risk-reduction counselling<br>• Provision of information<br>• Referral to specific services	• Improves acceptability and uptake if the intervention is perceived to not have exclusive focus on SRH and HIV<br>• A more holistic approach as the intervention addresses issues of importance to AYP

i. 3.2.1 HIV Testing and Counselling

Clients will be offered a choice of HIV testing performed by a HCW or to self-test privately for HIV on-site using an OMT , at the centre. The team will provide a demonstration on the use of the OMT and OMT kits will have detailed instructions on using the kit . . All positive HIV test results will require a second confirmatory test with a blood-based RDT. Discordant or inconclusive results will be further evaluated according to national guidelines. The need for retesting will be determined on an individual basis. In general, clients will be advised to retest on an annual basis. HTC for partners will be provided through asking CHIEDZA attendees to bring their partners to the service.

ii. 3.2.2 Linkage to HIV care and treatment

Those who test HIV-positive will be registered for HIV care by the CHIEDZA intervention team and given a National HIV programme number (as well as a CHIEDZA ID number if they choose to access care through the CHIEDZA intervention). The individual will be considered registered at the nearest clinic but will not need to attend the clinic in person to register. The HIV records of the individual (the nationally used MOHCC OI/ART Record or "green book") will be maintained at the nearest clinic and updated by the study team when drugs are collected. This will ensure that the individual remains part of the national HIV programme. Locator information will be collected so that the study team is able to follow-up the individuals should they not attend to collect their ART medication. Those who prefer to register and receive their ART directly at the health facility will be able to do so and a member of the study team will offer to accompany them to the nearest health facility and help them to register for HIV care.

Immediate initiation of ART, irrespective of CD4 cell count or disease stage, will be offered, if clients are assessed to be ready to start and have no clinical contraindications to starting immediately (e.g. an OI). If the CD4 is less than 100/µl a CrAg test to exclude cryptococcal disease will be performed before ART initiation, to all those who test HIV-positive, as well as those who were diagnosed previously but have not yet initiated ART either because they had not been eligible under the previous guidelines or because they had not registered for care at / been lost to-follow from a health facility. Those who claim to have been diagnosed with HIV previously and have not been registered in care will be required to have a confirmatory HIV test. TB screening and antibiotic prophylaxis against opportunistic infections will be given according to national guidelines. Barring contraindications, the standard first line regimen recommended by the national guidelines will be used. The small number of individuals for whom this regimen is contraindicated according to local guidelines will be treated using alternative regimens as recommended by local guidelines for ART patients. There

will be clearly defined pathways to referral to a health facility should there be any clinical indications (e.g. toxicity, incident symptoms, suspected treatment failure). Any individual initiating ART through the CHIEDZA service will continue to receive ART as any other clinic client would. They will not be taken off ART if the study ends early or at the natural end of the study.

iii. 3.2.3 Adherence support

HIV-positive clients will be offered recruitment into support groups (CAPS groups) following HIV diagnosis. The CAPS group will meet every month and will be modelled on the existing "CARGs" (based on the CAG model) that have already been implemented in Zimbabwe. CARGs comprise a group of HIV-positive individuals who meet on a regular basis with one member of the group responsible for collection of ART for other members of the group. CAPS groups will consist of 8-10 HIV-positive youth who will meet monthly for semi-structured discussion about issues relevant to youth, social activities and peer support. The facilitator will be responsible for collecting the drugs for each member of the CAPS group from the local health facility. The plan will be for the CAPS to eventually be self-sustaining and run themselves- but during the intervention period, each CAPS group will be facilitated by a member of the study team. Those who decline to be part of the CAPS group will still be able to access their medication through the CHIEDZA service (but not through the CAPS groups) if they choose to do so.

VL testing at 6 months and a year after ART initiation will be done by the CHIEDZA team, obviating the need to attend the clinic. At the end of the study, it is anticipated that CAPS groups will continue; the alternative will be to receive ART through health facilities.

Youth with HIV who access ART through CHIEDZA and become pregnant during the study will be supported to link with ANC services and will have the option to continue receiving their ART through the CHIEDZA service. All HIV-positive clients will be offered the opportunity to sign up to be part of the "CHIEDZA club". Those who sign up to the club will receive SMS messages including reminders about the CAPS group meetings, health messages, (in multiple forms such as straightforward information, games, quizzes, competitions, etc.) and encouragement/social messages

iv. 3.2.4 SRH and HIV prevention

Safe and easily accessible services will be identified in each of the study clusters and expedited referral will be arranged for youth requesting VMMC.

Syndromic management of STIs based on national guidelines will be provided. Syndromic management has been standard of care in many LMICs due to the resource constraints for laboratory testing for STIs including lack of adequately trained personnel, laboratory infrastructure and robust quality control. The main limitation of the syndromic management approach is that individuals with asymptomatic STIs (the majority) are not identified.[46] The prevalence of STIs in

southern Africa is high among youth and untreated STIs result in considerable morbidity, and at a public health level, STIs facilitate HIV transmission.[47]

In recent years, near point of care tests of STIs have been developed, including a test for GC and CT that utilises the closed GeneXpert system. The GeneXpert platform was first introduced for TB diagnostics and is widely available. The test is performed on a urine sample with minimal processing required.[48,49] In addition, a lateral flow assay for Trichomonas *vaginalis,* is a point of care test that is performed on self-taken vaginal swabs.[49,50] CHIEDZA will offer testing for GC and CT (male and female clients) and for TV (females only), contingent upon availability of test kits.

Other services include advice and information on MHM and provision of MHM products (e.g. menstrual cup, reusable pads and disposable pads and analgesia - see details of the MHM services provided in Section 5.4.3), condoms, pregnancy testing, contraception including LARCs (emergency contraception, depot injections, implants, Intra-uterine contraceptive devices (IUCDs), and the oral contraceptive pill) and risk-reduction counselling. In cases of IPV or sexual assault, youth will be referred for onward care. Referral pathways will be established to ensure prompt referral.

There is growing evidence from randomised clinical trials that use of oral daily PrEP confers significant protection against HIV acquisition, but it is currently unclear how the results of those trials will be incorporated into national guidelines.[51]

New developments will be reviewed during the course of the trial. If the provision of PrEP is incorporated in local guidelines during the course of the trial, the intervention will be adapted appropriately.

v. 3.2.5 General health information and counselling

IEC materials about SRH, HIV and general health will be available in the form of video clips and information leaflets... Brief general health counselling and referral for defined services e.g. mental health services (depending on availability) will be provided.

g. *3.3 Delivery of Intervention*

i. 3.3.1 Configuration of Services

The intervention will be delivered in a community, multi-purpose use location within each cluster, such as a community centre (so that clients are not marked out). This will be accompanied by outreach to advertise the service and sensitise youth to achieve high coverage. Outreach will be conducted locations frequented by youth (e.g. shopping centres, workplaces, bars, sports venues). Social mapping will inform where the optimum community sensitisation spots will be found.

The intervention will be “youth friendly” i.e. able to effectively attract youth, meet their needs comfortably and responsively and retain them in care. This will focus

on both the configuration of the intervention as well as the intervention providers.

Specifically, the service will be confidential and clients will be registered using a fingerprints biometrics system. Each subsequent attendance will then be recorded using fingerprints only. The table below summarises how the intervention will be configured to be youth friendly:

Table 2: Configuration of the intervention to provide Youth-Friendly Services

**Environment**	• Convenient location<br>• Adequate and attractive space where youth can socialise<br>• Comfortable surroundings<br>• Configured to attract male youth and female (e.g. male and female service providers)<br>• Providers known by first names<br>• Encouragement of youth-focused "non-health" activities e.g. music, dancing, sport, games, talks<br>• Outreach - sensitisation

Table 2 (continued): Configuration of the intervention to provide Youth-Friendly Services

**Confidentiality and privacy**	• Visual and auditory privacy<br>• Names not mentioned/ recorded*<br>• Anonymity of service accessed*
**Information**	• Paper and electronic IEC materials (leaflets, posters, videos) appropriate for various age and literacy levels
**Flexibility**	• Services will be provided from 11am to 7pm and at weekends (e.g. CAPS groups)<br>• Drop-in service delivery (no appointments)
**Youth as equal partners**	• Youth involved in design of space and intervention content and delivery<br>• Youth Advisory Board for feedback
**Appropriate and affordable services**	• Wide range of services available (see above)<br>• Services free of charge<br>• System to "red-flag" youth with concerns<br>• Well-established linkages to other services

**except where required for clinical care e.g. registration with national HIV care programme*

The intervention team will consist of a nurse, CHW and youth workers. The team will consist of a mix of male and female providers. There will be one intervention team per province, each responsible for delivering the intervention to 4 clusters. Each intervention team will rotate around its allocated clusters on a weekly basis (one day a week per cluster). Although the intervention teams are affiliated with this research project (and this will be made clear to all those who interact with them), their role will be to primarily to deliver what is recognized by the WHO as a 'best practice' public health intervention. Hence for this project we regard the intervention teams as separate from the 'research teams' and believe that the norms and standards governing their activities should largely be those accepted

for the implementation of public health interventions rather than those applied to conventional clinical research projects.

The intervention team will be selected on the basis of prior experience of working in communities and with youth. In addition, the intervention team will undergo a training programme on provision of youth friendly services as well as on each of the intervention components. Training will particularly focus on the following aspects:

1. Adolescent physiology and development
2. Knowledge and skill on delivery of each intervention component according
   to age and maturity of client
3. Referral pathways
4. Skills on counselling and communication with youth
5. Skills to bring myths to surface, to discuss and dispel them
6. Interpersonal skills
7. Attitudinal training (respect, confidentiality, non-judgement, relatability)

ii. 3.3.2 Collaborations and partnerships

The study team will collaborate with partners to facilitate an adequate supply of condoms, STI treatment, contraception, MHM products, clinical supplies including blood tests and drugs for individuals with HIV; and approval of use of health centre data.

• 4. Study Population

h. 4.1 Selection of Study Clusters

The study will be conducted in 3 provinces: Harare, Bulawayo and Mashonaland East. Harare is the capital and largest city and the population is predominantly of Shona ethnicity; Bulawayo, the second largest city in the country is situated about 450km west of Harare and is predominantly Ndebele. Mashonaland East borders Harare and peri-urban settings in this province were selected.

The first stage of the cluster selection was to define the criteria / requirements for each cluster. These were defined by the Trial Management Team as:

- An estimated cluster size of between 2000 – 4000 youth (to get a *minimum of* 1500 young people). This population size would allow for overestimates from census data, migration and so on.
- A cluster to be served by a defined clinic, and the clinic not serving another cluster.
  The clinic would ideally be sited within the cluster, but not necessarily so.
- Geographical separation between clusters to minimise contamination.
- Each cluster in an urban or peri-urban setting (as population densities in rural areas are very low)

Geographical Information (GIS) data which included spatial and attribute datasets (tabular data) were provided for the study area by the Zimbabwe

Electoral Commission. These included: electoral wards (with the population), health centres (with names and type), and schools (with name and type). The population for the study area was based on the Zimbabwe 2012 Census. The population within the study age group was assumed to be 20% of the total population based on the 2012 Census percent composition of the population (15-19yrs: 10.8% and 20-24yrs: 9.2%).

These data and local field knowledge were used to define a list of possible locations for clusters. The rough locations were examined in more detail, overlaying ward populations, clinic and school locations on top of Openstreetmap data which shows the roads, rivers, and, for some locations, all buildings. The actual cluster was defined by taking the electoral ward boundaries as starting points, to identify areas within the required population range required. These areas were then modified to take into account natural breaks for e.g. a river or a major road to provide sensible cluster boundaries. Where possible, natural boundaries such a green space or industrial areas, were used to form the edge of the cluster to minimise contamination. In some areas, where wards contained far large populations than 4000 young people and where all buildings were mapped, simple population estimation was carried out. This took the total number of buildings and multiplied it by 5.6 (a standard number used across Sub-Saharan Africa). This method of estimation was used to define an area that would contain between 2000-4000 young people. Once all the clusters were defined the boundaries and their clinics were exported to Google Earth (.kml format), to enable field teams to check the clinic locations and for key POIs to be mapped.

For mapping key POIs, the field team will walk around the entire cluster, marking any point of interest onto a print out of the openstreetmap data while at the same time storing the location and details as a bookmark in MAPSME. The electronic locations will be validated against the paper version locations.

i. 4.2 Randomisation

The first step in randomisation will be to obtain agreement from communities to take part in the study. Twenty-four clusters will be randomly allocated 1:1 to the intervention and standard of care arms. All clusters will be of approximately equal population size as a result of the mapping process used to define them. We will randomise clusters within each of three strata, defined by i) whether they receive healthcare from Harare municipal services (Harare City Health), Bulawayo municipal services (Bulawayo) or non-municipal (MoHCC) services (Mashonaland East), ii) HIV prevalence and iii) viral suppression.[3]. We will aim for equal numbers of clusters per stratum although this will depend on availability of suitable clusters/stratum. Within each of the 3 strata, we will randomise 1:1 to the control or intervention arm

Figure 3: HIV prevalence among 15-64 year olds by province (ZIMPHIA estimates)

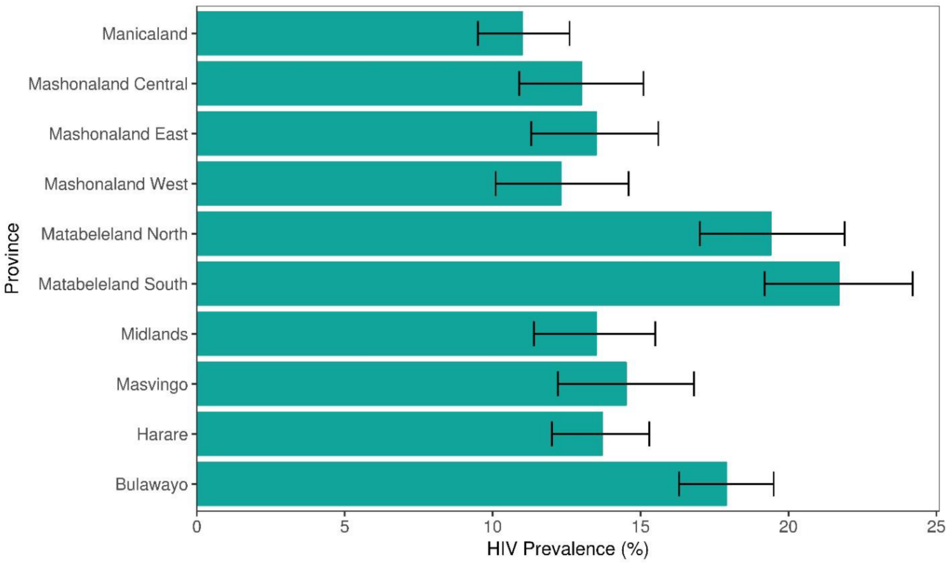

Figure 4: VL suppression (VLS) among 15-64 PHIV year olds by province (ZIMPHIA estimates)

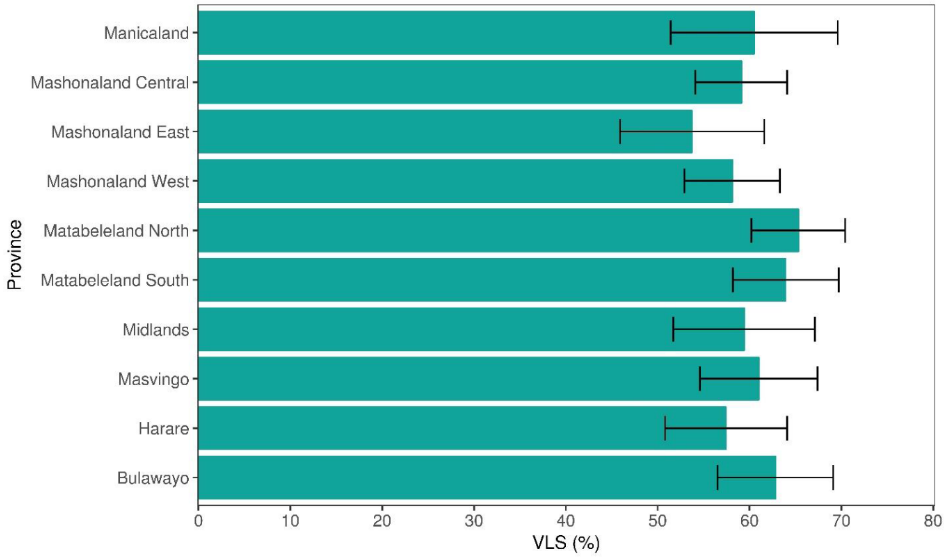

To maximise transparency and buy-in from stakeholders, a semi-public randomisation procedure will be carried out with representatives of the community, MoHCC and respective City Health or town council health departments.. After initiation of the intervention, if any community needs to be removed from the study (e.g. if a community should cease to agree to participate in the study) then the study leadership will decide upon the most appropriate course of action, which would likely include replacement of the community.

j. 4.3 Community Engagement

This study will build on the community engagement established during the formative work for the CHIEDZA trial conducted between 2017-2018. Briefly, this comprised a detailed stakeholder analysis (meetings and discussions with stakeholders including health services, CBOs, NGOs), and interviews and discussions with youth, family members and other community gatekeepers.

These informed the choice of implementing partners for the intervention (e.g. partners to whom CHIEDZA clients will be referred to for specific services).

Workshops were held with youth selected from study communities to inform the design, content and delivery of the intervention and to ensure they are equitable partners in the research process. Workshop participants were identified through relevant CBOs, health facilities and through hour existing networks. Each stratum will be served by a YAG, selected from those who attend the workshops. The YAGs will provide guidance on the implementation of the intervention and on study procedures, and represent the views of youth- the beneficiaries of this research. Strong community engagement will allow the establishment of a partnership between communities, participants and researchers to ensure the latter discharge their responsibilities ethically in the study communities. Community engagement will be an ongoing process through regular contacts with stakeholders and the YAGs. A dedicated CHIEDZA community engagement team will work with various community groups and stakeholders such as churches, schools, law enforcement, government structures at community level, health-related committees, and development-related committees. The main role of the community engagement team is to maintain an ongoing dialogue between researchers and community groups. Engagement will be in the form of community meetings, workshops, participation in stakeholder meetings and relevant forums and importantly maintaining a regular presence in the intervention communities. This will enable the study management team to ensure that information about the study is disseminated widely in the communities involved and to keep the community stakeholders updated regarding progress of the study, events that may arise in conduct of the research, and new developments in HIV prevention and treatment. Community engagement will allow researchers to get feedback from the community on social harms, individual and community level risks, perceptions about the study and implementation challenges. The YAGs and the community engagement team will remain involved throughout the study. The YAG members and the community engagement team will be trained on GCP and research ethics.

The local health services (e.g. the primary health care clinics) are the custodian of health of the community and collaboration with them is essential for ensuring both care of CHIEDZA clients beyond the intervention and to facilitate scalability. The intervention team will work with the nurses at the clinics to ensure that HIV and other services provided by CHIEDZA are integrated with routine services e.g. ensuring that all youth diagnosed with HIV through CHIEDZA are registered into the National ART programme. Regular meetings will be held between the intervention team and health facility staff to ensure that strong relations are maintained.

In addition, a key aspect of building confidence within communities is the knowledge that the study is being conducted in partnership and with the support of the government. Partnership with the MoHCC was sought at the inception of the study and input on the proposal was given by the MoHCC before the grant was submitted; the Deputy Director of the AIDS and TB Unit at MoHCC is a project co-investigator. Support and permission to conduct the study will be sought from

the Provincial Medical Directorate, District Medical Officers, Local Authorities, Health Services Directors and Town Secretaries for the smaller towns which do not have directors of Health Services. At community level, permission will be sought from the Provincial and District Administrators for use of community centres.

• 5. Research Procedures and Activities

k. 5.1 Prevalence survey

A community cross-sectional survey will be conducted among 18-24 year olds after 30 months of implementation of the intervention (700 randomly selected youth per cluster). The age group has been selected to ensure maximum exposure to the intervention, and to include only those able to give independent consent. A pre-intervention survey with HIV testing will not be conducted as this would itself constitute a mass testing programme and may compromise the anticipated intervention effect. The prevalence survey team will be separate from the intervention team, and will be blinded to whether the cluster is an intervention versus control cluster. However, the possibility of the survey team identifying the trial arm allocation for a particular cluster from other sources cannot be excluded.

The cross-sectional survey will be conducted in phases following the phased implementation of the intervention (Figure 3). Therefore, the first stratum of four clusters to implement the intervention and four control clusters will be surveyed over a three-month period. This will be followed by a survey of the intervention and control clusters in the second stratum over a period of three months. The clusters in the third stratum will then be surveyed. This will ensure that all intervention clusters are surveyed within a three-month period after the end of the intervention.

The sampling methodology will combine remote selection methodologies that use geographical information systems and satellite photographs and traditional random house selection.[52] Very high-resolution satellite images are frequently updated in Zimbabwe, and will be used to map every building in the clusters into Openstreetmap. This will be done remotely during the early stages of the project. The resulting Openstreetmap data will be checked by the field teams during the POI mapping process.

ARCGIS will be used to select random geopoints (defined as a short stretch of a street: about 100-200m) within each cluster. More random geopoints than required will be selected to ensure that enough are available during the survey. All eligible individuals whose household is on either side of the street will be approached for participation in the study. Selecting every eligible household member gives a self-weighting sample as every individual has the same probability of selection. Selecting households with equal probability as described above and then selecting ALL youth in the selected households is the simplest form of self-weighting design. In the event that there are no eligible individuals in the household the team will move on to the street. Every household in the stretch of street will be visited until 700 participants are enrolled.

Using the list of roads in the cluster, the survey team will go street by street and visit every household on both sides of the street All households in the designated stretch of the street will be visited to confirm that there is an eligible individual residing at the dwelling and if present to invite them to participate in the survey.

If the eligible individual is not present the field team will pre-book study interview time. The survey team will visit the household for the actual interview at the pre-booked time. If the eligible individual is not available on the booked appointment time and date, another appointment will be made for a second visit. If the individual is not available after three visits, no further attempts will be made for that individual. If the household is empty, then neighbours will be asked to provide information on the composition of the household. If it is clear that there are no eligible individuals living in the household, then the household will not be revisited. If there are or may be eligible individuals living in the household, then the household will be visited a further two times. If there is more than one eligible individual in the household, then all eligible individuals in that household will be enrolled.
Eligibility criteria will be:

- Age ≥ 18 years (day of their 18th birthday or older) to 24 years (one day before their 25th birthday)
- Written informed consent to complete the questionnaire and provide a blood sample

A study number will be allocated to each participant and a fingerprint will be collected. The fingerprints will be compared with the fingerprints of CHIEDZA service attendees to explore 1) the proportion of control cluster survey participants who accessed the intervention (degree of contamination) and 2) proportion of intervention cluster survey participants who accessed the intervention. Sociodemographic details will be collected and data on exposure to the intervention, and on duration of residence will be collected to assess in-migration, movement between clusters and likelihood of contamination.

Participants will be asked about knowledge of HIV status, history of HIV testing (including date of, place of and reason for test) and whether accessing HIV care/on ART, where they are accessing ART and ART adherence. Participants will be asked about their sexual behaviour (including VMMC (males only); pregnancy history, use of contraception and MHM (females only); and condoms (all)), and other risk behaviours such as smoking, alcohol and substance use and common mental disorders using validated tools.[53] Blood pressure, height and weight will be measured. Participants will be provided with information about risk behaviours and those who meet pre-defined criteria for common mental disorder or harmful substance use or severe hypertension will be referred for appropriate care. All participants who do not want to disclose their HIV-status or do not have documented evidence of an HIV-positive test and want to know their HIV status will be referred to the local health facility for HIV testing and counselling.. A blood sample for anonymised HIV antibody testing and an HIV VL (and drug resistance testing- will be collected. HIV VL will only be done on samples that test HIV-positive in the laboratory.

I. 5.2 Process Evaluation

A detailed mixed-methods process evaluation based on the MRC Process Evaluation Framework will address several research questions and key areas of

investigation related to three core process evaluation functions: implementation, mechanisms of impact and context, to provide guidance for sustainable and scalable implementation of the intervention should it prove effective.[39] The research questions / key areas of investigation, along with the related data collection methods and sources are outlined in detail in Table 4.

i. 5.2.1 Implementation

In evaluating the implementation of the intervention, the question 'what was implemented and how' will be answered. To do this, coverage, uptake and fidelity of the intervention will be assessed. Routine monitoring data from the CHIEDZA service (both fixed and mobile) will be used to determine the proportion of youth that accessed various components of the intervention (and how frequently), how equitable the coverage was and the proportion of HIV positive youth that enrolled in CAPS groups. Structured / unstructured observations (including mystery clients), intervention implementation logs, and in-depth interviews (IDIs) and / or focus group discussions (FGDs) with youth, providers and other key stakeholders will be conducted to more fully understand the implementation of the various components of the intervention, particularly barriers and facilitators of implementation fidelity, as well as coverage and uptake from various perspectives.

ii. 5.2.2 Mechanisms of Impact

The second core process evaluation function will investigate 'how the intervention leads to change'. This area will draw largely upon data collected through IDIs and / or FGDs with youth, providers and other key stakeholders, and will be supported by field notes and data from observations to understand which components of the intervention were best accepted and adopted, what challenges and barriers were faced and perceived impact the intervention had on the target population. It will also investigate how various components of the intervention interacted, and determine whether or not there were any unanticipated pathways or consequences.

iii. 5.2.3 Context

Finally, how local context affects implementation and shapes outcomes will be evaluated. IDIs and / or FGDs with youth, providers and other key stakeholders, observations and field notes will contribute to understanding what social, cultural, political, and logistical factors impede or facilitate how the intervention was implemented, and how users were able to engage with and adopt aspects of the intervention.

In March 2020, the WHO declared that the rapid spread of the SARS-CoV-2 infections (the causative agent of COVID-19) constituted a pandemic, and measures to contain the pandemic such as social distancing and lockdowns and restrictions on community gatherings were recommended. As part of understanding the overall context, the process evaluation will address the knowledge, attitude and practices around the pandemic and the effect of the pandemic on the implementation of the intervention. The intervention's current

configuration is at tension with the social/physical distancing measures that can contain COVID-19. Therefore, thoroughly understanding how CHIEDZA communities are interacting with and responding to the COVID-19 pandemic may be crucial to comprehending implementation and mechanism of change within CHIEDZA.

iv. 5.2.4 Data collection methods

The process data will be collected at multiple time points, using a range of methods and sources. There will be several sources of quantitative process data. To help interpret the results of the trial, existing services on HIV testing, ART delivery, SRH and other youth services will be mapped in the control and intervention communities. To the degree possible, the study team will obtain data from routine records in health facilities and other CBOs. To understand service use, the number of CHIEDZA service attendances and re-attendances and uptake of specific service components will be recorded (see Section 6.1.2). Specifically, data on type of contraception, number of referrals made (e.g. for VMMC, cervical screening, mental health services), number and type of STIs treated will be collected for CHIEDZA service users. These will not be individual level data but available by intervention cluster, age and sex. HIV care data including incident clinical events, drug side-effects, referrals for additional care, VL and CAPS group attendance will be collected on an individual basis for CHIEDZA service attendees, using personal identifiers. Furthermore, data on coverage of the intervention and uptake of services will also be obtained from the endline survey.

The methods for qualitative data collection will include IDIs, FGDs and observations- including mystery clients. To reduce the volume of data collection, and burden on research participants, one data collection method may provide data for several research questions simultaneously. Purposive and theoretically informed sampling will be used to determine the sampling frame for qualitative data collection methods, with consideration of trial community setting and provider and participant characteristics (e.g. age, sex, degree of engagement with the intervention). The selection of intervention communities will reflect the need to explore variation, with a detailed focus on a few communities in order to detail rich insights, while maintaining a manageable design. These will be chosen following randomisation. It is anticipated that up to 120 individual in-depth interviews with CHIEDZA service attendees from at least 3 intervention communities (in the three different strata) and up to 12 focus group discussions will be conducted. Up to 40 in-depth interviews with intervention staff from and up to 30 in-depth interviews and focus group discussions with community members will also be conducted in at least 3 different clusters representing the three different strata. A further 75 semi-structured observations of various components of the CHIEDZA intervention in at least 3 intervention communities in different strata will be conducted.

At least 6 mystery client observational visits of some CHIEDZA intervention components will also be conducted. CHIEDZA service attendees will be recruited

as mystery clients and invited to informational session about being a mystery client. The attendee's next CHIEDZA visit would then serve as a mystery client session as well. In this session, standardised clients will engage with the provider until they have received the advice or product they are seeking. After leaving the CHIEDZA centre/visit, the client will take down notes about the knowledge, attitudes, and practices of the provider; and then a follow-up interview will be conducted after the visit, preferably within 48hrs, to mitigate recall challenges. The interview will focus on the user experience of CHIEDZA service provision. The intervention team will be informed of these visits but would not know which of the young people at CHIEDZA a mystery client is. Because the mystery clients are already CHIEDZA attendees, the existing informed consent forms will be used to gain consent. Post-visit interviews will be tape-recorded, transcribed, and translated where necessary. Transcription, translation, coding, and analysis will take place the same as for the IDIs and FGDs.

Throughout the trial, the process evaluation will remain flexible and dynamic to be responsive and adapt to emerging trial and process findings and so additional data collection may be conducted to investigate emerging findings. Data will be triangulated from multiple sources, and quantitative and qualitative analyses will build upon and reinforce each other.

Table 3: Process Evaluation Summary

Research Domain		Research Questions / Key Areas of Investigation	Data Collection Methods and Sources
**Implementation** *What is implemented and how?*	**Coverage and Uptake**	What proportion of youth accessed various components of the intervention?<br>How equitable was this coverage?<br>What were the barriers and facilitators to coverage and uptake?<br>What proportion of HIV positive youth enrolled in CAPS groups?<br>Did any youth from control communities access the intervention?	Routine monitoring data<br>Structured / unstructured observations<br>Mystery Clients<br>Intervention implementation logs<br>IDIs and / or FGDs with youth, providers, family members of youth, community members and other key stakeholders
	**Fidelity**	How did a) training, and b) actual implementation of each component and delivery-mode of the community-based intervention vary from what was planned?<br>What were the barriers and facilitators to implementation fidelity?<br>What adaptations were made?	

**Mechanism of Impact** *How does intervention lead to change?*	**Responses to and interactions with the intervention**	Which components of the intervention were best accepted and adopted by youth and other stakeholders?<br>What were the barriers and facilitators to acceptability?	IDIs and / or FGDs with youth, providers, family members of youth, community members and other key stakeholders<br>Structured / unstructured observations<br>Field notes
	**Interactions and Consequences**	How did various components of the intervention interact?<br>Were there any unanticipated pathways or consequences?	

Table 3 continued): Process Evaluation Summary

Research Domain		Research Questions / Key Areas of Investigation	Data Collection Methods and Sources
**Context** *How context affects implementation and shapes outcomes?"*	**Proximal and Distal**	What social, cultural, political, and logistical factors impede or facilitate (or were affected by) how the intervention was implemented, and how youth and other stakeholders engaged with it?<br>What were contextual reasons for adaptations to the intervention and its delivery?	IDIs and / or FGDs with youth, providers, family members of youth, community members and other key stakeholders<br>Structured / unstructured observations<br>Field notes

m. 5.3 Cost-effectiveness studies

i. 5.3.1 Introduction

Costing and cost-effectiveness studies have become an essential component in the evaluation of health interventions especially in resource-constrained settings. The information generated can inform policy makers on the cost of scaling-up interventions and whether they provide efficient use of the limited financial resources available to them. Complex public health interventions like the one proposed are challenging to undertake an economic evaluation of. The multiple components of the intervention need to be costed and impact of each component on the final outcome (HIV-positive youth virally suppressed on ART) accounted for. In this scenario decision-analytic modelling provides a rigorous approach to comparing the costs and health consequences of an intervention to existing approaches to providing care thereby informing policy makers of the

additional costs and additional health gains achieved through real-world implementation. An advantage of decision-analytic modelling includes the ability to evaluate cost-effectiveness over a longer time-horizon than that observed in the research study. Decision-analytic models are reliant on data commonly referred to as input parameters. It is these input parameters that allow simulation of the long-term costs and health consequences of an intervention and thereby estimating its cost-effectiveness in comparison to standard of care.

ii. 5.3.2 Aims and Objectives

Broadly the aim is to undertake a decision-analytic model-based cost-effectiveness analysis of the CHIEDZA intervention. The health economics research proposed in this study aims to undertake primary costing studies of the different components of the CHIEDZA intervention and current approaches to providing HTC/ART care, and synthesise these data with findings observed in the parent cluster randomised trial and process evaluations within a modelling framework.

Primary objective:

- To estimate the incremental cost per DALY averted from implementing the CHIEDZA intervention

Secondary objectives:

- To estimate the costs of HIV testing and counselling through the CHIEDZA intervention and through routine services
- To estimate the cost of linkage to HIV care and treatment through the CHIEDZA intervention
- To estimate the cost of providing ART (including costs of drugs, delivery and monitoring)
- To estimate the cost of adherence support provided by the CHIEDZA intervention
- To estimate the cost of the SRH and HIV prevention provided by the CHIEDZA intervention

iii. 5.3.3 Primary costing studies

Costing of the different components of the CHIEDZA intervention and of routine HIV testing and treatment services will follow standard UNAIDS 2011 guidance.[40] For each 'service' to be costed a combination of top-down and bottom-up costing will be utilised to first estimate the total cost of that service component. For each service this involves identifying all the resources (Inputs: e.g. staff, training, utilities, consumables, equipment) used in delivering the service. We will then obtain the price/cost of this resource through discussions with the finance department or through the use of international market prices (e.g. for drugs, consumables/equipment) in order to estimate the total cost. The total cost will include all resources used directly at the service centre and those provided indirectly through support centres (e.g. centrally provided services). The total cost will be linked to the usage of that service component (e.g. total number of

youth managed) to estimate unit cost (e.g. cost per youth managed). Table 4 provides an overview of how each component of the CHIEDZA intervention or routine HIV testing and treatment will be costed to estimate the unit costs.

Table 4: Overview of costing approach

UNAIDS Costing process	Data source
**1. Define and measure services and output by service centre**	Service centres include:<br>• Individuals components of CHIEDZA intervention<br>• HIV testing and treatment services<br>Review findings from process evaluation or access medical records from health facilities
**2. Define and measure inputs used at the service centre**	Interviews with ward staff<br>Interviews with finance department<br>Inputs include:<br>• Staff<br>• Consumables<br>• Equipment<br>• Capital and other overheads

Table 4 (continued): Overview of costing approach

UNAIDS Costing process	Data source
**3. Measure the cost and prices of inputs at service centre**	Interviews with finance department
**4. Define, measure and cost the input used at the support centres**	Support centres include centrally provide services essential for running of service centre e.g. transport; Monitoring and Evaluation; medical waste disposal.<br>Interviews with heads of support centres<br>Interviews with finance department
**5. Calculate the unit cost of each type of output for each support centre**	Interviews with heads of support centres to define approach and proportion of costs allocated to the service centre of interest
**6. Allocate costs from the support centres to care centres**	
**7. Calculate the total cost for each service centre**	For each service centre combine the total cost of inputs used at service centre to the support centre costs allocated to the service centre
**8. Calculate the unit costs for each service centre**	For each service centre divide the total cost by the total output

iv. 5.3.4. Analytical plan

Decision-analytic modelling will be utilised to estimate the incremental cost-effectiveness of the CHIEDZA intervention. Previously developed HIV testing and treatment models will be adapted to undertake the cost-effectiveness analysis.[54] The model will reflect the youth population at the CHIEDZA study sites. For this data on participants including age/sex distribution, HIV prevalence and ART coverage will be extracted from the post-intervention cross-sectional survey

undertaken amongst control clusters in the CHIEDZA trial. CD4 count distributions in this age-group will be obtained from published data. The model will also be parameterised with cost and outcomes data generated from the CHIEDZA trial, process evaluations and primary costing studies. DALY weights and other input parameters will be obtained through the process of multiparameter evidence synthesis. Briefly this involves undertaking targeted systematic reviews of the published literature.

The cost-effectiveness analysis will adhere to the Gates reference case and will simulate the lifetime costs and DALYs for current standard of care and under the scenario the CHIEDZA intervention is implemented. The incremental costs and incremental DALYs averted through implementation of the CHIEDZA intervention will allow estimation of the ICER. Although there are no formal ICER thresholds for interpreting cost-effectiveness in Zimbabwe and other sub-Saharan Africa countries, informal thresholds relating to the one times and three times the gross domestic product will aid decision-making on cost-effectiveness. Additionally, ICERs will be presented on cost-effectiveness acceptability curves to allow interpretation of cost-effectiveness at different willingness to pay thresholds for a DALY averted. Probabilistic sensitivity analysis will be undertaken to incorporate uncertainty in values utilised for input parameters in the model. Scenario analysis will explore cost-effectiveness of different time horizons.

n. 5.4 MHM Intervention Acceptability, Uptake, and Effectiveness (MAE) Sub-Study

The CHIEDZA intervention will provide young women access to a choice of MHM products, analgesia, and education on pain management, menstruation, and MHM more generally. A prospective cohort sub-study will be nested within CHIEDZA to understand perceptions and practices around MHM and knowledge on menstruation and MHM, and to investigate the acceptability, uptake, and effectiveness of an informed MHM intervention aimed at providing access to MHM education, MHM product choice, and analgesia among young women.

i. 5.4.1 Background and Rationale

The onset of puberty and menstruation is a fundamental experience for girls and women, and appropriate menstrual hygiene management (MHM) is directly linked with basic human rights. Yet, MHM continues to be a challenge for many girls and women, especially in LMICs.[55] Due to a lack of access to appropriate education, affordable and accessible menstrual hygiene care products, and adequate sanitation, many women especially girls and young women during the first few years following menarche struggle to manage menstruation safely and/or with dignity.[56] In LMICs, many girls and women have no access to clean water or private spaces to attend to their menstrual care needs and are forced to use non-absorbent, unhygienic, and uncomfortable materials that may negatively affect their health, education, and well-being.[55] In 2012, the Joint Monitoring Program of the WHO and UNICEF defined adequate MHM as access to clean absorbents including, when relevant, sufficient washing, drying, storage and

wrapping of reusable absorbents; adequate frequency of absorbent change; washing the body with soap and water; adequate disposal facilities; and privacy for managing menstruation. Using the MHM definition as a measure, studies have shown that many girls and women in LMIC have inadequate MHM and inadequate MHM may hinder their daily activities, prevent girls and women from going to school or participating in income generating activities, or lead to stigma, discrimination, and adverse psychosocial outcomes.[57] Options for menstrual health products include disposable and re-usable pads, tampons, period pants, and the menstrual cup. However, there are relatively few data on acceptability, uptake, and effectiveness of these products (especially reusable products which are more cost-effective and environmentally friendly) in LMICs. A study to investigate this will be nested within the CHIEDZA trial.

ii. 5.4.2 Study Aim and Objectives

The aim of the MHM sub-study is to investigate the acceptability, uptake, and effectiveness of an MHM intervention within the community-based CHIEDZA trial among women aged 16-24 years.

The specific objectives are

1. To understand and describe the current knowledge levels, practices and perceptions around menstruation and MHM among women aged 16 – 24 years in Zimbabwe.
2. To investigate menstrual product choice, continued use or discontinuation of products, and patterns of use over time among young women offered all 3 product choices

Effectiveness

3. To investigate the effect of the intervention on MHM practices e.g. access and use of clean absorbents, sufficient washing, drying, storage and wrapping of reusable absorbents; washing the body with soap and water
4. To investigate the effect of the MHM intervention on the knowledge and perception of menstruation and MHM
5. To investigate the effect of the MHM intervention on pain management during menstruation

Acceptability

6. To investigate the acceptability of the intervention in a community-based SRH intervention programme i.e. intervention uptake, affective attitude, burden, ethicality, intervention coherence, opportunity costs, perceived effectiveness, self-efficacy, and intervention implementation challenges, fidelity, reach, and service quality[58,5958,5958,5958,5958,5958,5958,59]

Objective 1 will be met by using CHIEZDA data and by carrying out qualitative data research with female CHIEDZA participants that uptake MHM services. Quantitative data collected from the nested cohort will also contribute to objective 1. Objectives 3-6 will be met by carrying out qualitative and quantitative data research within the nested cohort study.

iii. 5.4.3 Theory of change

A theory of change model has been developed to illustrate the expected outcomes and impact from the MHM sub-studies (Figure 5).

iv. 5.4.4 MHM Intervention

All CHIEDZA attendees will have *a choice of one of the following MHM products*: the menstrual cup or reusable pads. This will be accompanied by analgesia (12 tablets of paracetamol or ibuprofen), a bar of soap, a simple period-tracking sheet, and comprehensive MHM education including an MHM educational pamphlet participants can take home and an MHM educational video to be played at the CHIEDZA centre. Clients will be able to ask for a different product but only after 1) a minimum of 3 months of usage and all CHIEDZA female participants who choose an MHM product will have the option of changing their chosen MHM product if they bring the existing MHM product back to the CHIEDZA centre.

Participants in the nested cohort study will be given comprehensive MHM education including an MHM educational pamphlet participants can take home and an MHM educational video to be played at the CHIEDZA centre, a monthly supply of up to 12 analgesics (ibuprofen or paracetamol), a comprehensive period tracking diary, and *all the MHM products* as summarised in Table 5. Participants in the cohort will be recruited from 2 randomly-selected CHIEDZA intervention clusters in Harare and will be followed-up 3 monthly for one year. At enrolment, the 6 month, and 12 month visit, participants will be required to self-complete a questionnaire (using Open Data Kit on a tablet) and at the 3 month and 12 month (endline) visit, participants will be required to hand-in their period tracking diary for review.

Figure 5: Theory of Change model for the MAE sub-study

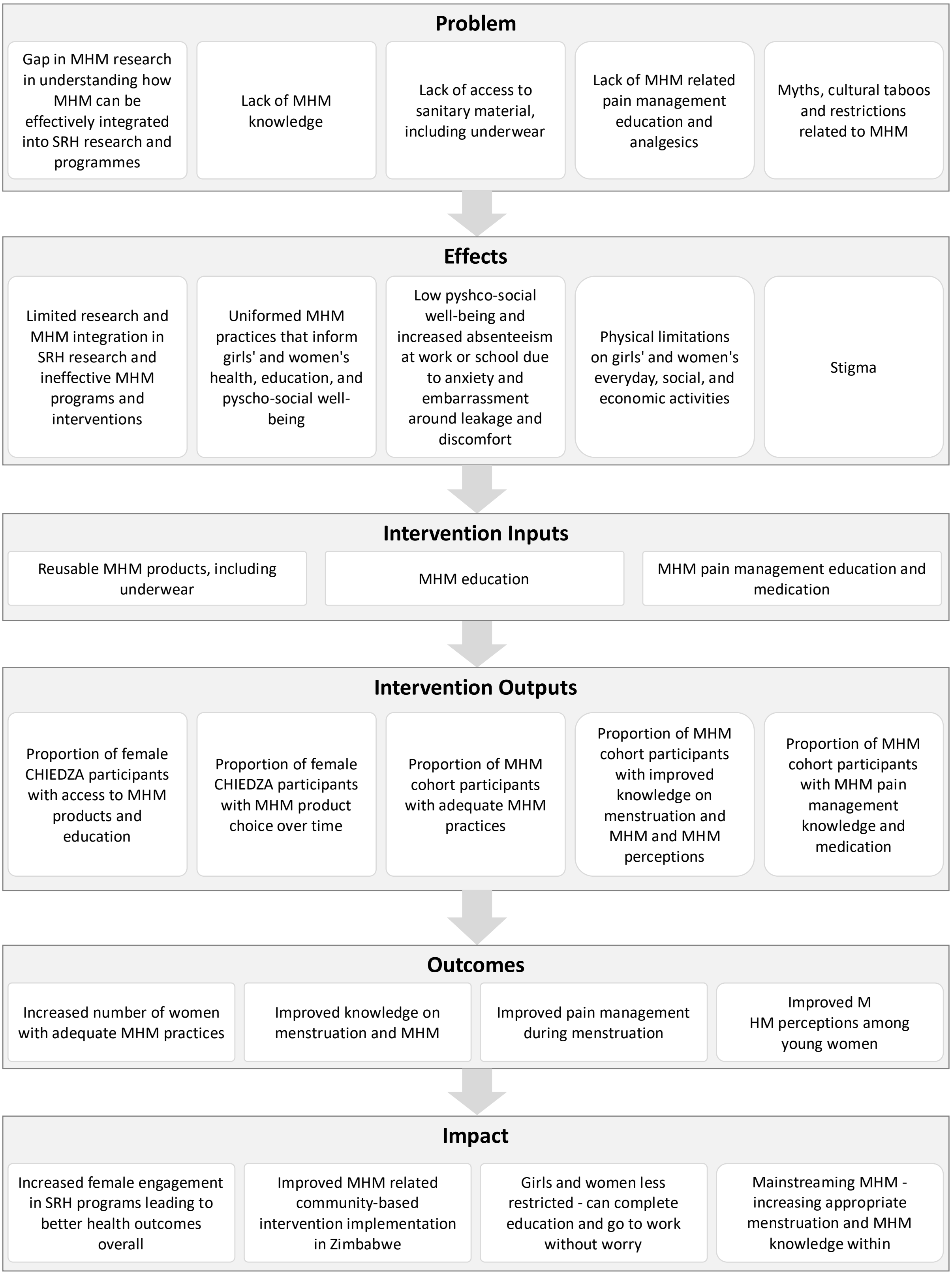

Table 5: MHM Intervention

<table>
<tr><th>MHM Product</th><th>Hardware</th><th>Software</th></tr>
<tr><td>Menstrual Cup</td><td>• 1 cup
• 1 pack of disposable pads (1 month supply)
• 2 pairs of underwear
• Soap
• MHM educational pamphlet
• Pain medication</td><td rowspan="2">• Initial education session:
– One-on-one product demonstration
– Pain management information
• Scheduled education sessions throughout intervention period that explore myths and taboos and elaborate on puberty, menstruation, and menstrual hygiene
• MHM Video</td></tr>
<tr><td>Reusable Pads</td><td>• 1 pack of reusable pads (AFRIPads with 4 reusable pads/pack or Days for Girls with 2 period shields and 8 period liners/pack)
• 2 pairs of underwear
• Soap
• MHM educational pamphlet
• Pain medication</td></tr>
</table>

v. 5.4.5 Study Outcomes

Objective 1: *To understand and describe the current knowledge levels, practices and perceptions around menstruation and MHM among women aged 16 – 24 years in Zimbabwe.*

Outcome 1: Qualitative data from the FGDs and descriptive data from the quantitative questionnaire will be analysed with proportion and cross-tabulations to show the distribution of knowledge, perceptions, and practices regarding MHM. Data from the FGDs will complement the quantitative data.

Objective 2: *To investigate menstrual product choice, continued use or discontinuation of products, and patterns of use over time among young women offered all 3 product choices*

Outcome 2: Menstrual product choice over time amongst young women and proportion of young women with continued or discontinued menstrual product use over time. Data from the FGDs and IDIs will complement the quantitative data

Objective 3: *To investigate the effect of the intervention on MHM practices e.g. access and use of clean absorbents, sufficient washing, drying, storage and wrapping of reusable absorbents; washing the body with soap and water*

Outcome 3: Using the quantitative data collected from the period tracking diary entries and the pre- and post- quantitative questionnaires, the self-reported MHH practices will be used to generate a pooled, aggregate of adequate MHM practice consistent with the definition given by the WHO and UNICEF[57] and the theory of change model

developed for the longitudinal cohort sub-study intervention. Data from the FGDs and IDIs will complement the quantitative data

Objective 4: *To investigate the effect of the MHM intervention on the knowledge and perception of menstruation and MHM*

Outcome 4: Pre- and post-intervention quantitative data from the questionnaires will be used to analyse change in the proportion of cohort participants with improved MHM knowledge and perceptions over time

Objective 5: *To investigate the effect of the MHM intervention on pain management during menstruation*

Outcome 5: Data collected from the period tracking diary and the pre- and post-intervention questionnaires will be used to analyse change in pain management in cohort participants.

Objective 6: To investigate the acceptability of the intervention in a community-based SRH intervention programme i.e. intervention uptake, affective attitude, burden, ethicality, intervention coherence, opportunity costs, perceived effectiveness, self-efficacy, and intervention implementation challenges, fidelity, reach, and service quality[58,5958,5958,5958,5958,5958,5958,59]

Outcome 6: An assessment of MHH intervention acceptability i.e. affective attitude, burden, ethicality, intervention coherence, opportunity costs, perceived effectiveness, self-efficacy, and intervention implementation challenges, fidelity, reach, and service quality, using quantitative data on MHH intervention uptake and a qualitative data assessment from participants and the intervention team

vi. 5.4.6 Data from the CHIEDZA intervention

Quantitative Methods

Quantitative data from all female participants that accessed MHM services across the 12 CHIEDZA intervention clusters will be used to generate yield data on MHM intervention uptake (Objective 6) and MHM product choice over time (Objective 2). MHM product choice over time will be assessed using data from SIMPRINTS biometric system which will record every time an existing female CHIEDZA participant returns an MHM product of choice and requests to uptake a different MHM product (Objective 2).

Cross-tabulation tests will also be run to assess the association between some socio-demographic variables such as age with MHM intervention uptake and MHM product choice. Informing factors and reasons for MHM intervention uptake and changes in MHM product choice will then be further explored qualitatively within the two randomly selected nested MHM sub-study intervention clusters from the CHIEDZA intervention province of Harare.

Qualitative Methods

Up to 12 FGDs will take place throughout the delivery of the intervention with at least 2 FGDs in each of the nested MHM sub-study intervention clusters at baseline, 6 months, and 12 months. A purposive sample of 12 – 15 participants will be selected to participate in FGDs to include those of varying ages, females using different types of MHM products, and females that have changed their MHM product of choice over time. Written informed consent will be obtained from each participant before the FGDs.

The FGDs will be conducted by local female qualitative research assistants in either Shona, Ndebele or English (as agreed by the participants) and will be audio recorded. Each discussion is expected to take between 1 – 2 hours. Discussions will used semi-structured topic guides will be used to explore current knowledge on menstruation and MHM and practices and perceptions around MHM (Objective 1). The FGDs will also be used to investigate the factors that may inform product choice and continued or discontinued menstrual product use over time (Objective 2) and the acceptability of an MHH intervention within CHIEDZA (Objective 6). These FGDs will be followed-up with IDIs to further explore individual views on menstrual product choice and use over time. Descriptive narratives, from participant observations at the CHIEDZA sites, will also be used to assess MHH intervention fidelity and service delivery quality (Objective 6)

Throughout the delivery of the intervention, some of the FGD participants may be invited for a follow-up IDI. Up to 10 IDIs may take place and qualitative data from these interviews will be used to compliment the data from the FGDs. Written informed consent will be obtained from each participant before the IDIs.

Audio recordings of the FGDs and IDIs will be transcribed verbatim and then translated (if applicable) into English for analysis. Thematic analysis and coding of emerging themes (common and divergent) will be conducted by 2 social science research assistants independently. Regular data analysis team meetings steered by a senior social scientist will be held to discuss and compare the work of the RAs. All recordings will be downloaded on to a password-secured laptop for processing purposes and then deleted once the nested cohort study is completed.

vii. 5.4.7 The Nested MHM Cohort Sub-Study Intervention

MHM Sub-Study Pilot

At the start of the trial, the tools and procedures will be piloted for the nested cohort study. In addition, qualitative research will be carried out to gain a better understanding of how participants experienced the MHM component during the pilot phase. The qualitative research during the pilot phase will include up to 2 FGDs (one each with young girls (16 – 20 years old) and older girls (21 – 24 years old)) with a purposive sample of 12 – 15 participants to include those of varying

ages and those using different MHM products. Tools and procedures will be revised based on the results and experiences of the pilot.

Recruitment

Two CHIEDZA intervention clusters from Harare district will be randomly selected for the cohort sub-study. Using convenience sampling, the first three hundred (combined) female CHIEDZA participants seeking MHM services in the selected clusters will be recruited for the cohort and followed for 1 year. Eligible young women who express an interest in enrolling in the cohort will be given information about the study and will be provided the opportunity to ask questions. Inclusion criteria will be willingness to participate and the ability to provide signed informed consent. Exclusion criteria will be the following: current pregnancy or intending to get pregnant within the next 1 year; irregular or absent bleeding within the last 3 months; or having any condition that, in the opinion of the investigator, would preclude informed consent or make study participation unsafe. After all questions have been answered, eligible young women will be asked to sign a consent form. Participation in the cohort will be optional and it will be emphasized that care of those who decline to participate will not be adversely affected. Participants will also be allowed to withdraw at any time and will not be adversely affected.

Enrolment

At enrolment, consenting participants will receive the MHM intervention including MHM education, analgesics, 2 pairs of period pants, and all the MHM products in Table 5. Participants will also be asked to complete a self-administered structured quantitative questionnaire using ODK on a tablet to obtain data on socio-demographics, and genital and menstrual hygiene, knowledge on menstruation and MHM, MHM perceptions and practices, pain management, and psycho-social well-being. Each participant will also be given a comprehensive 4 month period tracking diary. The participants and RAs will also go through the participants' period tracking diaries together to make sure participants are comfortable and confident using the diaries and that the diaries are being completed accurately on a monthly basis.

Participants will also be given a participant booklet with the dates of their follow up visits (with a two-week window period around their scheduled follow-up visit date). If participants fail to attend their CHIEDZA site visit, they will be contacted and asked if they can be visited at home.

Follow-up

Participants will be followed up every 3 months for one year. At baseline, 6 months, and at 12 months (endline), participants will complete the same self-administered quantitative questionnaire to obtain updated data on genital and menstrual hygiene, MHM knowledge, perceptions, and practices, psycho-social well-being and period pain management knowledge. At the first 3-month follow-up visit, the first period tracking diary will be collected. Participants will be given another 4 month period tracking diary at the 9 month visit that will be collected at the 12 months visit.

If participants fail to make their scheduled CHIEDZA site visits they will be contacted via phone (up to three times). If participants fail to reschedule their

follow-up visit at the CHIEDZA site or then choose to have a home visit, a trained research assistant will travel to participant's home, according to the address given. If the participant is not at home on the date of the scheduled visit, they will be contacted via phone (up to three times).

Sample Size

A sample size is based on Objective 3: 300 participants will provide 90% power to detect an increase in adequate HM practices from 10% to a double of 20% before (pre-) and after (post-) the intervention for each sub-study participant respectively.

Statistical Methods

Descriptive statistics will be used to show the socio-demographic characteristics as well as the distribution of knowledge, perceptions and practices regarding MHM. The self-reported MHM practices will be used to generate a pooled, aggregate of adequate MHM modified from the Joint Monitoring Program of the WHO and UNICEF in 2012.[57] The MHM sub-study intervention will only affect three components of the definition: access to clean absorbents including, when relevant, sufficient washing, drying, storage and wrapping of reusable absorbents; adequate frequency of absorbent change; and basic understanding of menstruation. The intervention will not affect washing the body with soap and water; adequate disposal facilities; or privacy for managing menstruation.

McNemar's test for correlated proportions and conditional logistic regression will be used to compare the proportion with improved MHM practices (Objective 3), proportion with good knowledge and appropriate perceptions (Objective 4), and appropriate pain management (Objective 5) pre- and post- the intervention.

Data from all of the period tracking diary entries will be used for descriptive statistics on MHM product choice and continued or discontinued product use over time, and MHM product patterns of use, MHM practices and pain management during menstruation (Objectives 2, 3, and 5). The diary entries will also track if participants missed work or school on the days before, after, or during their periods.

The facilitators, barriers, reasons for MHM product choice and continued and discontinued use of products over time and an of appraisal of MHM products from participants will be further explored using qualitative methods.

Qualitative Methods

A similar approach will be followed as for the qualitative study as described above (main CHIEDZA study qualitative interviews-see above).

Qualitative FGDs and IDIs will be used to explore the *how* and *why* research questions of the study. FGDs will be used to understand the broad social and environmental context of the study. The FGDs and IDIs will also be used to explore effects of pain during menstruation and the use of analgesics and pain management in general among the women (Objective 5). Semi-structured topic guides for the FGDs and IDIs will also be used to further explore the factors that inform menstrual product choice, continued or discontinued use over time, and

patterns of use (Objective 2) and the effects of the intervention on knowledge levels and perceptions (Objective 4), and MHM practices (Objective 3). Qualitative FGDs and IDIs will also be used to investigate MHM intervention acceptability by looking at affective attitude, burden, ethicality, intervention coherence, opportunity costs, perceived effectiveness, self-efficacy, and intervention implementation challenges (Objective 6).

To adhere to the prevention control measures implemented in the COVID-era, telephonic interviews may become a norm for IDIs (section 5.2.3). Where possible, every effort will be made to obtain written consent, that can be followed up with a phone-based IDIs. However, when not possible, phone-based consent will be sought. Measures to verify true client identity will include 1) video-conference calls where possible with participants 2) asking client name, age & where they stay in the CHIEDZA intervention communities

o. 5.5 STI Screening Acceptability and Effectiveness

A semi-qualitative study will be nested within CHIEDZA to i) assess current knowledge, attitudes and practices regarding STIs and perceived barriers towards accessing STI testing and partner notification; and ii) determine factors predicting acceptance of STI testing. STI testing will be offered (contingent on availability of test kits) and uptake prevalence and yield of testing will be assessed. .

i. 5.5.1 Background and Rationale

Youth are a group that is particularly vulnerable to STIs, including syphilis, gonorrhoea, chlamydia, trichomonas and HIV.[60] STIs facilitate HIV transmission and cause morbidity.
Both gonorrhoea and chlamydia can cause pelvic inflammatory disease in women, leading to infertility, risk of ectopic pregnancy or chronic pelvic pain.[61] In Zimbabwe, both are common amongst symptomatic patients. In a study of 200 men with urethral discharge, 73.5% had gonorrhoea, 22.5% had chlamydia, 4.0% had trichomonas and 3.5% had mycoplasma infection, and the prevalence of HIV was 41% among 489 patients presenting with urethral discharge, vaginal discharge or genital ulcer disease.[62,63]

In LMIC, syndromic management of STIs is the recommended strategy due to resource constraints in implementing aetiological diagnosis.[64] The number of studies assessing the perceptions and acceptability of STI screening is therefore limited. A study in Mombasa (that included only female youth) noted a general willingness for STI screening but barriers included lack of symptoms and stigma[65].[66] The screening for GC, CT and TV within CHIEDZA therefore represents an excellent opportunity to further build on this work to inform us on how to operationalise screening. The limitations of syndromic management were demonstrated in the Zimbabwe study. Among 200 patients with GUD, 27% had GC or CT but most of them did not meet the criteria for concomitant syndromic management. These patients would therefore remain untreated and continue to transmit infection.

Partner notification is integral to effective STI care to reduce infection rates in the population and to prevent both re-infection and the consequences of untreated infections. There are a number of approaches to partner notification, including patient referral (whereby patients are encouraged to contact their sex partners themselves), provider referral (the health care provider notifies the partner and arranges treatment), contractual patient–provider referral (a two-step approach that links patient and provider referral methods), and expedited partner therapy (the diagnosed patient takes the prescriptions or medication to his/her partner)".[60] However, the evidence is not present to identify a single optimal partner notification strategy for a particular STI.[67] Despite the importance of partner notification, it is often not performed adequately.[68,69] There are a number of potential reasons for this, including concerns about adverse partner reactions, such as risk of violence or of the partner leaving.[68] Embarrassment and stigma are also reported as perceived barriers to partner notification and partner notification is also lower in casual relationships.[70,71]

ii.5.5.2 Study Aim and Objectives

This sub-study aims to assess current knowledge, attitudes and practices regarding STIs; to assess knowledge, attitudes and perceived barriers towards accessing STI testing and partner notification; and to determine factors predicting acceptance of STI testing. The uptake, yield and prevalence of STI testing will be assessed in the intervention arm. In addition, we will sample a proportion of participants in the endline survey to investigate the impact of offering unselected STI testing on the population level prevalence of STIs.

The objectives are:

1. To assess knowledge, attitudes and practices towards STIs
2. To assess attitudes towards accessing STI testing including site of testing and mechanisms of receiving results
3. To assess attitudes and perceived barriers to partner notification for STIs
4. To determine uptake, yield and prevalence of STI testing
5. To determine factors predicting acceptance of STI testing
6. To determine prevalence of STIs by study arm

iii.5.5.3 Sub-study methods

Recruitment

Youth attending the CHIEDZA service sites will be eligible. Those attending the CHIEDZA service who express an interest in enrolling in the sub-study will be given the opportunity to obtain more information and ask questions. In stage 1 (Harare only), purposive sampling will be used to deliberately select equal numbers of male and female clients who did and did not agree to STI testing. In stage 2, consecutive sampling will select clients for questionnaire administration. Inclusion criteria will be age 16 to 24 years inclusive, willingness to participate and the ability to provide signed informed consent. Exclusion criteria include an inability to communicate or read in English or Shona. Participation in the sub-study will be optional and it will be emphasized that care of those who decline to

participate will not be adversely affected. Following this, STI testing will continue to be offered in the intervention clusters (Harare and Bulawayo).

Data Collection

In Stage 1, consenting participants will provide basic sociodemographic information. They will then undergo a semi-structured interview, collecting qualitative data on the following domains:

- Knowledge of sexually transmitted infections
- Attitudes towards STI testing
- Current practices regarding STI testing and treatment-seeking behaviour
- Attitudes and perceived barriers to partner notification

Stage 2 will follow completion of stage 1. Consenting participants will undertake a structured questionnaire. The exact questions and available options for the questionnaire will be refined based on the responses to the semi-structured interviews from stage 1. The questions will fall into the following domains:

- Sexual history including history of previous STIs
- Knowledge of STIs
- Attitudes and practices towards STI testing
- Attitudes and perceived barriers to partner notification

Qualitative methods

Participants will be purposively selected as described above for stage 1 of the sub-study. Data collection by semi-structured interviews will be performed until data saturation is reached.[72] Other qualitative studies assessing STI testing in young people noted data saturation following between seventeen and twenty-four interviews.[66] Our study will likely have a similar data saturation point. The semi-structured interviews will use semi-structured topic guides based on the domains noted above. They will be conducted in English or Shona, depending on the participants' preference. Written informed consent will be obtained from each participant before the interview. Interviews will be digitally recorded, and written notes will be taken. Each interview is expected to take between 30 minutes and 1 hour. Audio recordings of the interviews will then be transcribed verbatim and then translated (if applicable) into English for analysis. Thematic analysis will be performed to provide a qualitative summary of knowledge, attitudes and practices as follows: familiarisation with the data; generating initial codes; searching for themes; reviewing potential themes; defining and naming themes; and producing the report.[73]

Quantitative methods

In Stage 1 and 2, for the quantitative aspect, we anticipate recruiting 600 participants which will provide 80% power to detect an odds ratio of 2 for uptake of STI testing assuming a prevalence of the variable of interest in the control (non-STI testing) group of 30%. This assumes an uptake of STI testing of one third, giving 200 cases and 400 controls. Descriptive statistics will show the socio-demographic characteristics and distribution of knowledge, attitudes, and practices regarding STIs. Categorical variables will be described using

frequencies and percentages. Continuous variables will be described using either mean (standard deviation) or median (interquartile range) for normally distributed and non-normally distributed data, respectively. Logistic regression will be performed to determine factors predicting acceptance of STI testing.

For the prevalence survey, we anticipate recruiting 300 participants per cluster in the two provinces that will offer STI testing for at least 12 months before the endline survey is conducted (Harare and Bulawayo). Urine samples will be collected on randomly allocated days that the survey will be implemented. The days on which urine samples will be collected will be selected by block randomisation with 55% of survey days selected to allow for refusals etc.

iv.5.5.4 Outcomes

Outcomes will be:

1. Qualitative summary of knowledge, attitudes and practices regarding STIs
2. Qualitative summary of attitudes and practices regarding STI testing
3. Qualitative summary of attitudes and perceived barriers to partner notification for STIs
4. Uptake, yield and prevalence of STI in intervention clusters that offer STI testing.
5. Quantitative analysis of the association between acceptance of STI testing and predictive factors
6. Prevalence of STIs by study arm

p. 5.6 Pilot study of investigation into prevalence of exposure to trauma

i. 5.6.1 Background and description

Trauma exposure in youth as described in the Diagnostic and Statistical Manual 5 involves danger of death, injury, physician or sexual abuse, war, conflict and chronic disease that can predispose young people to diverse mental health problems specifically post-traumatic stress disorder (PTSD).[74] Research, mostly from high-income settings, has shown that post -traumatic stress disorder (PTSD), depression and substance use are common in young people who experience trauma.[75] The consequences of the mental disorders can be far reaching, including reduced quality of life, increased use of mental health services and impaired functioning.[76] There is also increasing evidence of a link between repeated traumatic exposures and effects on physical health. People living in LMICs like Zimbabwe likely have more exposure to “trauma” than those living in high-income settings including higher levels of interpersonal violence and socioeconomic stressors.[77,78] In addition, the recent COVID19 pandemic and the consequent control measures such as restricted movement and lockdowns may be experienced as a traumatic life-event.[79] The CHIEDZA project provides an opportunity to conduct a pilot to investigate if young people do report high levels of trauma. This pilot will inform a more in-depth investigation of the prevalence

of trauma, the types of trauma, its consequences and the appropriate interventions that should be developed and implemented.

A convenience sample of 100 individuals aged 18-24 years accessing the CHIEDZA intervention (in Harare province only) who provide written informed consent will be recruited to determine the traumatic events that young people go through using the Life events checklist (LEC). The measure is designed to screen for potentially traumatic events in a respondent's lifetime. It assesses exposure to 16 events that are associated with development of PTSD. The scale includes one additional item assessing any other extraordinarily stressful event not captured in the first 16 items. This will be complemented by qualitative interviews in approximately 30 participants who have experienced traumatic events to learn about the traumas experienced and their mental health consequences and their health seeking behaviour. The LEC and interviews will be administered by a trained clinical psychologist and anyone identified with PTSD will be referred for clinical care to Parirenyatwa Hospital, with management costs to be covered by the study.

- # 6. Data management

There are different forms and uses of data within the study.

q. *6.1 Process data for intervention delivery*

i. 6.1.1 Identification of service users

To make the intervention 'youth friendly', identifying information such as name and address will not be recorded for clients accessing the CHIEDZA intervention. Instead, biometrics (fingerprint recognition) will be used to record visits and the services received. At the first visit to the CHIEDZA service, demographic data (age, sex, date of birth and initials will be recorded and fingerprints collected. Clients will choose a memorable password which will be used to identify the correct match at subsequent visits. If biometric identification is not possible or clients decline to provide fingerprints, this will not be a reason to deny services. A backup registration process will be available, consisting of an ID number allocated to the client.

ii. 6.1.2 Monitoring service use

Individuals accessing the CHIEDZA service will be identified only by their fingerprints on each visit, except for certain services (see below). Fingerprint recognition operates by returning the most closely matching 3-5 records in its database. In the event that the password is forgotten, age, sex and initials will be used to verify the match. At each visit the client makes, the services accessed will be recorded (Figure 5). Where serious concerns are identified (red-flag), personal details will be collected to follow-up on concerns. The only information from previous visits available to a member of CHIEDZA service staff through the fingerprint match will consist of:

- HIV status
- Date of last HIV test
- Red flag

. For those who test HIV-positive, identifying data including name and address and contact/locator details will be collected. This will be done to register them into the national HIV programme and to follow-up those who do not attend for drug collection. Attendance at CAPS groups, and ART prescribed and dispensed will be recorded and transcribed into the national ART programme records.

Demographic data at registration and services accessed at visits will be recorded off-line using an Android based data capture application called Survey CTO Collect. SIMPRINTS biometric technology will be integrated with Survey CTO to track visits to the CHIEDZA and services accessed at each attendance. After the data are collected off-line, it will be synchronised over our local wi-fi network to the Survey CTO Server. Data will be quality controlled on a real-time basis using automated quality checks.

Access to the data will be limited to the Data Management personnel and specified study personnel. The data collection and entry processes will be as detailed in Standard Operating Procedures. Data will be quality controlled on a real-time basis using automated quality checks.

r. 6.2 Outcome data from cross-sectional survey

The cross-sectional survey will be conducted at community level by trained research assistants, using electronic tablets with pre-designed data entry forms including validation checks and skip patterns. Fingerprints will be collected from each survey participant. The research assistants will ask questions and fill in answers on the tablet. For sensitive questions, the participant will input the answers directly to prevent social desirability bias. Biometric records obtained from participants in the cross-sectional survey will be matched against all biometric records of clients accessing the intervention to assess degree of contamination and coverage of the intervention.

Figure 6: Different forms of data and linkage to fingerprints

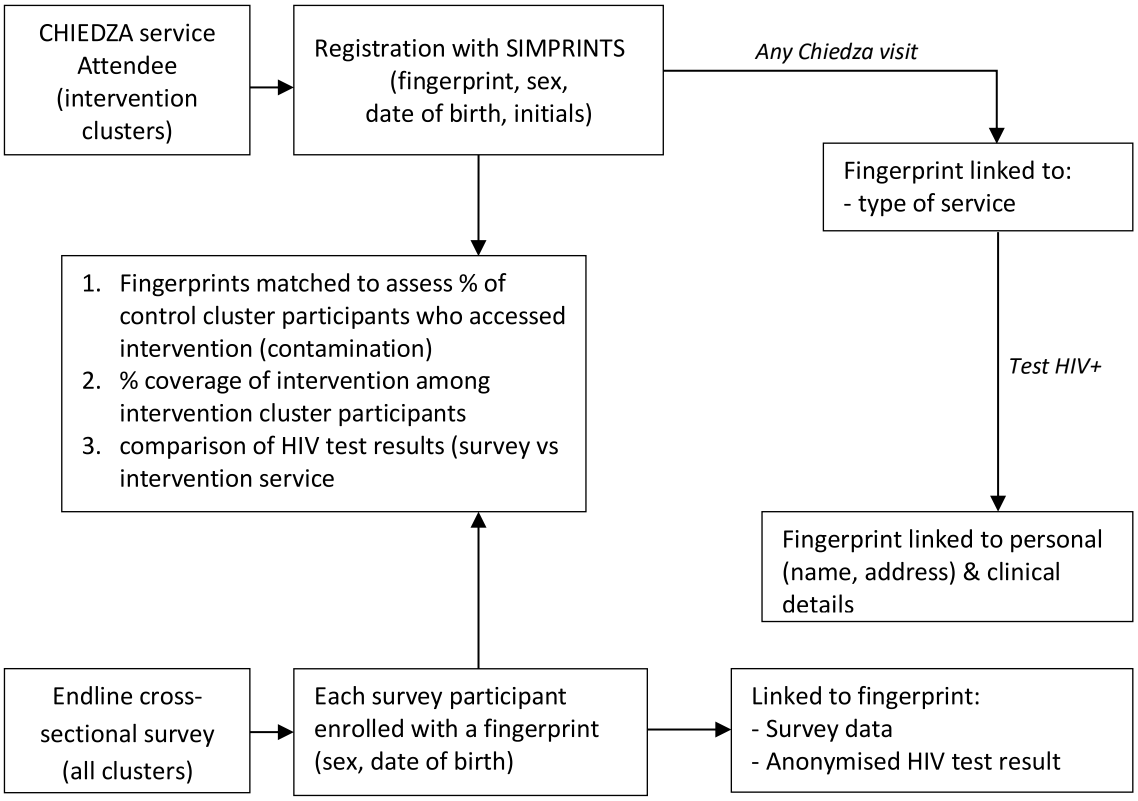

• 7. Statistical considerations and Data analysis

s. 7.1 Outcomes

All outcomes will be measured in the cross-sectional survey after 2 years of implementation.

The primary endpoint is the proportion of HIV positive participants in the survey who have virological suppression (viral load <1000 copies/ml), by arm.

The secondary outcomes are

- Proportion of HIV positive participants who are aware of their HIV status
- Proportion of those aware of their HIV positive status who are taking ART
- Proportion of those on ART who are virally suppressed

These outcomes correspond to the individual UNAIDS 90-90-90 targets, while the primary endpoint is the product of all three.

Other outcomes will be measured among all survey participants, regardless of HIV status. They are

- Proportion with knowledge of their HIV status
- Proportion with STIs
- Proportion of males circumcised
- Proportion of females using modern methods of contraception
- Proportion with good MHM (defined as using a clean menstrual management material to absorb or collect blood that can be changed in privacy as often as necessary for the duration of the menstruation period, using soap and water for washing the body as required, and having access to facilities to dispose of used menstrual management materials[80])
- Proportion with symptoms of common mental disorders (measured using the Shona Symptom Questionnaire-14 with a cutpoint of 9)
- Proportions who report tobacco, alcohol and substance use
- Sexual behavior (including no. of sexual partners in the past year and condom use)

ART drug resistance will be measured in samples that are virologically non-suppressed (subject to funding).

t. 7.2 Sample size calculations

For the primary outcome, we assume that

- the community prevalence of HIV in the population aged 18-24 (the age range of the cross-sectional survey) is conservatively estimated at 3.0%- based on a prevalence of 3.6% in 15-19 year olds and 5.5% in 20-24 year olds in the ZIMPHIA survey (a national population-based survey conducted in Zimbabwe between 2015-2016)[3]
- the coefficient of variation in HIV prevalence between clusters is 0.25-0.3

- The primary outcome is the proportion of young people living with HIV who are virally suppressed, expected to be 43% in the control arm (60% diagnosed x 70% linked to care x 63% virally suppressed).

The trial is aiming to meet 90-90-90 targets and has >90% power to detect a difference (73% in the intervention arm). However, this is an aspirational target and the study will have enough power to detect a smaller difference in the primary outcome. The study continues to have high power in the scenarios of lower HIV prevalence or increased variation in HIV prevalence between clusters. The study has not been powered to undertake stratified analysis by sex or assess any differences in impact between them.

Using the *clustersampsi* command in Stata gives the following projections:

Table 6: Sample size calculation for primary outcome

Cluster size	HIV prevalence	Number HIV+ per cluster	Expected outcome in control arm	Power	Coefficient of variation	Minimum outcome prevalence in intervention arm that will show effect
**1000**	3%	30	43%	80%	0.25	62%
**1000**	3%	30	43%	80%	0.3	66%
**1000**	3%	30	43%	90%	0.25	66%
**1000**	3%	30	43%	90%	0.3	70%
**700**	3%	21	43%	80%	0.25	64%
**700**	3%	21	43%	80%	0.3	67%
**700**	3%	21	43%	90%	0.25	67%
**700**	3%	21	43%	90%	0.3	71%

The secondary outcomes rely on self-report of HIV-positive status and 2) ART use. In the ZIMPHIA survey, 50% of 15-24 year olds reported being HIV-positive, 83.7% of these reported using ART and of these 85.4% were virally suppressed. The first step of the HIV care cascade (HIV testing) appears to be the main step that will drive the difference in the primary outcome and the trial is powered to detect a difference in this step, including if there are higher proportions of those who report being HIV positive and a smaller difference between the control and intervention arms. The trial will not be powered to detect a difference in the second and third steps of the HIV care cascade.

Table 7: Sample size calculations for secondary outcome: Awareness of HIV positive status

Control arm	Intervention arm	Number HIV+ per cluster	HIV prevalence	K	Power
60%	90%	21	3.0%	0.25	92%
60%	90%	21	3.0%	0.3	82%
60%	80%	21	3.0%	0.25	64%
60%	80%	21	3.0%	0.3	52%

Sampling will continue until the target sample size is reached in each cluster. For the STI outcomes, the sample size calculations are given below:

Table 8: Sample size calculations for STI outcomes

Cluster size	Clusters	Expected outcome in control arm	Power	Coefficient of variation	Maximum prevalence in intervention arm that will show an effect
**300**	16	17%	80%	0.25	11%
**300**	16	17%	80%	0.3	10%
**300**	16	17%	90%	0.25	10%
**300**	16	17%	90%	0.3	9%

u. 7.3 Statistical analysis

CONSORT guidelines for cluster randomised trials will be followed. A flowchart will be created showing the number of clusters eligible, the number allocated to each arm and the number of participants per cluster in the cross-sectional survey. Cluster-level analyses will be used to adjust for between-cluster variability, as recommended for trials with fewer than 15 clusters/arm. Descriptive analysis will be used to compare the cluster-level characteristics of the two arms, with adjustment for variables that are imbalanced between arms (avoiding variables likely to be affected by the intervention) and for stratum.

All outcomes are binary. For each outcome, the risk for each cluster will be calculated, and shown by strata and arm. The mean and SD of the log risk will be used to estimate the geometric mean and associated 95%CI for each arm of the study. Linear regression of the log mean risk on strata and arm will be used to estimate the risk ratio and 95%CI. The approximate variance for the mean risks will be obtained based on the residual mean square from a 2-way ANOVA on arm and strata. A 95% CI for this will be calculated from the variance using a t-statistic.

Pre-defined sensitivity analysis will include multiple imputation to adjust for non-participation in the survey. Participants in the intervention clusters are not necessarily expected to have received the intervention personally. As a form of exploratory analysis we will assess the difference in outcomes between intervention arm participants who accessed the CHIEDZA intervention (defined as having had at least one HIV test through the CHIEDZA service), compared to control arm participants.

• 8. Ethical Considerations

8.1 Collaborative partnerships

Community representatives have been involved in the design of the intervention and have engaged with the research team to shape the intervention. Formative research was conducted with several stakeholders including youth (HIV-infected and -uninfected), family members of youth, health care providers at health facilities, including those involved in HIV testing, clinical care, and counselling / outreach, CHWs, representatives or key informants from CBOs that are engaged in providing adherence support or related services and community members and other gatekeepers. The formative research is described in a separate protocol that was approved by local and international ethics committees (Protocol numbers as follows- MRCZ: MRCZ/A/2266; BRTI: AP144/2018; LSHTM: 14652).

The research teams will continue to actively engage with the study communities at various levels (e.g. with government structures, healthcare facilities at management and worker level, existing NGOs and CBOs providing community-based services and other stakeholder groups). As discussed in Section 4.3, an YAG has been established during the formative study and will provide guidance and feedback to the study team.

v. 8.2 Social value

As described in Section 1, the worldwide burden of HIV infection continues to grow, with SSA being particularly affected. Young people have disproportionately poor outcomes across the HIV care cascade. If this study is able to show that the intervention is effective and cost-effective, it could contribute to meeting the ambitious UNAIDS targets which have been laid out as a means to ending the HIV epidemic, and which are currently not met in youth. The multifaceted approach of the intervention, incorporating the whole HIV care cascade as well as provision of SRH information and commodities and general health counselling offers value to youth in the community whether they are men or women, already infected with HIV or uninfected, even if the intervention ultimately does not impact upon the VL.

w. 8.3 Scientific validity

Young people stand out as a group that have not benefitted from the numerous interventions to improve HIV outcomes, and studies consistently have shown that this group has lower levels of VL suppression, but also are less likely to have accessed HTC and linked to care if tested HIV-positive. This study will determine if providing community-based services that address each step of the cascade and that have been configured to try and improve access and coverage will impact on viral load among youth. Furthermore, the study addresses the broader issue of access of services by youth. It is well-recognised that young people face substantial barriers to accessing health services in facilities and health services in many countries give low priority to this age-group which has contributed to their poorer health gains compared with young children or adults over recent

decades.[81,82] Therefore if community-based provision of services does result in high uptake (particularly of sensitive services such as SRH and HIV services), this may provide a model for delivery of other health services to this age-group.
The study will be conducted according to the most rigorous standards of research and will provide an understanding about the process of implementing the intervention as well as the impact of such an intervention at community level. The study results will be shared throughout the study with national and international policy-makers to ensure that the findings are understood and that lessons from the study are implemented.

x. 8.4 Risk-benefit assessment

This community-based, cluster-randomized study can potentially incur risk of harm at both a community and an individual level. Likewise, study-related benefits may accrue at both an individual and a community level. The benefits and risks and how they will be addressed are discussed below.

i. 8.4.1 Benefits and risks at community level

If the intervention is effective, this will lead to increased level of viral suppression at population level and contribute to meeting the ambitious UNAIDS 90-90-90 targets to help end the HIV epidemic. This age-group has disproportionately poor outcomes compared to other age groups (e.g. 45% viral suppression in 15-24 year olds compared to 60% in older age groups).[3] The intervention will likely have an impact on HIV incidence (although the study is not powered to detect this) as well as improved SRH. If the trial shows that the intervention is highly effective and cost-effective, efforts will be made to ensure that the control communities are among the first to benefit from this intervention.

Any community-based research project may present risks to a community. Communities may feel disempowered by having a research agenda imposed on them or they may be placed at risk of stigmatization by the publication or dissemination of research results. Large community research projects may disrupt intra-community social structures and networks that may not have been identified by the research team. We will conduct a detailed process evaluation, which will measure such harms and also have systems for monitoring and addressing social harms (see below). Communities will not be named in any publication or dissemination of results of the study. There could be an additional burden placed on already overwhelmed routine services. The intervention includes provision of ART and other services at community level and this may mitigate against this risk. The study team will work closely with government agencies and will not entice staff away from them by offering differential salary packages. Communication will be maintained with study communities for the duration of the study and a well-developed exit strategy will be planned with the input of all stakeholders and community engagement structures to ensure that there is a seamless transition from study to routine health services at the end of the study.

ii. 8.4.2 Risks and benefits at individual level

There are several benefits at individual level. All youth accessing the CHIEDZA service regardless of HIV status will be able to access SRH services and general health counselling. HTC will be available for both CHIEDZA service users as well as to all endline survey participants with defined linkage to care pathways in both cases. Knowledge of personal HIV status is the entry point to accessing care, treatment, while HIV-uninfected individuals will be able to access HIV prevention services.
Those who are identified to have STIs through testing will be treated according to National guidelines. Those identified with HIV through the CHIEDZA intervention will be offered immediate ART initiation. Several trials have shown that early treatment of HIV-infected patients has clinical benefit,[5,7] and the WHO now recommends initiation of HIV treatment regardless of disease stage in all age-groups.[4] ART reduces the incidence of TB and other infections, as well as protecting the immune system and significantly slowing HIV clinical progression.[83] It also significantly reduces the risk of onward transmission to sexual partners, with a consequent decrease in the anxiety and psychological distress associated with HIV infection.[6]

The risks include psychological trauma from learning one's HIV test status, and possible social harm, stigmatization or IPV related to intended or unintended disclosure of HIV status, either within or beyond the household. A red flag system will be in place to identify these risks. To minimise this, post-test counselling and support will be provided by staff trained to provide counselling according to national guidelines. Couple counselling and testing of partners will also be provided. Referral to CBOs will be made for those suffering from domestic violence. The risks of starting ART for any HIV infected individual includes side effects, the development of drug resistance if treatment adherence is sub-optimal and consequent limitation of future treatment options. Treatment will be in accordance with national guidelines. Toxicity associated with ART or cotrimoxazole prophylaxis will be monitored and treatment modified if necessary. Adherence support through CAPS groups or individual support will be provided to mitigate against this risk, with follow-up of those who miss their drug collection visits. Individuals who access HIV care and treatment will have their personal details and sensitive information collected, some of which will be entered into a research database. Collection and storage of sensitive health information carries with it the risk of unwanted disclosure if there is a breach of data security. A stringent protocol for maintaining confidentiality will be put into place (see Section 8.6). All intervention team staff will be trained on GCP as well as ongoing training and supervision on delivery of the intervention services.

The ethical concerns associated with using mystery clients as part of the process evaluation include the potential deceptiveness of this methodology. Mystery clients have been widely used to investigate provider behaviour and quality of care, and the process evaluation will employ mystery client methodologies that have been used in previous similar studies. All intervention team members will be informed that mystery client may visit the CHIEDZA sites. Consent for the mystery client visits will not be sought to avoid behaviour bias, as has been

demonstrated by health workers when they know they are being watched. Human subjects' regulations allow consent waivers if obtaining consent would threaten validity of the study, there is no participant harm, and the activities involved are standard. This holds true for this study.

The main risks associated with the endline survey are similar to risks relevant to any population-based epidemiological HIV research and primarily involve risks to privacy and confidentiality. This is addressed in Section 8.6. There are additional minor risks for the survey participants including the taking of specimens, which may include pain or bruising when blood samples are taken. Also, there is the risk that some questions addressed to participants, e.g. relating to their sexual behaviour or HIV infection status, may result in discomfort or distress. Those who are identified with common mental disorders or substance use problems will be referred for further care. All survey staff will undergo training in GCP and there will be a strong emphasis in staff training and supervision on the importance of strict confidentiality of information as well as on supportive interviewing skills. Blood collection will be carried out by fully trained staff using appropriate sterile procedures.

Risk assessment

In summary, there are risks associated with this study for the individuals and communities involved. Notably, all the services provided are within national guidelines, with the exception of inviting individuals to a CAPS group straight after diagnosis rather than once stable on ART. However, the period when individuals are at highest risk of loss to follow up and require most support is after HIV diagnosis. Furthermore, if clients opt not to receive ART through CAPS groups they will have the option to receive ART through the service (but not via the CAPS groups) or to receive ART through clinics (standard of care). The risks outlined are no more than those encountered in everyday life, and as outlined above, the study will as far as possible address anticipated risks. Thus, we believe that the overall risk-benefit assessment for this study is favourable at both a community and an individual level.

8.5 Consent procedures

In a community-based, cluster-randomised trial such as this one, informed consent needs to take place at several levels ranging from approval from the government authorities, to so-called "community consent", and finally to individual consent. However, obtaining individual consent from every individual living in every community involved in this study would be unfeasible. As discussed earlier in Section 3.3.1 the intervention teams, while an integral part of this research will deliver a community health care package that is recognised as good practice and as such is not a research intervention.

iii. 8.5.1 Approval from Authorities

The project was discussed with and approved by the MoHCC before submission for funding. Subsequently, the project has been presented to the relevant technical teams at MoHCC. Additionally, during the planning process of the study,

approval will be sought from other authorities such as provincial and district authorities and local authorities (see section 4.3).

iv. 8.5.2 Community Consent

It is of the nature of a cluster-randomised trial that entire communities are assigned to one study arm or the other, and individual consent for community allocation is not possible. True 'Community Consent' is only possible if the "community has a legitimate political authority, e.g. a tribal council that has the authority to make binding decisions on behalf of its members."[84] If used inappropriately, the concept of 'community consent' may result in a false sense of security or mandate. Notably, the clusters in this trial are not administratively defined units or self-defining communities, but are defined geographically based on estimated population size and geographical boundaries to minimise contamination. We will seek approval for community participation from community-level stakeholders who will be defined through the community engagement process (see section 4.3) and who will include local leaders. Following agreement to participate, community representatives will take part in a randomisation ceremony at which the allocation of communities to study arms will be decided using a transparent and fair process.

v. 8.5.3 Individual Consent

Individual consent for intervention activities

Because the proposed activities are poised between an established public health intervention (HIV testing, HIV care according to national guidelines, provision of standard SRH services) and a public health research project (data collection and additional follow-up), the team will seek from the ethics committees an alteration of consent to verbal consent for participation in the CHIEDZA intervention. There is adequate prior research to show that delivery of HIV testing including HIV self-testing, provision of condoms and other SRH services and linkage to routine facility or community-based services by community health workers is safe and effective, such that its deployment in this study could be considered as implementation of a public health service, and therefore not requiring written research consent from each of the 30,000- 40,000 individuals targeted by the service. Individual consent for HIV testing and other interventions such as provision of condoms, STI testing and treatment and referral for VMMC or cervical screening will be obtained using standard procedures (verbal consent) as these interventions are considered part of the routine delivery of HIV prevention services and not specifically study related. Thus individuals who undergo these procedures in the study communities will not specifically be asked to participate in a research study but rather will be asked to consent to these activities as part of their health care. National guidelines stipulate that those aged 16 years and older can give independent consent to accessing HIV and SRH services.[85]

Importantly, there will be no names or addresses collected from clients accessing the CHIEDZA service (including SRH services, general counselling and HTC). Attendees will be registered and identified through a well-established biometrics

system (SIMPRINTS). This has an inbuilt consent on an electronic platform and therefore participants will only have to give fingerprints if they wish to do so. The SIMPRINTS system will create a unique number for each distinct client when s/he first accesses the service. The only other data that will be collected with the fingerprint will be sex and date of birth and the type of service accessed. Given the large number of clients anticipated to use the service it will not be possible to identify an individual through sex and date of birth. Thus use of biometric identification allows us to identify an individual without collecting additional personally identifiable information. Agreeing to provide a fingerprint will not be a condition for accessing services, and any individual declining to provide their fingerprint will still be able to access the services offered.

However, there are aspects of the intervention that are innovative and go beyond what would be considered an extension of government health services, and for which personal details such as name, address and other personal details will be collected. This includes enrolment of individuals identified as being HIV-positive who will be offered enrolment into CAPS groups. While these groups are modelled on CARGs which are being routinely implemented in Zimbabwe, the CARGs enrol individuals who are stable on ART. Those who test HIV-positive will be enrolled into CAPS groups following diagnosis. The rationale for this is that the period following diagnosis is the highest risk period for attrition and therefore the period when support is most required. Also, HIV-positive individuals will receive supportive SMS messages, which is not standard of care. Personal details will be collected to register HIV-positive individuals into the National ART programme even if delivery of ART will be at community level, to follow-up those who do come to collect their ART, and to send supportive SMS messages, so its primary use is to provide benefit to clients. During follow up, the CHIEDZA intervention team will have access to the personally-identified data for their HIV-positive clients because they need this information to find individuals and provide individualized care. Any HIV-positive client-linked data retrieved from clinics, will only be shared for *research* use after personally identifying information has been removed. Besides the intervention team (whose access to personally-identified information will be limited to the HIV-positive clients they are providing care to) only the study data manager will have access to personally-identified HIV-positive client data, which will be stored in an encrypted database. The encryption method and encryption key will only be accessible to designated data managers. The service will have written information leaflets with details about the project as a whole as well as the contact details of the MRCZ.

In summary, verbal consent will be requested based on the following considerations:

i. Intervention activities involve delivering community based health care (rather than primarily research activities) and involve minimal risk as the data will be de-identified
ii. Rights and welfare of individuals will not be adversely affected by a verbal consent process as data will be stored confidentially and shared only in de-identified form with the research teams

iii. Requiring full written research consent from all individuals that come into contact with the CHIEDZA intervention would make this project logistically unfeasible
iv. Pertinent written information about the project will be available at all intervention sites.

Individual consent for research studies in all arms

Consent for the endline survey will be collected using electronic consent forms on tablets with the participants signing on the tablet. Participants will be shown a video which will provide details of the survey including the purpose, the research institutions conducting the study and the procedures to be undertaken. Specifically, the video will also detail how confidentiality will be maintained and emphasize that participation is voluntary. The video will show how the blood and urine sample (for those randomised to be included into the STI component of the survey) will be collected and how height, weight and blood pressure will be measured. The electronic consent form will specifically ask participants to confirm that they have watched the complete video, had all their questions answered and consent to participate. A paper version of the consent form (an exact copy of the electronic consent form) will also be signed by the participants. The paper form will list the URL of the website through which the video can be accessed in the future. This ensures that the information received by the participant (in the form of a video) can be linked to the participant signature. The consent form which the participant will keep will also have the contact details of the study team and of the ethics body (MRCZ) in case participants wish to make a complaint or raise an issue about the study or decide to withdraw in the future. To ensure GCP compliance for the electronic consent process, the ID number allocated for the survey participant will be recorded on the paper and the electronic consent form. This will ensure that the electronic consent and the paper consent form can be linked. Backup paper information sheets and consent forms will be available in case of failure of the electronic tablets.

The video that will serve to provide information to eligible participants will be co-designed with the study team and youth. A script will be used as a starting point for developing the video which will be iteratively refined in consultation with youth, fieldworkers and the research team. In addition, as well as a pilot of the video, a randomised trial will be embedded within the endline survey to check that the e-consent process is not inferior to the standard consent process.

Electronic consent forms has been used in other surveys including the ZIMPHIA 2015-2016 survey.[3]

This approach has been developed using international guidance available at:
https://www.fda.gov/regulatory-information/search-fda-guidance-documents/part-11-electronic-records-electronic-signatures-scope-and-application

https://www.hra.nhs.uk/about-us/news-updates/hra-and-mhra-publish-jointstatement-seeking-and-documenting-consent-using-electronic-methods-econsent/

Written informed consent will also be required of individuals participating in qualitative research activities that involve collection of participant-identified responses to interviewer questions such as IDIs or FGDs (see Section 5.2). However, written consent will not be sought for other types of qualitative methods, such as observation of persons who are not participants or for telephonic interviews, which might be necessary in some instances like COVID-19 prevention and infection control. Written informed consent will be required to participate in the STI and MHM sub-studies (see Section 5.4 and 5.5). Separate consent to store leftover urine samples collected for STI screening (for future studies) will also be sought (Section 3.2.4). To advance infection and prevention control in the COVID-era, telephonic interviews may become a norm for IDIs (section 5.2). Where possible, every effort will be made to obtain written consent, that can be followed up with a phone-based IDIs. However, when not possible, phone-based consent will be sought. Measures to verify true client identity will include 1) video-conference calls where possible with participants 2) asking client name, age & where they stay in the CHIEDZA communities

The study team has considerable experience of designing and implementing suitable models of informed consent for young people in Zimbabwe that may have low levels of functional literacy. Care will be taken to ensure that information materials are developed that are appropriate to the study population, with translation into Shona / Ndebele where necessary and back-translation into English to ensure accuracy. Research staff will go through the information with participants and questions will be asked to check their understanding of key points before signed consent is sought.

Waiver of parental consent for participants aged below 18 years

Zimbabwean guidelines stipulate that those aged under 18 years should have parental consent. For the MHM study and the process evaluation of the CHIEDZA intervention, a waiver of the need for consent from a guardian for those aged 16-17 years will be requested from the MRCZ. This is a "grey" area and has been subject to much controversy and therefore advice was sought from researchers with expertise of working with adolescents in Zimbabwe and within Southern Africa. Reference was also made to available African guidelines for studies on SRH and HIV in adolescents, including guidelines from the WHO and guidelines (the most recent one is published by the Government of Kenya in 2015 following a consultation with experts and stakeholders globally.[86,87]

The consensus is that a waiver for consent is acceptable if the following conditions are met:

<u>Research cannot be practically or adequately conducted without a waiver:</u> The main concern in this study is that participants will not be able to be open about their concerns if they worry that their guardians will ask questions and will sanction them for being open. The subject area around SRH including MHM and accessing SRH/HIV services is taboo and young people may be sanctioned by their guardians if they participate in such discussions, and therefore the responses obtained are often superficial and do not reflect the real concerns that

adolescents may have. Experts working in the area have found that the very participants whose guardians are likely to refuse consent for them to participate are the very ones who face the most problems accessing help for SRH problems. In practice, participants will be those who are accessing SRH, HIV and MHM services without requiring consent from guardians, and it is therefore reasonable that participants should be able to talk about their experience of accessing these services independently without guardian consent.

Risks are minimal: The study aims to understand the problems young women face with MHM and the effectiveness and acceptability of MHM. For the process evaluation, participants will be mainly questioned about their experience of accessing the CHIEDZA service and there are minimal risks associated with participation in these studies. The FGDs and questions will be age-appropriate and tailored to the needs of the age-group.

Rights and welfare not affected: It will made clear from the outset that participants can leave and stop whenever they want and that they will be under no compulsion to take part. Study staff will be trained in GCP and ethical conduct of research and will be supervised by the project coordinator who has more than 20 years of conducting field research in Zimbabwe. Procedures will be in place to enable participants to seek assistance on a confidential basis during and after taking part in the study on issues that they may need extra assistance or information for.

Protection of privacy and confidentiality: While this is a critical principle of ethical conduct of research generally, it is of particular importance in this case. Interviews will take place in a private location that participants feel comfortable in, and participants will be able to self-complete questionnaires. Participants will be reassured that their information will be held confidentially.

vi. 8.5.4 Consent to Access routine healthcare surveillance data

Waiver of individual consent for collecting routine healthcare surveillance data, particularly from the control communities will be sought from the ethics committees. Permission will be sought from the respective public health authorities to collect these data without specific individual informed consent. This information will be collected, coded, stored and managed in such a way as to ensure individual identity and privacy are protected at all times.

Table 9 summarise the types of data to be collected and the consent procedures.

Table 9: Types of data to be collected and consent

Time-point	Data collected	Consent procedure
	Intervention	
Registration into CHIEDZA service	Fingerprint, sex, date of birth	Fingerprint: Electronic built into SIMPRINTS platform<br>Sex and date of birth: Verbal

CHIEDZA service attendance	Type of service used linked to fingerprint	None
HIV self-testing	Telephone number and if using ITHAKA platform: HIV test kit barcode linked to fingerprint, sex, age, self-reported HIV test results	Verbal consent / electronic inbuilt consent if web-based platform (ITHAKA) used by client
Diagnosis of HIV	Name, contact details, socio-demographic and clinical data	Verbal consent
	Outcomes and Evaluation	
End-line cross-sectional survey	Socio-demographic data, location data, contact details, trial outcomes including HIV test and VL	"Written" informed consent- administered on paper or on tablets
FGD/IDI during process evaluation of intervention	Sociodemographic data, audio-taped interviews	Written informed consent
MHM cohort study	Sociodemographic data, contact details, study data	Written informed consent
	Routine healthcare surveillance	
Throughout intervention	Aggregate rates of STI, HIV diagnoses, contraception use, VMMC in healthcare facilities by arm	Waiver of consent

y. 8.6 Confidentiality

Strict measures will be in place to safeguard confidentiality of data. All laboratory specimens, reports, study data collection, process, and administrative forms will be identified by coded numbers only to maintain participant confidentiality.

For clients accessing the CHIEDZA intervention, the registration will involve a collection of a fingerprint with in-built electronic platform consent for providing the fingerprint) as the only feasibly identifying information. Date of birth and sex will also be collected but it will not be feasible to identify individuals given the large numbers of individuals using date of birth and sex. The fingerprint data will be converted into a unique ID number and no other personal data (e.g. names/ addresses) will be collected. The data will be used as to track the number of attendances and uptake of services.

Additional identifying information (names and contact details) will be collected in the following groups:

1. Those who opt to access HIV care and receive ART through the HIV programme: personal data will be collected for 1) operational purposes (i.e. to register them in the national HIV Programme) and 2) for clinical care (to

ensure that those who do not attend to collect their drugs are followed-up and/or receive SMS as part of the adherence support).
2. MHM substudy, qualitative study and end-line survey participants for operational and logistic purposes (e.g. to make appointments to visit them at home to conduct the survey or referral to services). See Section 9.1 for description of how confidentiality will be maintained at the level of the laboratory.

Personal identifiers will appear on paper or electronically on consent forms, log books, follow up lists and other listings (as specified above). These listings will NOT include any (sensitive) study information (including laboratory data). A unique study number will be used to link personal identifiers to study information. Personal identifiers on paper will be stored in a locked cabinet. Electronically kept personal identifiers will be stored in separate datasets with password protection only accessible for designated staff (for computers and servers). Hand-held devices will also be password protected and personal identifiers will be stored in an encrypted format. Any data transfer over wireless or mobile networks will use Virtual Private Networks or router protected dedicated internet protocol addresses. Participants' study information will not be released without the written permission of the participant, except as necessary for trial monitoring (as discussed below), and/or government and regulatory authorities including Ethics committees and IRBs. Datasets will be stripped of any personal identifier before analysis. Survey CTO and SIMPRINTS adhere to GDPR. SIMPRINTS is regulated by the UK Information Commissioner's Office, the UK's independent regulatory body for privacy and data protection. Prior to processing data for the CHIEDZA study, SIMPRINTS will complete a data protection impact assessment to identify any potential risks and describe risk mitigation procedures. SIMPRINTS also employs best-practice standards in data security and encrypts data at all stages of its processing operations, including collection, transfer and storage. Importantly, biometrics (together with the pseudonymised identifier, non-personal information and GPS coordinates) and the programme information collected via Survey CTO will be stored in separate databases. This increases the protection of client data in case of a breach into either database.

All electronic data will be stored in password protected database systems, with access granted to authorised staff only. All computers, servers and networks on which the data are stored will be password protected. All collected study data on central computers and servers, remote computers and hand-held devices, will be backed up daily. Backup tapes/discs will be stored separately from the primary electronic storage.

FGDs and IDIs will be recorded digitally, and then deleted from recorders once downloaded onto password-protected project computers. Audio files will be kept for five years, and identifiable only by interviewee code. All FGDs and interviews will be transcribed and translated, but will include no individual identifiers or identifying attributes. Verbatim quotes may be included in reports or publications, but will only identify the category of participant, their sex and age.

z. 8.7 Data and Safety monitoring

A DSMB will not be established as the outcome will be determined through a cross-sectional survey at the end of the intervention. A TSC will be established which will include a mix of senior trial investigators including the PI and external members, with an independent chair. The TSC will review operational performance including uptake, retention and adverse events on an ongoing basis, and will advise the study team on trial conduct. Data on the uptake of each component of the trial intervention will be captured electronically in all intervention communities. We will monitor intervention uptake on a monthly basis. This will be used to inform adaptations to improve uptake of and access to the intervention.

Adverse events, defined as an untoward medical occurrence in an individual to whom a medicinal product has been administered including occurrences that are not necessarily caused by or related to that product, among individuals accessing the intervention will be recorded. The products referred to include ART, STI treatment, contraception and MHM products.

Social harms are defined as any untoward social occurrences that happen to a participant as a result of their participation in the study, with examples including loss of employment, harassment by neighbours, shunned by family, rejection by partner etc. Because this study is a community-randomized trial of a multi-faceted intervention, the majority of people in the community affected by the implementation of the study will not be participants in the evaluation surveys, and so the definition of social harm for this study will be expanded to also include any untoward social occurrences that happen to a community, or groups or individuals within a community, as a result of implementation of the study intervention. Social harms will be monitored throughout the study (through report from those accessing the CHIEDZA intervention) and study staff will be trained to report these on a regular basis and to provide appropriate counselling and/or referral to appropriate resources, as needed. Staff may also become aware of social harms passively e.g. if they live in the intervention communities, and will be encouraged to report these.
Discussion of social harms will be addressed with the TSC and with the YAG. The qualitative research to be conducted as part of the process evaluation will also explore social harms in the community. The study team will review the social harms reports monthly, or sooner, if a concerning trend or event is identified. If the team judges an individual social harm, or a trend in social harms, to be serious or unexpected, they will work together with appropriate bodies (IRBs, YAG, TSC, Trial sponsor) to determine if a response is indicated, and if so, what it should be.

Adverse events and social harms will be reported to the ethics committees according to their individual requirements.

aa. 8.8 Clinical management during and post-study

HIV treatment in the intervention arm will be provided according to national guidelines which stipulate that all individuals who test HIV-positive are eligible

for ART regardless of age or disease stage.[8] ART will be obtained through the national health systems and documentation of individuals will be done in line with existing procedures in the public sector. Procedures for ensuring that individuals with HIV starting treatment are registered within the national system and referral criteria and pathways for those requiring health-facility-based care will be established (e.g. those who have incident symptoms or side-effects). Other clinical management e.g. treatment for STIs and provision of contraception will also follow national guidelines.

CAPS groups will be trained and supported to become self-sustaining over the intervention period, so that following the intervention period the groups will be maintained. Individuals accessing ART through the CHIEDZA intervention will be made aware that following the end of the intervention period they will have the option of continuing to be part of a CAPS group (should the group agree) or to access ART from their nearest PHC.

bb. 8.9 Ethical Review

Approval to conduct this study will be obtained from the MRCZ, the BRTI IRB and the LSHTM Ethics Committee. Where there is disagreement or discordant IRB requirements the condition providing the highest level of human subject protection will be implemented. Adverse events will be reported on a regular basis according to the individual requirements of these IRBs, and annual reports will be sent to ethics committees according to the requirement of each committee.

- # 9. Laboratory Procedures

Testing of specimens will be performed at accredited laboratories that will adhere to GCLP and with quality management systems in place, with regular external audit of laboratory procedures. All activities related to processing, labelling of samples, testing, storage and shipment (if required) will follow Standard Operating Procedures.

cc. 9.1 Cross-sectional survey tests

Participants will be asked to provide a blood sample for anonymised HIV testing (a dried blood spot specimen). The samples will be assayed in batches for HIV antibodies using an ELISA 4th generation HIV antibody assay. All positive tests will be confirmed with a separate ELISA assay (assay to be determined) on the same sample. One in twenty samples that test HIV negative will also be re-tested to provide Quality Assurance. All HIV-positive samples will be tested for HIV VL. The assay to be used will depend on local availability. In addition to the tests described above, study participants will be asked to provide written informed consent for their specimens to be stored for possible additional, future testing (long-term storage). The specimens of participants who do not consent to long-term storage for future research will be destroyed after all protocol-related testing has been completed. Specimens will only bear the study ID of the participants. The study ID number will be put on each specimen for anonymised testing.

All participants will be provided with information about HIV testing and counselling and where this can be accessed. Those who wish to know their HIV status will be referred to the local health facility for expedited diagnostic HIV testing. Home-based testing is not being offered based on previously low levels of uptake due to concerns by youth of unintended disclosure and lack of complete privacy when HIV testing is performed at home.

Participants who are randomised for STI testing and have a positive STI test result will be contacted (using a prior agreed pseudonym) and will be treated by the study team and partner notification using client referral undertaken.

dd. 9.2 Safety issues

As the transmission of HIV and other blood-borne pathogens can occur through contact with contaminated needles, blood, and blood products, appropriate blood and secretion precautions will be employed by all personnel in the drawing of blood and shipping and handling of all specimens for this study. Staff training will be given to all employees given regarding universal precautions and avoidance of needle-stick injuries. A policy for needle-stick injuries is in place. All staff will be vaccinated against Hepatitis B and encouraged to have COVID19 vaccination. Residual specimens, used specimen containers and laboratory waste will be placed in sealed autoclaved bags, autoclaved, and incinerated at the end of each working day. COVID19 Infection prevention and control procedures will be in place including use of masks, hand sanitisers and social distancing practised by staff, clients and participants.

• 10. Administrative Procedures

ee. 10.1 Regulatory approvals

The trial will be conducted in accordance with the principles of the Declaration of Helsinki and in full conformity with relevant regulations and with the ICH Guidelines for Good Clinical Practice (CPMP/ICH/135/95) June 1996. Prior to implementation of this protocol, and any subsequent full version amendments, the protocol and the consent form(s) will be approved by the MRCZ, the LSHTM Ethics Committee and the BRTI IRB. The PI will register as a foreign researcher with the RCZ.

ff. 10.2 Trial registration

The trial has been registered with Clinicaltrials.gov Registry (**https://clinicaltrials.gov/**): Trial number NCT 03719521

gg. 10.3 Indemnity

LSHTM holds Public Liability ("negligent harm") and Clinical Trial ("non-negligent harm") insurance policies which apply to this trial.

hh. 10.4 Study monitoring

The MRCZ will conduct regulatory inspections to ensure that study procedures are compliant with GCP and will assess adherence to protocol and to SOPs, and confirm the quality and accuracy of information being collected. The study may also be subject to an audit by the RGIO office at LSHTM under its remit as the Trial Sponsor. External trial monitoring will be carried out by the Research Support Centre at the University of Zimbabwe. The study team will allow the study monitors to inspect study facilities and documentation, as well as to observe the performance of study procedures. An independent TSC will oversee trial progress, regularly review relevant information from other sources (e.g. other related trials) and advise the study team.

ii. 10.5 Protocol compliance

The study will be conducted in full compliance with the protocol. The protocol will not be amended without prior written approval by the PI. All protocol amendments will be submitted to and approved by the MRCZ, the LSHTM Ethics Committee and to the BRTI IRB prior to implementing the amendment. At the end of the study the MRCZ will be notified of the completion of the study (using the FORM 105- study termination form) and a final study report will be sent to the MRCZ.

jj. 10.6 Maintenance of records

The study team will maintain and securely store complete, accurate and current records throughout the study. All study records will be maintained for five years after the end of the study, as per MRCZ regulations. Study records include

protocol registration documents, all reports and correspondence relating to the study and documentation related to study participants (including informed consent forms, locator forms, case report forms, notations of all contacts with the participant, and all other source documents).

kk.

ll. 10.7 Use of data and publications

All processes related to data use will follow the principles of Good Clinical Practice. At the time of publication of research the subset of data required for the purposes of verifying research findings will be made available for sharing, and will be placed in the institutional research data repository established by the LSHTM Research Data Management Support Service. This repository will enable direct download of records with codebooks to enable replication of the data analyses. A more complete sharing of data with any research group requesting access to individual data records will be done 12 months after completion of the study. At this point, all data and study tools will be made available through the LSHTM Institutional Research Data Repository. Data for sharing will be anonymised prior to release for data sharing. In addition, annotated questionnaires and STATA do-files used for data cleaning and analysis will be available. All databases will be password-protected and accessible to authorised personnel only. The LSHTM open access repository will also enable access to repository contents through a searchable index. Where individual data are concerned, the informed consent procedure will clarify the possibility of use of anonymised data by other researchers. Data users will be required to acknowledge the source of data and to ensure that the regulatory requirements of the MRCZ and other ethical bodies reviewing the projects are met.

Publication of the results of this study will be governed by the requirements of the Wellcome Trust open access policy. All publications will be made available through PubMed Central (PMC) and Europe PMC as soon as possible and within six months of the journal publisher's official date of final publication. Where possible (and when an open access fee is paid), research papers will be licenced a Creative Commons Attribution licence (CC-BY).

• 11. References

1. UNAIDS Data 2017. Geneva, Switzerland: Joint United Nations Programme on HIV/AIDS 2017.
2. Miles To Go. Global AIDS Update 2018. . Geneva, Switzerland: UNAIDS, 2018.
3. Zimbabwe Population-based HIV Impact Assessment (ZIMPHIA) 2015-16: First Report. Harare, Zimbabwe: Ministry of Health and Child Care (MOHCC) Zimbabwe, 2017.
4. Consolidated guidelines on the use of antiretroviral drugs for treating and preventing HIV infection: Recommendations for a public health approach (second edition). Geneva, Switzerland: World Health Organization, 2016.
5. Lundgren JD, Babiker AG, Gordin F, et al. Initiation of Antiretroviral Therapy in Early Asymptomatic HIV Infection. *N Engl J Med* 2015; **373**(9): 795-807.
6. Cohen MS, Chen YQ, McCauley M, et al. Prevention of HIV-1 infection with early antiretroviral therapy. *N Engl J Med* 2011; **365**(6): 493-505.
7. Danel C, Moh R, Gabillard D, et al. A Trial of Early Antiretrovirals and Isoniazid Preventive Therapy in Africa. *N Engl J Med* 2015; **373**(9): 808-22.
8. Guidelines for Antiretroviral Therapy for the Prevention and Treatment of HIV in Zimbabwe. Zimbabwe: National Medicines and Therapeutics Policy Advisory Committee (NMTPAC) and The AIDS and TB Directorate, Ministry of Health and Child Care, Zimbabwe, 2016.
9. Govindasamy D, Ford N, Kranzer K. Risk factors, barriers and facilitators for linkage to antiretroviral therapy care: a systematic review. *Aids* 2012; **26**(16): 2059-67.
10. Katz IT, Kaplan R, Fitzmaurice G, et al. Treatment guidelines and early loss from care for people living with HIV in Cape Town, South Africa: A retrospective cohort study. *PLoS Med* 2017; **14**(11): e1002434.
11. De Cock KM, Gilks CF, Lo YR, Guerma T. Can antiretroviral therapy eliminate HIV transmission? *Lancet* 2009; **373**(9657): 7-9.
12. Definition of youth. Fact sheet.: United Nations Department of Economic and Social Affairs, 2016.
13. Bygrave H, Mtangirwa J, Ncube K, Ford N, Kranzer K, Munyaradzi D. Antiretroviral Therapy Outcomes among Adolescents and Youth in Rural Zimbabwe. *PLoS ONE* 2012; **7**(12): e52856.
14. Evans D, Menezes C, Mahomed K, et al. Treatment Outcomes of HIV-Infected Adolescents Attending Public-Sector HIV Clinics Across Gauteng and Mpumalanga, South Africa. *AIDS Res Hum Retroviruses* 2013.
15. UNAIDS. 90-90-90. An ambitious treatment target to help end the AIDS epidemic. Geneva, Switzerland, 2014.
16. Kapogiannis BG, Legins KE, Chandan U, Lee S. Evidence-based programming for adolescent HIV prevention and care: operational research to inform best practices. *J Acquir Immune Defic Syndr* 2014; **66 Suppl 2**: S228-35.
17. Kranzer K, Meghji J, Bandason T, et al. Barriers to provider-initiated testing and counselling for children in a high HIV prevalence setting: a mixed methods study. *PLoS Med* 2014; **11**(5): e1001649.
18. Denno DM, Hoopes AJ, Chandra-Mouli V. Effective strategies to provide adolescent sexual and reproductive health services and to increase demand and community support. *J Adolesc Health* 2015; **56**(1 Suppl): S22-41.
19. Suthar AB, Ford N, Bachanas PJ, et al. Towards universal voluntary HIV testing and counselling: a systematic review and meta-analysis of community-based approaches. *PLoS Med* 2013; **10**(8): e1001496.
20. Sweat M, Morin S, Celentano D, et al. Community-based intervention to increase HIV testing and case detection in people aged 16-32 years in Tanzania, Zimbabwe, and Thailand (NIMH Project Accept, HPTN 043): a randomised study. *Lancet Infect Dis* 2011; **11**(7): 525-32.

21. Kwame S, Ab S, Joseph CM, et al. Community intervention improves knowledge of HIV status of adolescents in Zambia: findings from HPTN 071-PopART for Youth Study. *AIDS (London, England)* 2017; **31**(Suppl 3): S221-S32.
22. Choko AT, Desmond N, Webb EL, et al. The uptake and accuracy of oral kits for HIV self-testing in high HIV prevalence setting: a cross-sectional feasibility study in Blantyre, Malawi. *PLoS Med* 2011; **8**(10): e1001102.
23. Coates TJ, Kulich M, Celentano DD, et al. Effect of community-based voluntary counselling and testing on HIV incidence and social and behavioural outcomes (NIMH Project Accept; HPTN 043): a cluster-randomised trial. *The Lancet Global health* 2014; **2**(5): e267-77.
24. MacPherson P, Lalloo DG, Webb EL, et al. Effect of optional home initiation of HIV care following HIV self-testing on antiretroviral therapy initiation among adults in Malawi: a randomized clinical trial. *JAMA* 2014; **312**(4): 372-9.
25. Barnabas RV, van Rooyen H, Tumwesigye E, et al. Initiation of antiretroviral therapy and viral suppression after home HIV testing and counselling in KwaZulu-Natal, South Africa, and Mbarara district, Uganda: a prospective, observational intervention study. *The lancet HIV* 2014; **1**(2): e68-e76.
26. Asiimwe S, Ross JM, Arinaitwe A, et al. Expanding HIV testing and linkage to care in southwestern Uganda with community health extension workers. *Journal of the International AIDS Society* 2017; **20**(Suppl 4): 21633.
27. Bemelmans M, Baert S, Goemaere E, et al. Community-supported models of care for people on HIV treatment in sub-Saharan Africa. *Trop Med Int Health* 2014; **19**(8): 968-77.
28. Grimsrud A, Barnabas RV, Ehrenkranz P, Ford N. Evidence for scale up: the differentiated care research agenda. *J Int AIDS Soc* 2017; **20**(Suppl 4): 22024.
29. Ammon N, Mason S, Corkery JM. Factors impacting antiretroviral therapy adherence among human immunodeficiency virus-positive adolescents in Sub-Saharan Africa: a systematic review. *Public health* 2018; **157**: 20-31.
30. Ridgeway K, Dulli LS, Murray KR, et al. Interventions to improve antiretroviral therapy adherence among adolescents in low- and middle-income countries: A systematic review of the literature. *PLoS One* 2018; **13**(1): e0189770.
31. MacPherson P, Munthali C, Ferguson J, et al. Service delivery interventions to improve adolescents' linkage, retention and adherence to antiretroviral therapy and HIV care. *Trop Med Int Health* 2015; **20**(8): 1015-32.
32. Differentiated Care For HIV and Tuberculosis. Geneva, Switzerland: The Global Fund, 2015.
33. HIV care and support: HIV care and support taking into account the 2016 WHO consolidated guidelines. Geneva, Switzerland: UNAIDS, 2016.
34. Lamb MR, Fayorsey R, Nuwagaba-Birbonwoha H, et al. High attrition before and after ART initiation among youth (15-24 years of age) enrolled in HIV care. *AIDS* 2013.
35. Kwarisiima D, Kamya MR, Owaraganise A, et al. High rates of viral suppression in adults and children with high CD4+ counts using a streamlined ART delivery model in the SEARCH trial in rural Uganda and Kenya. *J Int AIDS Soc* 2017; **20**(Suppl 4): 21673.
36. Nachega JB, Adetokunboh O, Uthman OA, et al. Community-Based Interventions to Improve and Sustain Antiretroviral Therapy Adherence, Retention in HIV Care and Clinical Outcomes in Low- and Middle-Income Countries for Achieving the UNAIDS 90-90-90 Targets. *Curr HIV/AIDS Rep* 2016.
37. Chibanda D, Verhey R, Munetsi E, Rusakaniko S, Cowan F, Lund C. Scaling up interventions for depression in sub-Saharan Africa: lessons from Zimbabwe. *Global Mental Health* 2016; **3**: 1-9.
38. Gore FM, Bloem PJ, Patton GC, et al. Global burden of disease in young people aged 10-24 years: a systematic analysis. *Lancet* 2011; **377**(9783): 2093-102.

39. Moore G, Audrey S, Barker M, et al. Process evaluation of complex interventions: UK Medical Research Council (MRC) guidance. United Kingdom: Medical Research Council, 2014.
40. UNAIDS. Manual for costing HIV facilities and services. Geneva, Switzerland, 2011.
41. Rosen S, Maskew M, Fox MP, et al. Initiating Antiretroviral Therapy for HIV at a Patient's First Clinic Visit: The RapIT Randomized Controlled Trial. *PLoS Med* 2016; **13**(5): e1002015.
42. Delany-Moretlwe S, Cowan FM, Busza J, Bolton-Moore C, Kelley K, Fairlie L. Providing comprehensive health services for young key populations: needs, barriers and gaps. *Journal of the International AIDS Society* 2015; **18**(2Suppl 1): 19833.
43. Cohen DA, Farley TA. Social marketing of condoms is great, but we need more free condoms. *Lancet* 2004; **364**(9428): 13-4.
44. Robinson MN, Tansil KA, Elder RW, et al. Mass media health communication campaigns combined with health-related product distribution: a community guide systematic review. *Am J Prev Med* 2014; **47**(3): 360-71.
45. Barnabas RV, van Rooyen H, Tumwesigye E, et al. Uptake of antiretroviral therapy and male circumcision after community-based HIV testing and strategies for linkage to care versus standard clinic referral: a multisite, open-label, randomised controlled trial in South Africa and Uganda. *The lancet HIV* 2016; **3**(5): e212-20.
46. Garrett NJ, Osman F, Maharaj B, et al. Beyond syndromic management: Opportunities for diagnosis-based treatment of sexually transmitted infections in low- and middle-income countries. *PLoS One* 2018; **13**(4): e0196209.
47. Barnabas SL, Dabee S, Passmore JS, et al. Converging epidemics of sexually transmitted infections and bacterial vaginosis in southern African female adolescents at risk of HIV. *Int J STD AIDS* 2018; **29**(6): 531-9.
48. Gaydos CA, Van Der Pol B, Jett-Goheen M, et al. Performance of the Cepheid CT/NG Xpert Rapid PCR Test for Detection of Chlamydia trachomatis and Neisseria gonorrhoeae. *J Clin Microbiol* 2013; **51**(6): 1666-72.
49. Garrett N, Mitchev N, Osman F, et al. Diagnostic accuracy of the Xpert CT/NG and OSOM Trichomonas Rapid assays for point-of-care STI testing among young women in South Africa: a cross-sectional study. *BMJ open* 2019; **9**(2): e026888.
50. Cousminer DL, Mitchell JA, Chesi A, et al. Genetically Determined Later Puberty Impacts Lowered Bone Mineral Density in Childhood and Adulthood. *J Bone Miner Res* 2018; **33**(3): 430-6.
51. Fonner VA, Dalglish SL, Kennedy CE, et al. Effectiveness and safety of oral HIV preexposure prophylaxis for all populations. *AIDS* 2016; **30**(12): 1973-83.
52. Pearson AL, Rzotkiewicz A, Zwickle A. Using remote, spatial techniques to select a random household sample in a dispersed, semi-nomadic pastoral community: utility for a longitudinal health and demographic surveillance system. *International journal of health geographics* 2015; **14**: 33.
53. Patel V, Simunyu E, Gwanzura F, Lewis G, Mann A. The Shona Symptom Questionnaire: the development of an indigenous measure of common mental disorders in Harare. *Acta Psychiatr Scand* 1997; **95**(6): 469-75.
54. Maheswaran H, Clarke A, MacPherson P, et al. Cost-Effectiveness of Community-based Human Immunodeficiency Virus Self-Testing in Blantyre, Malawi. *Clin Infect Dis* 2018; **66**(8): 1211-21.
55. Madziyire MG, Magure TM, Madziwa CF. Menstrual Cups as a Menstrual Management Method for Low Socioeconomic Status Women and Girls in Zimbabwe: A Pilot Study. *Women's Reproductive Health* 2018; **5**(1): 59-65.
56. Juma J, Nyothach E, Laserson KF, et al. Examining the safety of menstrual cups among rural primary school girls in western Kenya: observational studies nested in a randomised controlled feasibility study. *BMJ open* 2017; **7**(4): e015429.

57. Hennegan J, Dolan C, Wu M, Scott L, Montgomery P. Measuring the prevalence and impact of poor menstrual hygiene management: a quantitative survey of schoolgirls in rural Uganda. *BMJ open* 2016; **6**(12).
58. Sekhon M, Cartwright M, J. Francis J. Acceptability of healthcare interventions: An overview of reviews and development of a theoretical framework. *BMC Health Services Research* 2017; **17**.
59. Cowan FM, Chabata ST, Musemburi S, et al. Strengthening the scale-up and uptake of effective interventions for sex workers for population impact in Zimbabwe. *Journal of the International AIDS Society* 2019; **22**(S4): e25320.
60. Global health sector strategy on Sexually Transmitted Infections, 2016-2021 Geneva, Switzerland: World Health Organization, 2016.
61. Wasserheit JN. Epidemiological synergy. Interrelationships between human immunodeficiency virus infection and other sexually transmitted diseases. *Sex Transm Dis* 1992; **19**(2): 61-77.
62. Rietmeijer CA, Mungati M, Machiha A, et al. The Etiology of Male Urethral Discharge in Zimbabwe: Results from the Zimbabwe STI Etiology Study. *Sex Transm Dis* 2018; **45**(1): 56-60.
63. Kilmarx PH, Gonese E, Lewis DA, et al. HIV infection in patients with sexually transmitted infections in Zimbabwe - Results from the Zimbabwe STI etiology study. *PLoS One* 2018; **13**(6): e0198683.
64. Guidelines for the management of sexually transmitted infections Geneva, Switzerland: World Health Organizatinon, 2004.
65. Mungati M, Machiha A, Mugurungi O, et al. The Etiology of Genital Ulcer Disease and Coinfections With Chlamydia trachomatis and Neisseria gonorrhoeae in Zimbabwe: Results From the Zimbabwe STI Etiology Study. *Sex Transm Dis* 2018; **45**(1): 61-8.
66. Avuvika E, Masese LN, Wanje G, et al. Barriers and Facilitators of Screening for Sexually Transmitted Infections in Adolescent Girls and Young Women in Mombasa, Kenya: A Qualitative Study. *PloS one* 2017; **12**(1): e0169388-e.
67. Ferreira A, Young T, Mathews C, Zunza M, Low N. Strategies for partner notification for sexually transmitted infections, including HIV. *Cochrane Database Syst Rev* 2013; (10): CD002843.
68. Kalichman SC, Mathews C, Kalichman M, Lurie MN, Dewing S. Perceived barriers to partner notification among sexually transmitted infection clinic patients, Cape Town, South Africa. *Journal of public health (Oxford, England)* 2017; **39**(2): 407-14.
69. Offorjebe OA, Wynn A, Moshashane N, et al. Partner notification and treatment for sexually transmitted infections among pregnant women in Gaborone, Botswana. *Int J STD AIDS* 2017; **28**(12): 1184-9.
70. Alam N, Chamot E, Vermund SH, Streatfield K, Kristensen S. Partner notification for sexually transmitted infections in developing countries: a systematic review. *BMC Public Health* 2010; **10**: 19.
71. Mathews C, Kalichman MO, Laubscher R, et al. Sexual relationships, intimate partner violence and STI partner notification in Cape Town, South Africa: an observational study. *Sex Transm Infect* 2018; **94**(2): 144-50.
72. Saunders B, Sim J, Kingstone T, et al. Saturation in qualitative research: exploring its conceptualization and operationalization. *Quality & quantity* 2018; **52**(4): 1893-907.
73. Braun V, Clarke V. Using thematic analysis in psychology. *Qualitative Research in Psychology* 2006; **3**(2): 77-101.
74. Lu W, Yanos PT, Silverstein SM, et al. Public mental health clients with severe mental illness and probable posttraumatic stress disorder: trauma exposure and correlates of symptom severity. *J Trauma Stress* 2013; **26**(2): 266-73.
75. Lewis SJ, Arseneault L, Caspi A, et al. The epidemiology of trauma and post-traumatic stress disorder in a representative cohort of young people in England and Wales. *Lancet Psychiatry* 2019; **6**(3): 247-56.

76. Prevention and Promotion in Mental Health. Geneva, Switzerland: World Health Organization, 2002.
77. Patel V, Araya R, de Lima M, Ludermir A, Todd C. Women, poverty and common mental disorders in four restructuring societies. *Soc Sci Med* 1999; **49**(11): 1461-71.
78. Lund C, Breen A, Flisher AJ, et al. Poverty and common mental disorders in low and middle income countries: A systematic review. *Soc Sci Med* 2010; **71**(3): 517-28.
79. Fegert JM, Vitiello B, Plener PL, Clemens V. Challenges and burden of the Coronavirus 2019 (COVID-19) pandemic for child and adolescent mental health: a narrative review to highlight clinical and research needs in the acute phase and the long return to normality. *Child Adolesc Psychiatry Ment Health* 2020; **14**: 20.
80. Sommer M, Cherenack E, Blake S, M. S, Burgers L. WASH in Schools Empowers Girls' Education: Proceedings of the Menstrual Hygiene Management in Schools Virtual Conference 2014. New York, USA: United Nations Children's Fund and Columbia University, 2015.
81. Bundy DAP, de Silva N, Horton S, Patton GC, Schultz L, Jamison DT. Investment in child and adolescent health and development: key messages from Disease Control Priorities, 3rd Edition. *Lancet* 2018; **391**(10121): 687-99.
82. WHO. Health for the World's Adolescents: Asecond chance in the second decade. Geneva: World Health Organization 2014.
83. Suthar AB, Lawn SD, del Amo J, et al. Antiretroviral therapy for prevention of tuberculosis in adults with HIV: a systematic review and meta-analysis. *PLoS Med* 2012; **9**(7): e1001270.
84. Weijer C, Emanuel EJ. Ethics. Protecting communities in biomedical research. *Science* 2000; **289**(5482): 1142-4.
85. Youth and HIV. Mainstreaming a three-lens approach to youth participation. Geneva, Switzerland: UNAIDS, 2018.
86. Guidelines for Conducting Adolescent HIV Sexual and Reproductive Health Research in Kenya: National AIDS and STI Control Programme (NASCOP) & Kenya Medical Research Institute (KEMRI), 2015.
87. Guidance on ethical considerations in planning and reviewing research studies on sexual and reproductive health in adolescents. Geneva, Switzerland: World Health Organization, 2018.
